# Correction: DNAJB1-PRKACA fusion protein-regulated LINC00473 promotes tumor growth and alters mitochondrial fitness in fibrolamellar carcinoma

**DOI:** 10.1371/journal.pgen.1012054

**Published:** 2026-02-26

**Authors:** Rosanna K. Ma, Pei-Yin Tsai, Alaa R. Farghli, Alexandria Shumway, Matt Kanke, John D. Gordan, Taranjit S. Gujral, Khashayar Vakili, Manabu Nukaya, Leila Noetzli, Sean Ronnekleiv-Kelly, Wendy Broom, Joeva Barrow, Praveen Sethupathy

Following publication of this article [1], the following errors were noted in Fig 4, S3 Fig, and in the Materials and methods:

- In Fig 4H, the LeGO-Ctl TUNEL panel is incorrect and is a duplicate of the sh473 TUNEL panel; additionally, the sh473 TUNEL and LeGO-473ox TUNEL panels are mistakenly switched. The first and corresponding authors confirmed this was due to accidental copy and paste errors in the creation of the full figure which do not affect any calculations or conclusions related to this figure.
- In the bar chart of Fig 4H (*Knockdown*), the standard deviation for the RQV of % TUNEL+ sh473 cells is miscalculated and the resulting error bar for sh473 cells is incorrect. The correct standard deviation is 3.614. The authors confirm the p-value and means reported on this graph are correct.
- In S3D Fig, the n value provided in the legend text is incorrect. The correct n value for panel S3D is 1 and is updated in the revised S3 Fig legend provided with this Correction. The content of S3 Fig is unaffected.
- In the 2. PDX tumors section of the Materials and methods, there is an error in the fifth sentence of the third paragraph and the humane endpoint used in the animal study is inaccurately described.

With this Correction, the authors provide a revised Fig 4 in which the incorrect panels are replaced with the correct images from the original experiments and the error bar in the Fig 4H (*Knockdown*) bar chart is also corrected.

The original images underlying Figs 4 and S3C are shared in S1-S3 Files. Individual-level quantitative data from the original experiments are available for Figs 4, 5, 6A-B, and S3D and are included here in S4-S6 Files.

The fifth and sixth sentences of the third paragraph in the 2. PDX tumors section of the Materials and methods are corrected to the following: Mice are monitored daily for signs of distress, such as poor grooming and weight loss until the endpoint, which is established when animals exhibit 20% body weight loss or greater, tumor burden starts to ulcerate, or animal body condition deteriorates with visible signs of pain/distress; animals are unable to sleep, eat or ambulate. Tumor size will be monitored and euthanasia is determined in consultation with CARE veterinarians. Mice are subsequently euthanized using carbon dioxide administration for tumor collection, which is divided for propagating tumor passaging. Individual animals are placed into a clean cage and are administered carbon dioxide at a flow rate of 3.5 L/min. Mice are removed from the cage after respirations cease for a period of one minute.

**Fig 4:**
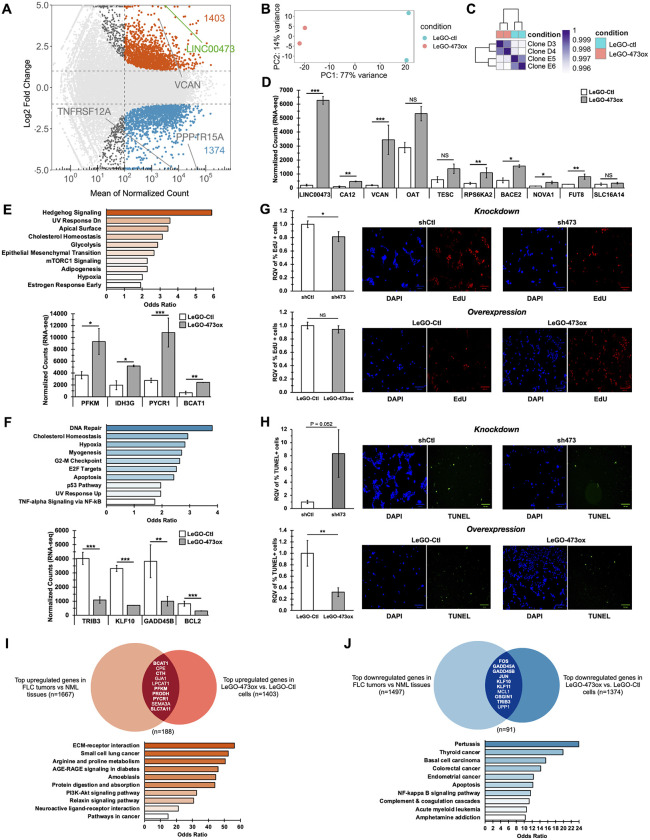
LINC00473 increases FLC cell survival by suppressing apoptosis. **(A)** MA plot showing differentially expressed genes in FLC cells that stably overexpress LINC00473 (LeGO-473ox) relative to empty vector control (LeGO-Ctl). Genes filtered for expression with base mean > 100, log2FC > 0.5 or <-0.5, and padj < 0.05 (DESeq). Dashed lines represent log2 FC of 1 and -1 (horizontal) and mean normalized count = 100 (vertical). Up- and down-regulated genes are colored orange or blue, respectively. **(B)** Principal component analysis of DESeq normalized rlog counts with cell treatment information shown by color. LeGO-Ctl (n = 2), and LeGO-473ox (n = 2) are shown in orange and blue, respectively. **(C)** Hierarchical clustering analysis of gene expression profiles across samples using DESeq normalized rlog counts. FLC cells with LINC00473 overexpression (LeGO-473ox) or empty vector control (LeGO-Ctl) are indicted by orange and blue boxes, respectively. **(D)** Normalized counts for specific FLC marker genes from RNA-seq from LeGO-473ox and LeGO-Ctl control FLC monoclonal cells. **(E, F)** MSigDB Hallmark pathway enrichment analysis of significantly upregulated genes (n = 1403) (A) and downregulated genes (n = 1374) **(B)**, respectively. Normalized counts for metabolism-related genes from RNA-seq from LeGO-473ox and LeGO-Ctl control FLC monoclonal cells. **(G)** EdU incorporation in FLC cells with stable LINC00473 knockdown (sh473) compared to control (shCtl), and LINC00473 overexpression (LeGO-473ox) compared to control (LeGO-Ctl). Representative images of DAPI- and EdU-stained cells show total and proliferative cells, respectively. **(H)** TUNEL staining in FLC cells with stable LINC00473 knockdown (sh473) compared to control (shCtl), and LINC00473 overexpression (LeGO-473ox) compared to control (LeGO-Ctl). Representative images of DAPI- and TUNEL-stained cells show total and apoptotic cells, respectively. **(I)** Gene list overlap analysis using significantly upregulated genes in FLC tumors relative to NML (n = 1667), and in LeGO-473ox cells relative to control (n = 1403). Bolded genes indicate proteins related to metabolism. KEGG pathway enrichment analysis of the intersecting 188 genes. Significance of the overlap (p = 4.81x10^-20^) was calculated by hypergeometric test. **(J)** Gene list overlap analysis using significantly downregulated genes in FLC tumors relative to NML (n = 1497), and in LeGO-473ox cells relative to control (n = 1374). Bolded genes indicate proteins related to apoptosis. KEGG pathway enrichment analysis of the intersecting 91 genes. Overlap of n = 91 genes was not significant following a hypergeometric test. Bar chart data in G and H are represented as mean across n = 6 ± SD. Relative Quantitative Values represent the change of any normalized measurement relative to the control group. Pathway enrichment figures indicate pathways with p-values < 0.05 and bar color intensity represents odds ratio. Scale bars represent 100 μm. P values are calculated by 2-tailed Student’s t-test. *p < 0.05, **p < 0.01, ***p < 0.001 unless otherwise indicated.

## Supporting information

S1 File Fig 4G underlying images. This file includes the original images underlying Fig 4G; Representative images of DAPI- and EdU-stained FLC cells.
(ZIP)

S2 File Fig 4H underlying images. This file includes the original images underlying Fig 4H; Representative images of DAPI- and TUNEL-stained FLC cells.
(ZIP)

S3 File Fig S3C underlying images. This file includes the original images underlying Fig S3C; Representative immunoblot of protein expression of DNAJB1- PRKACA (DP) fusion is detected with a protein kinase A catalytic α subunit (PKA) antibody. Lane 1, siDP#1-LNP; Lane 2, siDP#2-LNP; Lane 3, siLuciferase (siLuc-LNP) negative control; Lane 4, mock negative controls following 250nM treatment.
(ZIP)

S4 File Fig 4 underlying quantitative data. This file includes the original quantitative data underlying Fig 4; (A) RNA-seq data from FLC cells that stably overexpress LINC00473 (LeGO-473ox) relative to empty vector control (LeGO-Ctl). These data are used to plot Figs 4A-4F, and 4I-4J. (B) Fig 4D-4F: Normalized counts for specific FLC marker genes from RNA-seq from LeGO-473ox and LeGO-Ctl control FLC monoclonal cells. (C) Fig 4E: MSigDB Hallmark pathway enrichment analysis of significantly upregulated genes (n = 1403). (D) Fig 4F: MSigDB Hallmark pathway enrichment analysis of significantly downregulated genes (n = 1374). (E) Fig 4G: Quantification of EdU incorporation in FLC cells with stable LINC00473 knockdown (sh473) or overexpression (LeGO-473ox) relative to their respective controls (shCtl, LeGO-Ctl). (F) Fig 4H: Quantification of TUNEL staining in FLC cells with stable LINC00473 knockdown (sh473) or overexpression (LeGO-473ox) relative to their respective controls (shCtl, LeGO-Ctl). (G) Fig 4I: Gene list overlap analysis using significantly upregulated genes in FLC tumors relative to NML (n = 1667), and in LeGO-473ox cells relative to control (n = 1403). Bolded genes indicate proteins related to metabolism. Includes KEGG pathway enrichment analysis of the intersecting 188 genes. Significance of the overlap (p = 4.81x10^-20^) was calculated by hypergeometric test. (H) Fig 4J: Gene list overlap analysis using significantly downregulated genes in FLC tumors relative to NML (n = 1497), and in LeGO-473ox cells relative to control (n = 1374). Bolded genes indicate proteins related to apoptosis. Includes KEGG pathway enrichment analysis of the intersecting 91 genes. Overlap of n = 91 genes was not significant following a hypergeometric test.
(ZIP)

S5 File Fig 5 underlying quantitative data. This file includes the original quantitative data underlying Fig 5; (A) Fig 5A: Representative graph of extracellular acidification rate (ECAR) of FLC cells with stable LINC00473 overexpression (LeGO-473ox) and empty vector control (LeGO-Ctl). (B) Fig 5B: Quantification of glycolysis, glycolytic capacity and glycolytic reserve in LINC00473-overexpression (LeGO-473ox) FLC cells compared to empty-vector control (LeGO-Ctl). (C) Fig 5C: Representative graph of ECAR of FLC cells with stable LINC00473 knockdown (sh473) and non-targeting control (shCtl). (D) Fig 5D: Quantification of glycolysis, glycolytic capacity and glycolytic reserve in LINC00473-knockdown (sh473) FLC cells compared to non-targeting control (shCtl). (E) Fig 5E: Representative graph of oxygen consumption rate (OCR) of FLC cells with overexpression (LeGO-473ox) and control (LeGO-Ctl). (F) Fig 5F: Quantification of basal respiration, maximum respiration capacity, and spare respiratory capacity in LINC00473-overexpression (LeGO-473ox) FLC cells compared to empty-vector control (LeGO-Ctl). (G) Fig 5G: Representative graph of OCR of FLC cells with stable LINC00473 knockdown (sh473) and non-targeting control (shCtl). (H) Fig 5H: Quantification of basal respiration, maximum respiration capacity, and spare respiratory capacity in LINC00473-knockdown (sh473) FLC cells compared to non-targeting control (shCtl).
(ZIP)

S6 File Fig 6A-B underlying quantitative data. This file includes the original quantitative data underlying Fig 6A-B. Tumor length and width data are expressed in mm units and tumor volume as mm^3^.
(XLSX)

S7 File Fig S3D underlying quantitative data. This file includes the original quantitative data underlying Fig S3D; fold change and densitometry of immunoblot of protein expression of DP fusion and WT PKA via densitometry of the blot in Fig S3C, relative to siLuc negative control (n = 1).
(XLSX)

S3 Fig Silencing of the DP fusion. **(A, C)** Representative immunoblot of protein expression of DNAJB1- PRKACA (DP) fusion is detected with a protein kinase A catalytic α subunit (PKA) antibody. WT PKAc, DP fusion major, and DP fusion minor are identified. Lane 1, siDP#1-LNP; Lane 2, siDP#2-LNP; Lane 3, siLuciferase (siLuc-LNP) negative control; Lane 4, mock negative controls following 1.25nM treatment (A) or 2.50nM treatment (C) with siRNA-LNPs or mock condition over 96 hours. Vinculin loading control is shown in the lower panel and run on the same blot**. (B, D)** Fold change of protein levels of the blot in panel A (B) and panel C (D), relative to siLuc negative control (n = 1). **(E)** Representative immunoblot of protein expression of WT DNAJB1. Lane 1, siDP#1-LNP; Lane 2, siDP#2-LNP; Lane 3, siLuciferase (siLuc-LNP) negative control; Lane 4, mock negative control. siRNA-LNP treatments at 5nM, or mock condition, over 96 hours. Vinculin loading control is shown in the lower panel and run on the same blot (n = 3)**. (F)** Gene expression from RT-qPCR following free uptake of siDP#1-LNP, siDP#2-LNP, and siLuc-LNP at 5nM treatment over 96 hours in FLC cells, as shown in Fig 3F (n = 3). Data are represented as mean ± SD. P values are calculated by 2-tailed Student’s t-test. *p < 0.05, **p < 0.01, ***p < 0.001.
(PDF)
