# Supplementary figures and images for: DNAJB1-PRKACA fusion protein-regulated LINC00473 promotes tumor growth and alters mitochondrial fitness in fibrolamellar carcinoma
Source: PLoS Genet. 2024 Mar 21;20(3):e1011216. doi: 10.1371/journal.pgen.1011216 (PMC11020935; doi:10.1371/journal.pgen.1011216)

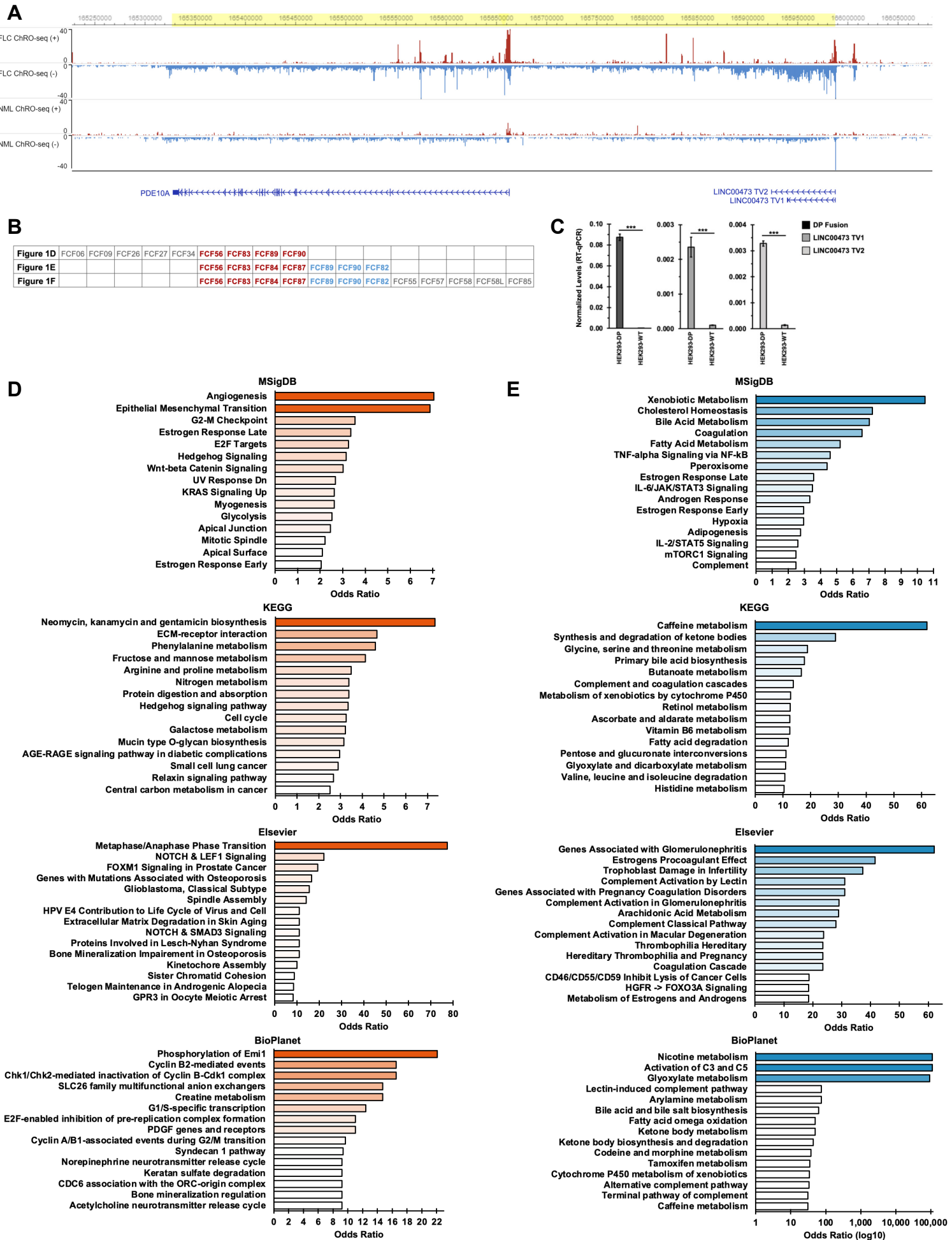

Supplementary Figure 1. LINC00473 is a distinct transcription unit in FLC tumors.

Supplement: S1 Fig — (A) Genome snapshot of the LINC00473 and PDE10A loci on chr6:165250000–166050000. Transcriptional signal on the plus and minus strand are shown in red and blue, respectively, in FLC tumors and non-malignant liver (NML) tissues. The RefSeq annotations for LINC00473 TV1 and TV2, and PDE10A, are shown on the bottom panel. Both genes are transcribed on the minus strand. LINC00473 has markedly higher levels of gene body transcription than the space in between LINC00473 and PDE10A. (B) Lists of primary patient tumors used in RNA-seq counts in a patient-matched subset from Cohort 1, gene expression via RT-qPCR in a patient- matched subset from Cohort 1, and correlation analyses on RNA expression levels of the DP fusion and LINC00473 isoforms TV1 and TV2 using patient tumors from Cohort 1, as described in Fig 1D, 1E and 1F, respectively. Tumors indicated in red are common for all three analyses, and tumors highlighted in blue are shared for Fig 1E and 1F. (C) Normalized levels of DP fusion and LINC00473 isoforms TV1 and TV2 RNA in HEK293-DP cells relative to wild-type control (HEK293-Ctl). Normalized levels are 2^(-dCt) values using RPS9 for normalization and presented from 3 technical replicates. Data are represented as mean ± SD. P values are calculated by 2-tailed Student’s t-test. *p < 0.05, **p < 0.01, ***p < 0.001. (D, E) Pathway enrichment analyses of significantly upregulated genes (n = 1666) (A) and downregulated genes (n = 1497) (B), respectively, in primary FLC tumors (n = 35) relative to non-malignant tissue (n = 10). Genes filtered for expression with base mean > 100, log2FC > 1 or < -1, and padj < 0.05 (DESeq). Pathways with adjusted p-value < 0.05 represented in figure. Color intensity represents odds ratio value. (PDF) [file pgen.1011216.s001.pdf]

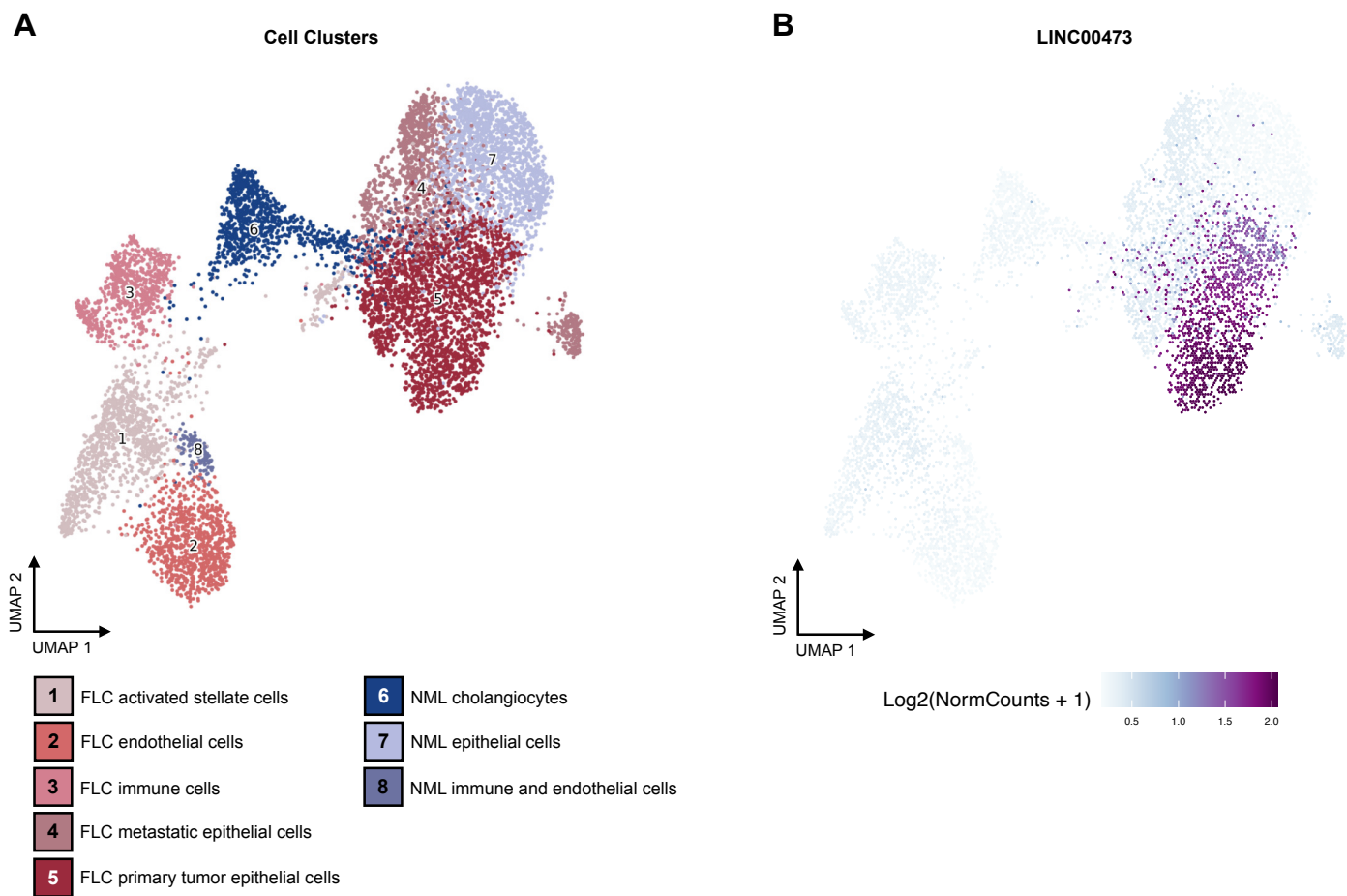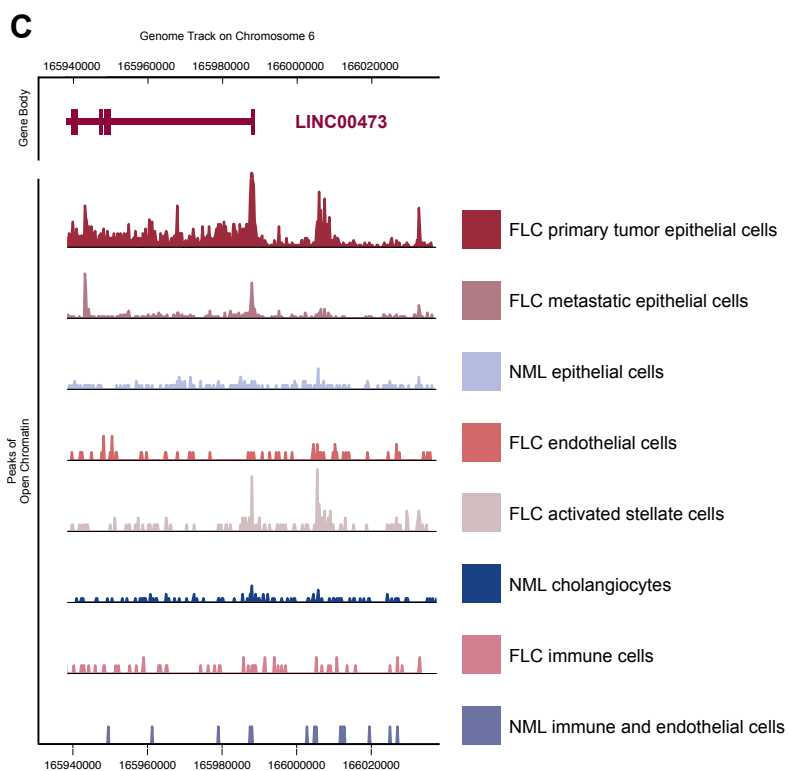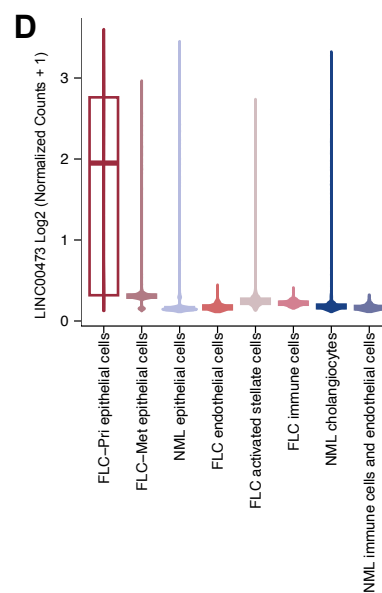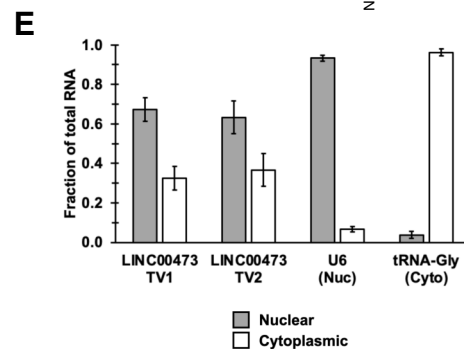

Supplementary Figure 2. LINC00473 is enriched in FLC tumor epithelial cells.

Supplement: S2 Fig — (A) snATAC-seq UMAP plot demonstrating eight cell clusters found in primary FLC, metastatic FLC, and NML tissue (~9500 nuclei). (B) Single-nucleus analysis of chromatin accessibility near the LINC00473 locus. Increasing signal is indicated by the color gradient (maximum signal is dark purple and minimal signal is light blue). (C) Top panel depicts the gene body of LINC00473, which is transcribed on the minus strand. Bottom panel demonstrates the quantification of snATAC-seq chromatin accessibility signal near the LINC00473 locus in different cell types. (D) Boxplot showing normalized counts of chromatin accessibility signal of LINC00473 in distinct cell types. (E) Subcellular fractionation followed by RT-qPCR in FLC cells using TaqMan primers designed to detect variants 1 (TV1) and 2 (TV2) of LINC00473, U6 (nuclear control) and tRNA-Gly (cytoplasmic control). Data represents n = 3 biological replicates ± SD. For A-D, data are represented from n = 1 FLC tumor, 1 Metastatic tumor and 1 NML. (PDF) [file pgen.1011216.s002.pdf]

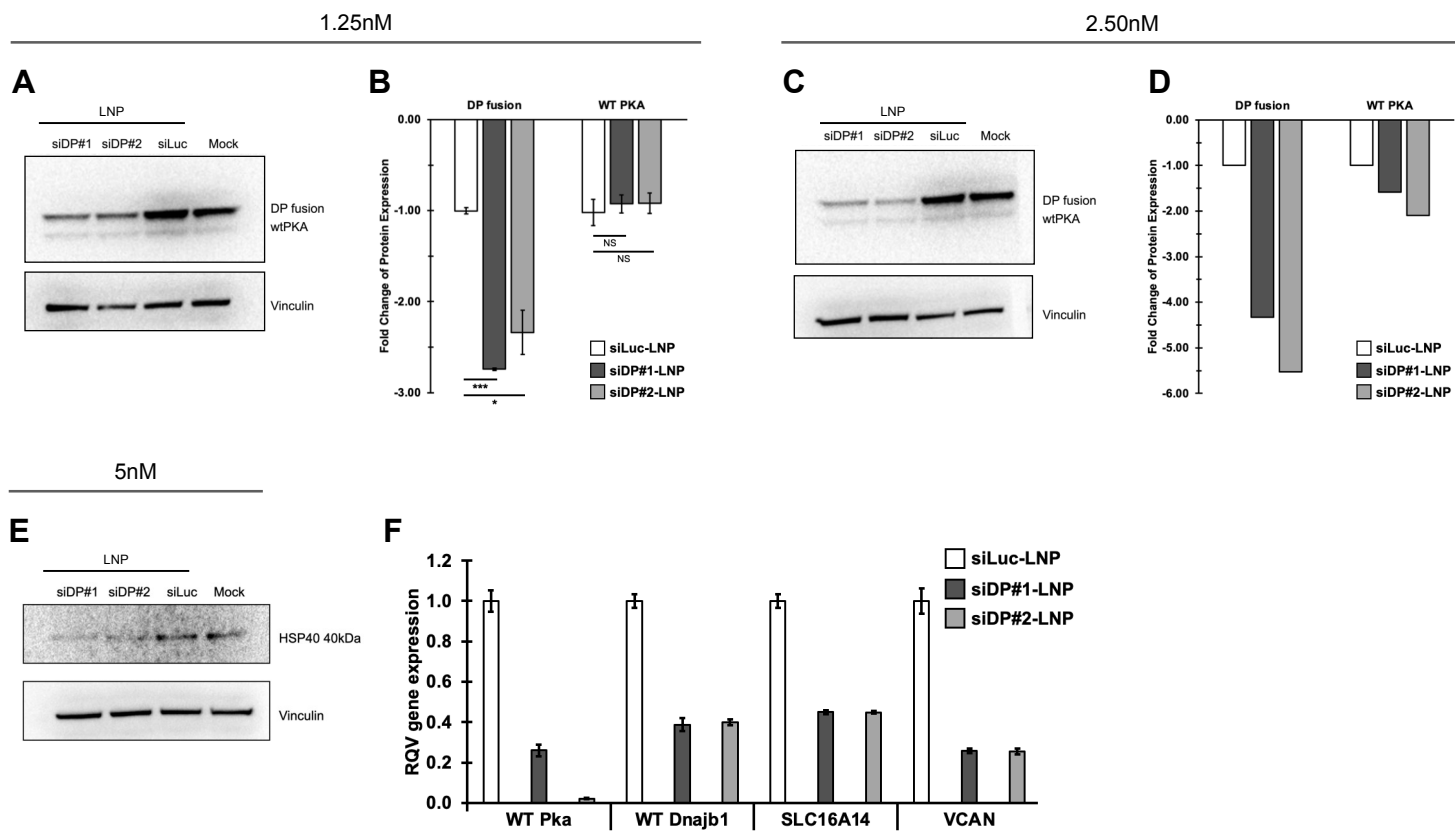

Supplementary Figure 3. Silencing of the DP fusion.

Supplement: S3 Fig — (A, C) Representative immunoblot of protein expression of DNAJB1- PRKACA (DP) fusion is detected with a protein kinase A catalytic α subunit (PKA) antibody. WT PKAc, DP fusion major, and DP fusion minor are identified. Lane 1, siDP#1-LNP; Lane 2, siDP#2-LNP; Lane 3, siLuciferase (siLuc-LNP) negative control; Lane 4, mock negative controls following 1.25nM treatment (A) or 2.50nM treatment (C) with siRNA-LNPs or mock condition overs 96 hours. Vinculin loading control is shown in the lower panel and run on the same blot. (B, D) Fold change of protein levels of the blot in panel A (B) and panel C (D), relative to siLuc negative control (n = 3). (E) Representative immunoblot of protein expression of WT DNAJB1. Lane 1, siDP#1-LNP; Lane 2, siDP#2-LNP; Lane 3, siLuciferase (siLuc-LNP) negative control; Lane 4, mock negative control. siRNA-LNP treatments at 5nM, or mock condition, over 96 hours. Vinculin loading control is shown in the lower panel and run on the same blot (n = 3). (F) Gene expression from RT-qPCR following free uptake of siDP#1-LNP, siDP#2-LNP, and siLuc-LNP at 5nM treatment over 96 hours in FLC cells, as shown in Fig 3F (n = 3). Data are represented as mean ± SD. P values are calculated by 2-tailed Student’s t-test. *p < 0.05, **p < 0.01, ***p < 0.001. (PDF) [file pgen.1011216.s003.pdf]
