# Supplementary material for: DNAJB1-PRKACA fusion protein-regulated LINC00473 promotes tumor growth and alters mitochondrial fitness in fibrolamellar carcinoma
Source: PLoS Genet. 2024 Mar 21;20(3):e1011216. doi: 10.1371/journal.pgen.1011216 (PMC11020935; doi:10.1371/journal.pgen.1011216)
Supplement: S4 Fig — (A) LINC00473 TV1 expression from RT-qPCR in FLC monoclonal cells following limiting dilutions of polyclonal cell lines to isolate single-clone cell colonies with stable gene knockdown using two independent shRNAs (sh473-2, sh473-4) or non-targeting shRNA control (shCtl) (n = 3), or overexpression using cDNA plasmid encoding LINC00473 TV1 (LeGO-473ox) or empty- vector control (LeGO-Ctl) (n = 3). (B) Expression of LINC00473 TV2 was queried via RT-qPCR in FLC monoclones described in panel A (n = 3). (C) LINC00473 TV2 expression from RT-qPCR in FLC polyclones induced with LINC00473 TV1 overexpression (n = 3). (D) Expression of LINC00473 TV1 from RT-qPCR in HEK293-DP polyclonal cells following lentiviral transfection enabling stable gene knockdown using two independent shRNAs (sh473-2, sh473-4) or non-targeting shRNA control (shCtl) (n = 3) or overexpression using cDNA plasmid encoding LINC00473 TV1 (LeGO-473ox) or empty-vector control (LeGO-Ctl) (n = 3). The expression of second variant of LINC00473 (TV2) in HEK293-DP cells with stable sh473-2, sh473-4, or shCtl (n = 3). (E) Cell growth curve of HEK293-DP monoclonal cells with stable LINC00473 knockdown (sh473-2: clones F3, F4; sh473-4: clones G1, G3) and non-targeting control (shCtl: clones H4, H5). Each monoclonal cell line was quantified 2 times across independent passages, and each point is the average cell count of 3 replicates. (F) Expression levels of both LINC00473 isoforms from RT-qPCR in HEK293-DP monoclones with stable gene knockdown using two independent shRNAs (sh473-2, sh473-4) or non-targeting shRNA control (shCtl). Data are represented as mean across 3 replicates ± SD. P values are calculated by 2-tailed Student’s t-test. *p < 0.05, **p < 0.01, ***p < 0.001. (PDF) [file pgen.1011216.s004.pdf]

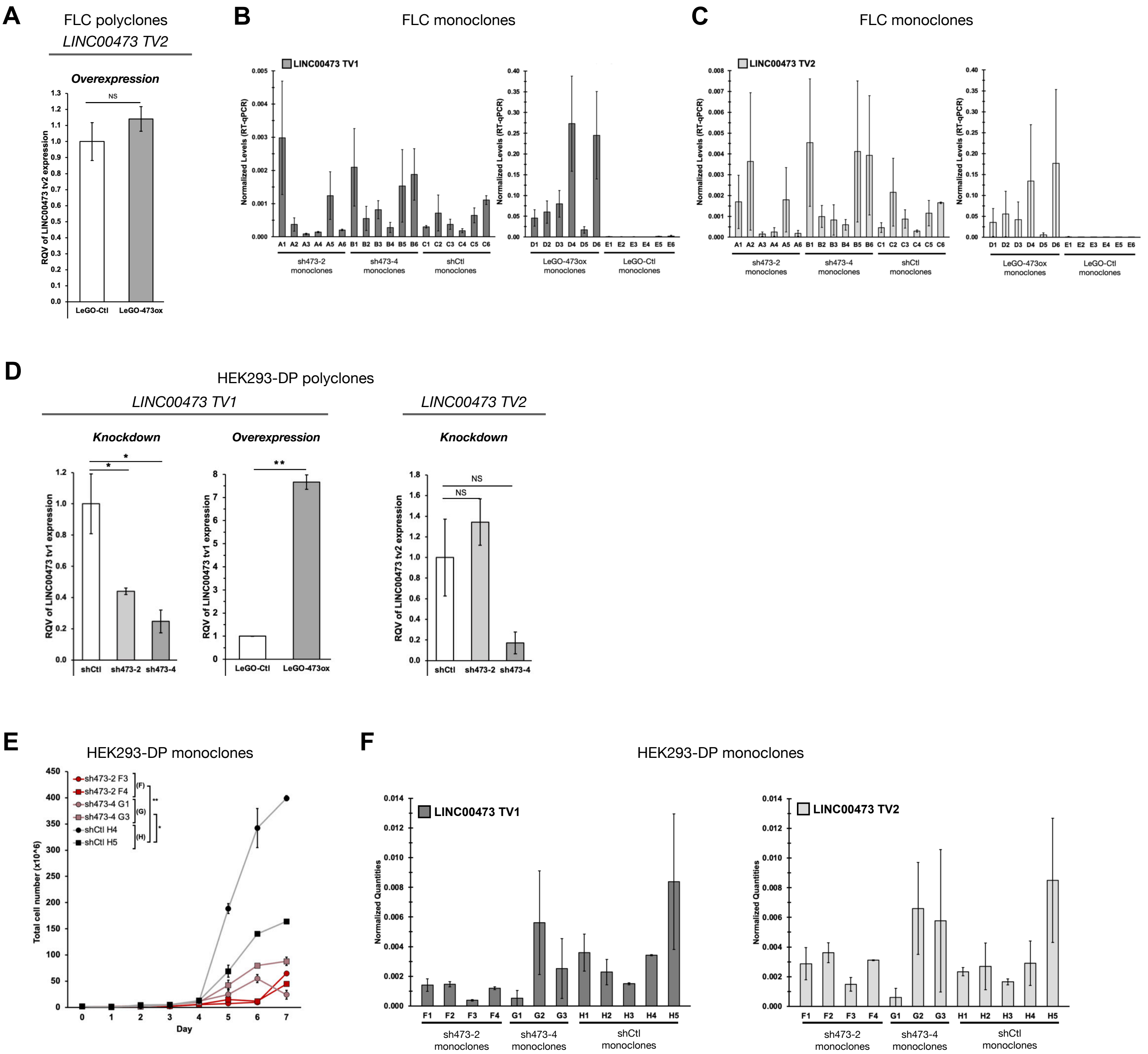

Supplementary Figure 4. LINC00473 is efficiently downregulated or overexpressed in FLC and HEK293-DP cells.
