# Supplementary material for: DNAJB1-PRKACA fusion protein-regulated LINC00473 promotes tumor growth and alters mitochondrial fitness in fibrolamellar carcinoma
Source: PLoS Genet. 2024 Mar 21;20(3):e1011216. doi: 10.1371/journal.pgen.1011216 (PMC11020935; doi:10.1371/journal.pgen.1011216)
Supplement: S5 Fig — (A) Normalized counts of several genes of interest from RNA-seq of LeGO-473ox and LeGO-Ctl control FLC monoclonal cells. Bars are ± SD. The panel includes FLC-relevant transcriptional regulators (MYC), or genes associated with cellular metabolism (IDH1, PYCR1) and cell survival (XIAP, CDK6, MALAT1). (B) Pathway analyses using the 1403 upregulated genes in FLC monoclones with LINC00473 overexpression (LeGO-473ox) relative to empty vector control (LeGO-Ctl). Genes were filtered for expression with base mean > 100, log2FC > 1 and padj < 0.05 (DESeq). (C) Pathway analyses using the 1374 downregulated genes in FLC monoclones with LeGO-473ox relative to LeGO-Ctl. Genes were filtered for expression with base mean > 100, log2FC < 1 and padj < 0.05 (DESeq). (D, E) Gene list overlap analysis using significantly up- (D) or down-regulated (E) genes in FLC tumors relative to NML (n = 1497), and in LeGO-473ox cells relative to control (n = 1374). Pathways with p-value < 0.05 represented in figure. Color intensity represents odds ratio value. P values are calculated by 2-tailed Student’s t-test. *p < 0.05, **p < 0.01, ***p < 0.001. (PDF) [file pgen.1011216.s005.pdf]

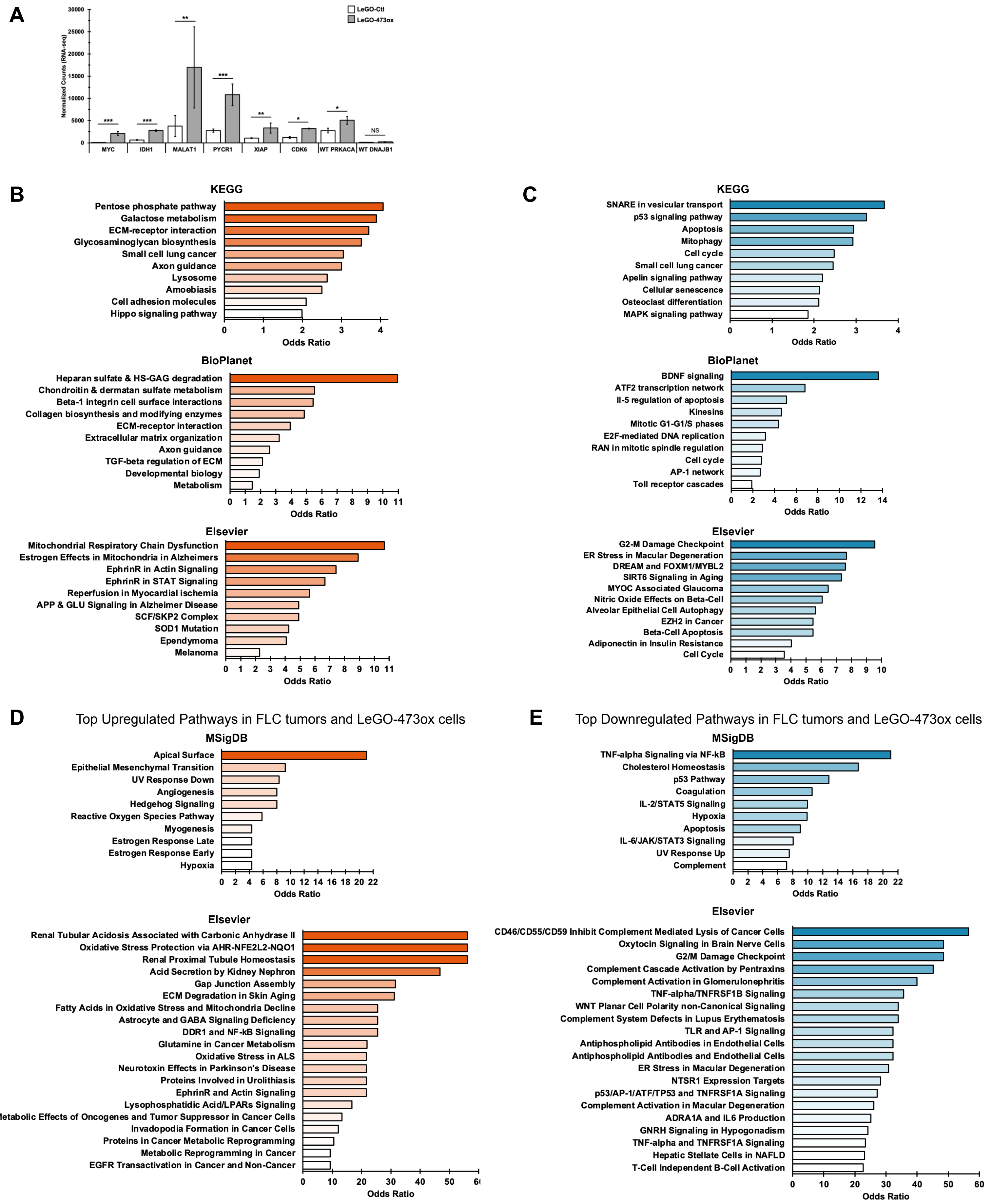

Supplementary Figure 5. LINC00473 upregulates genes enriched in metabolism pathways and downregulates pathways related to apoptosis.
