# Supplementary material for: DNAJB1-PRKACA fusion protein-regulated LINC00473 promotes tumor growth and alters mitochondrial fitness in fibrolamellar carcinoma
Source: PLoS Genet. 2024 Mar 21;20(3):e1011216. doi: 10.1371/journal.pgen.1011216 (PMC11020935; doi:10.1371/journal.pgen.1011216)
Supplement: S6 Fig — (A) Expression of FLC-related genes from RT-qPCR in LINC00473-overexpression (LeGO-473ox) and empty-vector control (LeGO-Ctl) subcutaneous FLC tumors. (B) KEGG pathway analyses using the 955 upregulated genes in FLC monoclones with LINC00473 overexpression (LeGO-473ox) relative to empty vector control (LeGO-Ctl). Genes were filtered for expression with base mean > 100, log2FC > 1 and padj < 0.05 (DESeq). (C) KEGG pathway analyses using the 1468 downregulated genes in FLC monoclones with LeGO-473ox relative to LeGO-Ctl. Genes were filtered for expression with base mean > 100, log2FC < 1 and padj < 0.05 (DESeq). Pathways with p-value < 0.05 represented in figure. Color intensity represents odds ratio value. P values are calculated by 2-tailed Student’s t-test. *p < 0.05, **p < 0.01, ***p < 0.001. (PDF) [file pgen.1011216.s006.pdf]

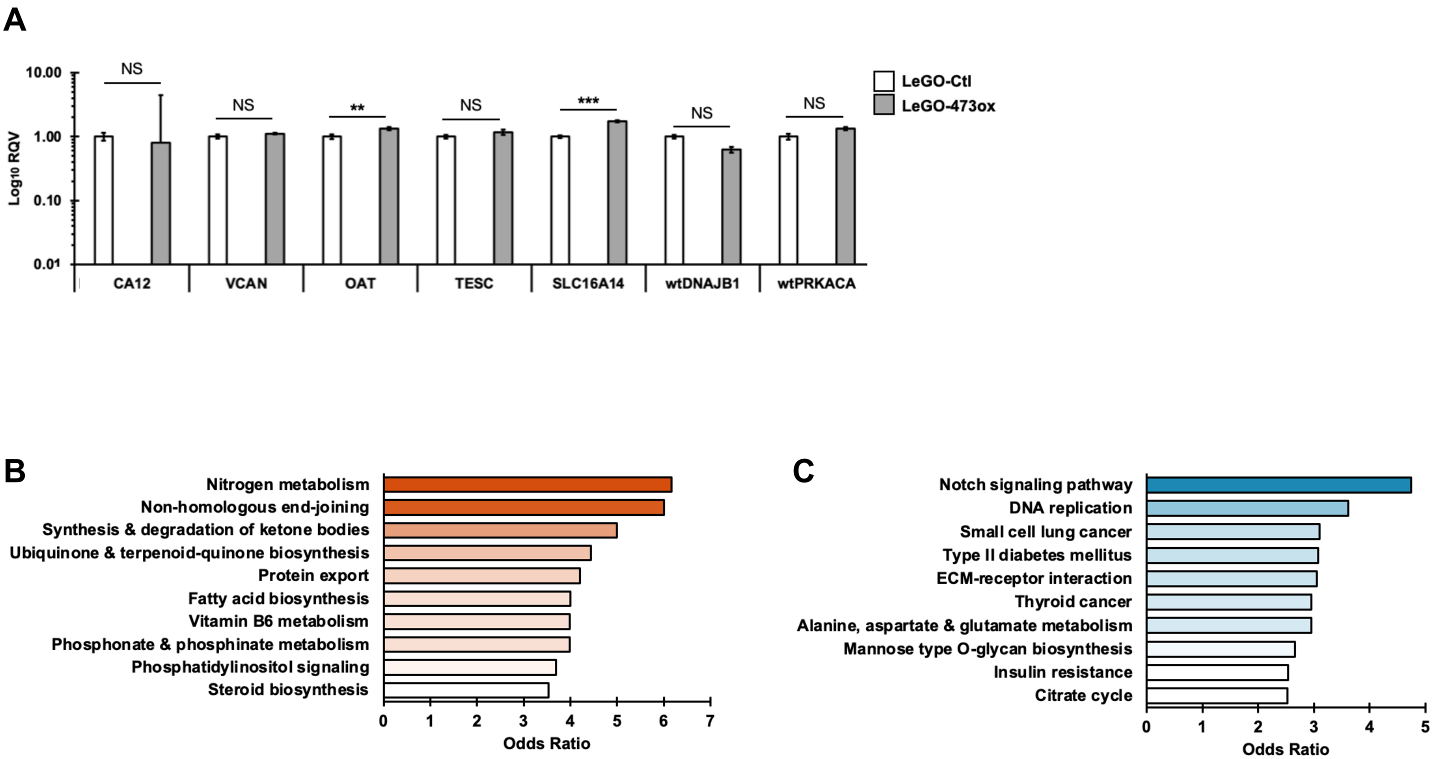

Supplementary Figure 6. Gene expression in tumors generated from LINC00473 overexpressing cell implantation.
