# Supplementary material for: DNAJB1-PRKACA fusion protein-regulated LINC00473 promotes tumor growth and alters mitochondrial fitness in fibrolamellar carcinoma
Source: PLoS Genet. 2024 Mar 21;20(3):e1011216. doi: 10.1371/journal.pgen.1011216 (PMC11020935; doi:10.1371/journal.pgen.1011216)
Supplement: Attachment — Submitted filename: Response to Reviewers_PLOS Genetics.pdf [file pgen.1011216.s011.pdf]

Editor's comments:

The reviewers all find this manuscript potentially appropriate for PLOS Genetics. Please consider the reviewer comments and plan to address each comment in a point-by-point response if you choose to resubmit your work.

We are very pleased by the enthusiasm for the work and overall very positive review. We have considered each of the Reviewers' points very carefully and have provided point-by-point responses below.

Comments to the Reviewers:

Reviewer #1:
In their manuscript entitled, "DNAJB1-PRKACA fusion protein-regulated LINC00473 promotes tumor growth and alters mitochondrial fitness in fibrolamellar carcinoma", Ma and colleagues investigate the mechanism of action of LINC00473 in the context of the DNAJB1-PRKACA fusion that drives fibrolamellar carcinoma (FLC), a rare but deadly cancer.

The authors generate primary RNA-Seq data from FLC patients and matched non-malignant liver samples. This dataset is a valuable community resource given the rare nature of this cancer type, and the authors have duly deposited the data into GEO and other repositories. Next, the authors establish using CRISPR-Cas9 deletion models as well as DNAJB1-PRKACA knockout models that LINC00473 is regulated by the fusion protein. They then perform gain-of-function and loss-of-function studies followed by RNA-Seq and molecular pathway analysis to predict affected biological pathways. Finally, an exploration of oncogenic mechanisms is explored using metabolic assays and xenograft models.

The manuscript is well written and clear. The experiments appeared to be well designed, and the data are generally convincing.

My biggest criticism of the manuscript in its current form is that I believe more could be done to build a case that LINC00473 is the major downstream target of the DNAJB1-PRKACA fusion. In the current manuscript, the authors cite literature evidence and prior work showing that LINC00473 has already been established as an oncogene. However, the authors generate a cohort of RNA-Seq from FLC and NML that could potentially be used in a hypothesis generating fashion. How do we know that LINC00473 is the most important target of the DNAJB1-PRKACA fusion protein? What other oncogenic mechanisms might be at play here?

These questions are not meant to detract from the well-designed experiments that the authors perform in the manuscript. However, a more thorough RNA-Seq analysis would enhance the manuscript's overall impact. Some of these thoughts are echoed below as specific points.

Thank you to the reviewers and editors for the opportunity to serve as a reviewer.

We appreciate the Reviewer's positive feedback on our manuscript. Please find below our responses to each specific point raised.

Major points:
1. It appears that the RNA-Seq dataset is being used in a hypothesis-driven rather than a hypothesis generating manner. The authors cite the literature support for LINC00473 to motivate the study as it has been studied in several cancer contexts, but the RNA-Seq analysis (Figure 1) indicates that LINC00473 is but one of the top 100 or so most highly overexpressed genes when comparing FLC versus NML. What role do the other upregulated/downregulated genes have in the biology of FLC? Are there other hypotheses? Are these differentially expressed genes related to the same molecular pathways? This

appears to be partially addressed in Figure 4D, but it appears that there may be opportunities to better utilize the RNA-Seq cohort.

The investigation of other up- and downregulated genes in FLC is a major area of research in our lab and the field, and we appreciate the Reviewer's interest in this ongoing work. To address this question, we thought it would be most beneficial to assess the differentially expressed genes through gene set analysis. To complement this evaluation, we also provide more in-depth information about specific genes that have been studied in the context of FLC. However, this list of genes is not exhaustive due to the paucity of detailed mechanistic studies on this rare disease.

First, we used ENRICHR to perform gene enrichment analyses on all top upregulated (n=1666) and downregulated (n=1497) genes in FLC tumors versus non-malignant liver (NML). By focusing on the MSigDB and KEGG databases, we found the following pathway enrichment. The adjusted p-value is calculated using the Benjamini-Hochberg method for correction for multiple hypotheses testing. The gene lists followed the filtration criteria: base mean > 100; log2 fold change > 1 for upregulated genes or <-1 for downregulated genes; adjusted p-value <0.05.

Top Upregulated Genes

MSigDB:

Index	Name	P-value	Adjusted p-value	Odds Ratio	Combined score
1	Angiogenesis	4.813e-7	0.000004813	7.05	102.61
2	Epithelial Mesenchymal Transition	1.366e-30	6.832e-29	6.87	472.21
3	G2-M Checkpoint	1.372e-11	3.430e-10	3.55	88.76
4	Estrogen Response Late	1.692e-10	2.820e-9	3.35	75.42
5	E2F Targets	5.697e-10	7.121e-9	3.26	69.30
6	Hedgehog Signaling	0.008358	0.02612	3.15	15.09
7	Wnt-beta Catenin Signaling	0.006786	0.02423	3.01	15.04
8	UV Response Dn	0.00001910	0.00009549	2.68	29.17
9	Myogenesis	0.000001232	0.000008800	2.62	35.63
10	KRAS Signaling Up	0.000001232	0.000008800	2.62	35.63

KEGG:

Index	Name	P-value	Adjusted p-value	Odds Ratio	Combined score
1	ECM-receptor interaction	6.480e-9	0.000001925	4.67	88.09
2	Cell cycle	9.076e-7	0.0001348	3.25	45.18
3	Protein digestion and absorption	0.000002976	0.0002947	3.38	42.98
4	Pathways in cancer	0.000004183	0.0003106	1.85	22.89
5	Relaxin signaling pathway	0.00005535	0.003151	2.67	26.18
6	AGE-RAGE signaling pathway in diabetic complications	0.00006366	0.003151	2.95	28.50
7	Axon guidance	0.0001014	0.004301	2.28	21.00
8	Small cell lung cancer	0.0001761	0.006539	2.89	24.95
9	Cellular senescence	0.0002109	0.006960	2.32	19.68
10	Calcium signaling pathway	0.0004048	0.01202	1.96	15.33

Top Downregulated Genes

MSigDB:

Index	Name	P-value	Adjusted p-value	Odds Ratio	Combined score
1	Xenobiotic Metabolism	4.295e-47	2.148e-45	10.48	1119.05
2	Coagulation	1.442e-19	3.604e-18	6.56	284.73
3	Bile Acid Metabolism	1.078e-17	1.797e-16	7.03	274.77
4	TNF-alpha Signaling via NF-kB	1.827e-16	2.284e-15	4.59	166.21
5	Fatty Acid Metabolism	3.626e-16	3.626e-15	5.21	185.22
6	Cholesterol Homeostasis	1.177e-12	9.807e-12	7.22	198.26
7	Estrogen Response Late	6.201e-11	4.429e-10	3.56	83.77
8	Pperoxisome	7.252e-9	4.532e-8	4.40	82.44
9	Hypoxia	8.292e-8	4.146e-7	2.95	48.12
10	Estrogen Response Early	8.292e-8	4.146e-7	2.95	48.12

KEGG:

Index	Name	P-value	Adjusted p-value	Odds Ratio	Combined score
1	Complement and coagulation cascades	2.402e-27	7.157e-25	13.65	836.41
2	Drug metabolism	6.692e-26	9.971e-24	10.19	590.68
3	Metabolism of xenobiotics by cytochrome P450	4.338e-23	4.309e-21	12.67	652.16
4	Retinol metabolism	8.417e-21	6.271e-19	12.63	583.97
5	Glycine, serine and threonine metabolism	1.540e-17	9.180e-16	18.84	729.32
6	Bile secretion	5.212e-17	2.589e-15	8.04	301.29
7	Steroid hormone biosynthesis	4.009e-14	1.706e-12	9.33	287.91
8	Fatty acid degradation	4.167e-13	1.552e-11	11.96	340.96
9	Valine, leucine and isoleucine degradation	5.964e-13	1.975e-11	10.61	298.57
10	PPAR signaling pathway	1.177e-12	3.507e-11	7.22	198.26

Our key findings include:

- Multiple upregulated pathways are directly related to cancer, including Epithelial to Mesenchymal Transition, Angiogenesis, KRAS Signaling, and Pathways in Cancer.
- One of the most notable findings was the downregulation of Retinoic Acid Metabolism, a normal function of the liver. The suppression of this pathway and its potential therapeutic vulnerability in FLC is under direct investigation by our group in a separate project through the treatment of all-trans retinoic acid (ATRA, also known as tretinoin), an FDA-approved drug.
- Some of the top downregulated pathways include complement and coagulation cascades, drug metabolism, and cytochrome P450 metabolism, which are essential functions of the liver. It would be reasonable that these pathways are relatively low in activity in tumors compared to their very high levels in normal adjacent liver tissues.
- Other downregulated pathways represent basic liver functions, such as bile acid metabolism, bile acid secretion, and cholesterol metabolism. To maintain metabolic homeostasis, cholesterol is converted to bile acids to prevent the accumulation of xenobiotics, which are known to induce cellular damage and critical injury to the liver and other organs (J. Chiang, 2013, Compr Physiol). Interestingly, dysfunctional bile acid metabolism has been shown to play a role in hepatocellular carcinoma by inducing DNA damage and inflammation to increase tumor progression (Rezen, Rozman, et al., 2022, Cell Mol Life Sci; Cui et al., 2023, Sci Rep).

- Notably, several pathways are concordant with our LeGO-473ox vs. LeGO-Ctl engineered cell line RNA-seq analysis, including Glycolysis. This suggests that LINC00473 could play a crucial role in some of the major processes exploited by FLC tumors to facilitate cancer phenotypes.

Importantly, our Enrichr analyses on primary tumors and NML tissues have been incorporated into Supplementary Figure 1D and 1E. In the case the Reviewer may find it helpful, we have provided links to our ENRICHR analyses below.

Upregulated FLC vs. NML

- Link to Top Up All genes
  > https://maayanlab.cloud/Enrichr/enrich?dataset=aa10e0dd9093153ece2159b3d8090ab7

Downregulated FLC vs. NML

- Link to Top Down All genes
  > https://maayanlab.cloud/Enrichr/enrich?dataset=9e8ac776617a312218d2b4d2e54aa47e

Secondly, we curated specific genes from the top upregulated gene list that have been reported in the literature on FLC.

Gene	Description in FLC or Other Cancers	Reference
CA12, OAT, PAK3, PCSK1, CREB3L1	Part of a gene signature that distinguishes FLC from other liver cancers, including hepatocellular carcinoma and cholangiocarcinoma	Dinh et al., 2017, Sci Rep
CREB3L1	Predictive biomarker for the prognosis of multiple cancers and promotes tumor growth and metastasis in thyroid carcinoma	Lin et al., 2022, Front. Genet.; Pan et al., 2022, Mole Cancer
MUC14, MUC5B	Highly specific tumor-associated mucins in biliary tract cancer, as well as mucoepidermoid carcinoma which has been shown to be regulated by LINC00473	Handra-Luca et al., 2005, Am J Surg Pathol; Matull et al., 2008, Br J Cancer; Chen et al., 2018, Oncogene
VCAN	A major proteoglycan in FLC tumors that is produced in tumor epithelial cells and activated stellate cells. Known to promote metastasis through increased cellular adhesion and is associated with tumor progression in hepatocellular carcinoma.	Francisco et al., 2022, Cancer Res Commun; Zhangyuan et al., 2020, Oncogene; Tanaka et al., 2018, Int J Mol Sci
PRODH, PYCR1, OAT	Mitochondrial proteins shown to be strongly expressed in FLC tumors relative to NML through proteomic analysis and immunofluorescence validation	Levin et al., 2023, Sci Adv

One major objective in publishing this dataset is to foster collaboration and contribute to the advancement of the field of FLC research. Notably, this RNA-seq dataset comprises the largest number of FLC samples published to date. We believe that the RNA-seq analysis, combined with the provided resources and documentation, positions it as a valuable tool for researchers in FLC. We hope the

scientific community finds our dataset conducive to generating novel hypotheses and advancing our collective understanding of this rare and devastating cancer.

Several top upregulated genes are under active investigation by our group (SLC16A14) and others in the field. Importantly, our lab is currently harnessing this RNA-seq dataset in several multiomic analyses. One example is the integration of proteomics data on FLC vs NML to investigate post-transcriptional regulation in tumors and provide a comprehensive understanding of cellular events in FLC.

For this study, LINC00473 was a striking gene for investigation because of its implicated role in cAMP signaling across several contexts, including lung adenocarcinoma and human decidualization. This was intriguing to pursue in FLC because virtually all tumors display aberrant PKA signaling, a cAMP-dependent protein kinase.

Below we provide volcano plots of the differentially expressed genes between FLC vs. NML for Cohort 1 (our group) and Cohort 2 (from Dr. Taran Gujral's group, Fred Hutchinson Cancer Center).

Volcano Plot for Cohort 1:

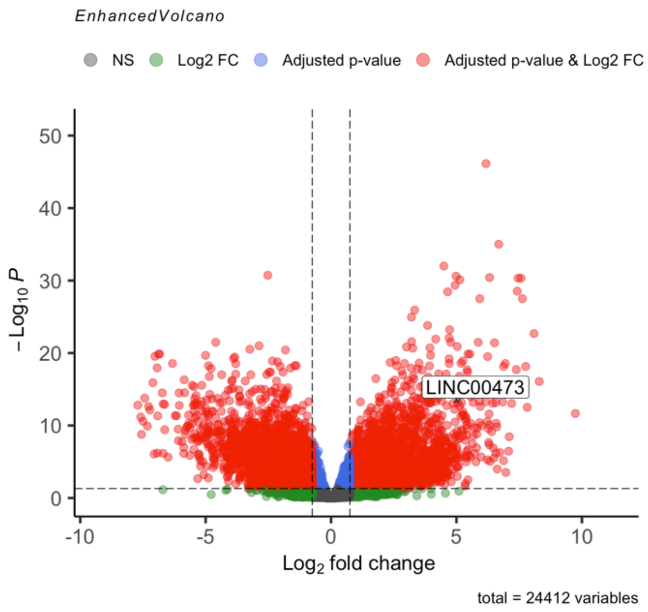

Volcano Plot for Cohort 2:

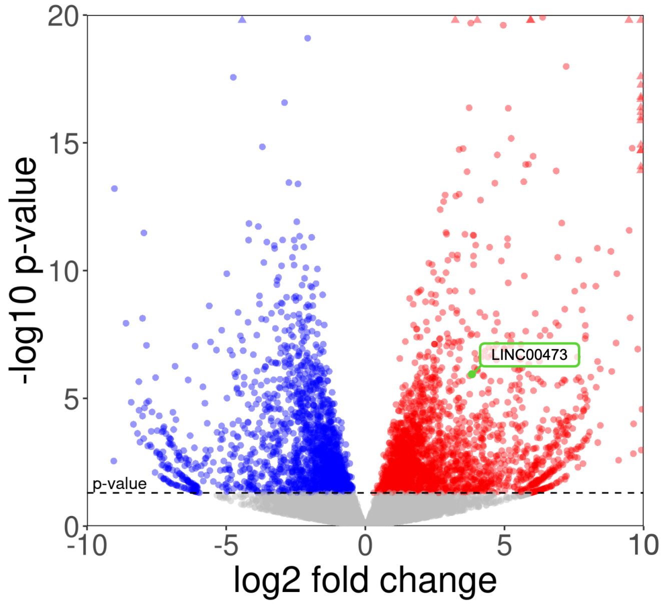

2. One thought would be to create gene sets based on the differentially expressed genes between FLC and NML. Those gene sets can be further analyzed for molecular pathways, etc. In addition, PCA or other dimensionality reduction analysis may show biologically relevant patterns in the data. It appears that the RNA-Seq data came from multiple batches. Has any effort been taken to assess for batch effects?

We thank the reviewer for their thoughtful comments. We sought to validate the results above (in response to Major Point #1) by implementing GSEA analysis on significantly up- and down-regulated genes. The gene sets are identical to those in the manuscript and in the Enrichr analysis above (filtration criteria: base mean > 100; log2 fold change > 1 for upregulated genes or <-1 for downregulated genes; adjusted p-value <0.05). Below, we summarize our findings.

Top upregulated pathways (n = 1667 genes):

	MSigDB PATHWAY	SIZE	ES	NES	NOM p-val	FDR q-val
1	HALLMARK_ESTROGEN_RESPONSE_LATE	46	0.68	2.14	0.000	0.024
2	HALLMARK_ESTROGEN_RESPONSE_EARLY	31	0.52	2.03	0.000	0.041
3	HALLMARK_ADIPOGENESIS	16	0.78	1.91	0.061	0.065
4	HALLMARK_APICAL_JUNCTION	36	0.45	1.87	0.009	0.063
5	HALLMARK_KRAS_SIGNALING_UP	39	0.63	1.79	0.049	0.082
6	HALLMARK_HYPOXIA	26	0.63	1.78	0.022	0.069
7	HALLMARK_EPITHELIAL_MESENCHYMAL_TRANSITION	75	0.53	1.6	0.145	0.176
8	HALLMARK_MYOGENESIS	38	0.4	1.6	0.045	0.161
9	HALLMARK_COMPLEMENT	16	0.64	1.57	0.075	0.167
10	HALLMARK_IL2_STAT5_SIGNALING	27	0.43	1.46	0.058	0.239

Notable pathways that are upregulated in FLC tumors relative to NML include those heavily implicated in cancer progression, such as KRAS signaling and Epithelial Mesenchymal Transition. Importantly, each of these pathways is also detected by Enrichr.

An enrichment plot of each pathway can be found below.

Top upregulated pathways

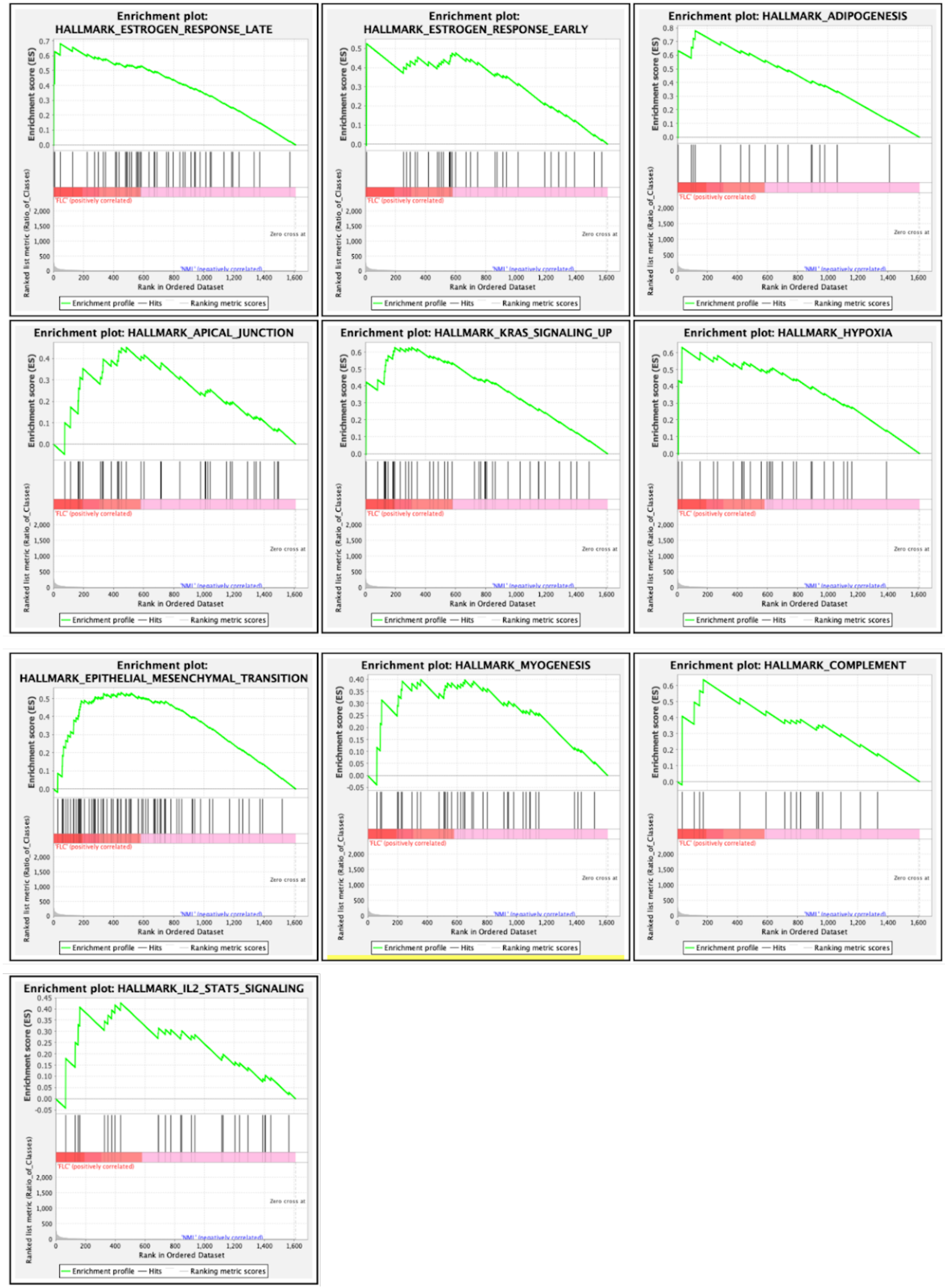

Top downregulated pathways (n = 1497 genes):

	MSigDB PATHWAY	SIZE	ES	NES	NOM p-val	FDR q-val
1	HALLMARK_ESTROGEN_RESPONSE_LATE	45	0.38	1.88	0.003	0.443
2	HALLMARK_IL6_JAK_STAT3_SIGNALING	19	0.68	1.82	0.018	0.311
3	HALLMARK_HYPOXIA	38	0.42	1.66	0.087	0.449
4	HALLMARK_ALLOGRAFT_REJECTION	15	0.64	1.63	0.055	0.396
5	HALLMARK_INTERFERON_GAMMA_RESPONSE	21	0.51	1.59	0.092	0.392
6	HALLMARK_ESTROGEN_RESPONSE_EARLY	40	0.34	1.56	0.042	0.361
7	HALLMARK_IL2_STAT5_SIGNALING	34	0.42	1.55	0.146	0.319
8	HALLMARK_INFLAMMATORY_RESPONSE	31	0.53	1.54	0.123	0.304
9	HALLMARK_HEME_METABOLISM	24	0.32	1.52	0.056	0.289
10	HALLMARK_UV_RESPONSE_DN	16	0.45	1.49	0.115	0.291

From both of our analyses using GSEA and Enrichr, we found that hypoxia, interferon/TNF-alpha signaling, and inflammation/PPAR pathways are activated in FLC tumors compared to NML. We note that both early and late estrogen response pathways were found to be simultaneously upregulated and suppressed in FLC tumors using both Enrichr and GSEA. This may be due to several genes within the estrogen response pathways that are associated with other cellular processes relevant to cancer, rather than directly linked to reproductive hormone biology.

An enrichment plot of each pathway can be found below.

Top downregulated pathways

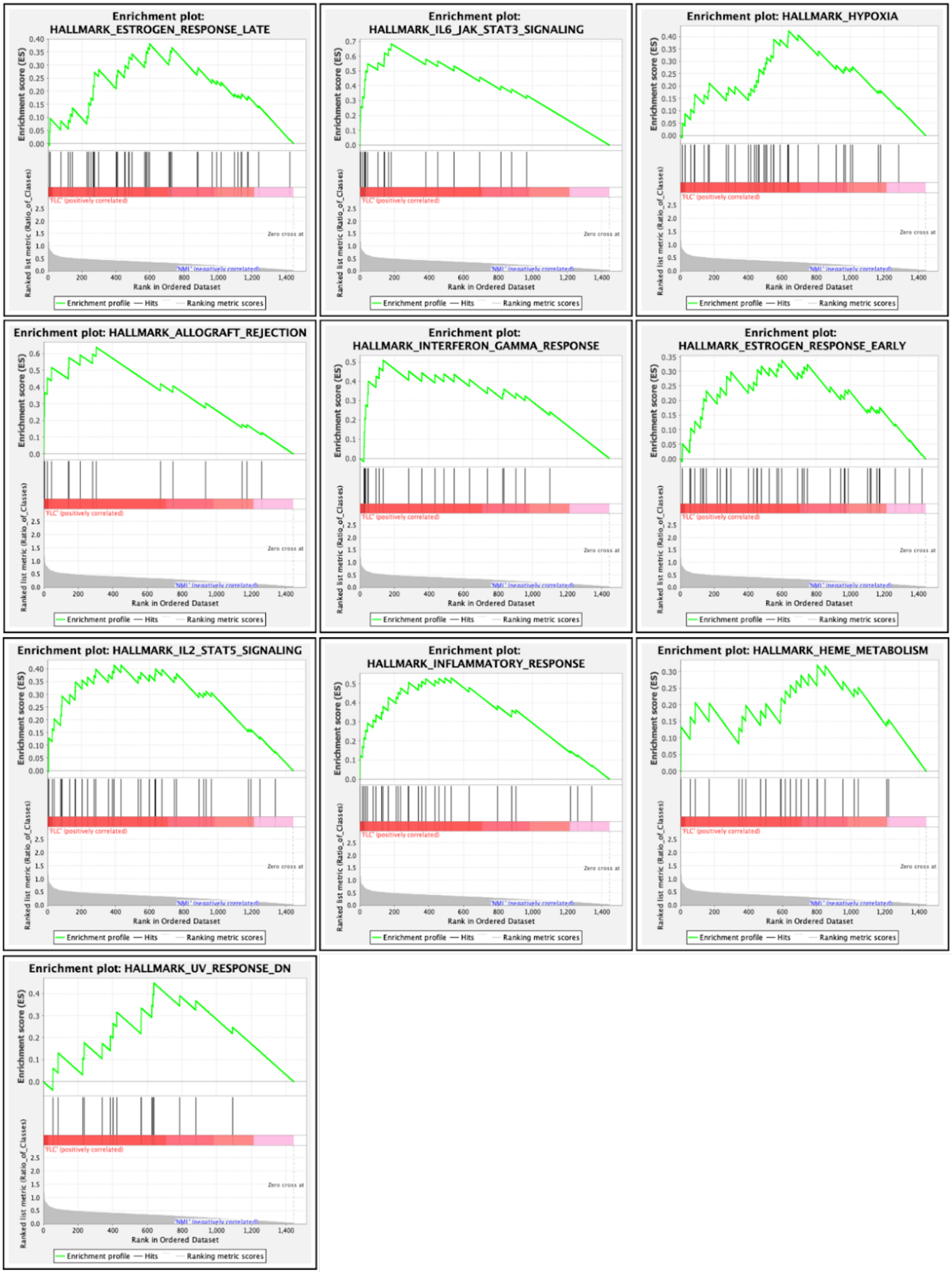

As per the Reviewer's request, we have provided PCA plots for both cohorts of primary patient samples below.

PCA for Cohort 1:

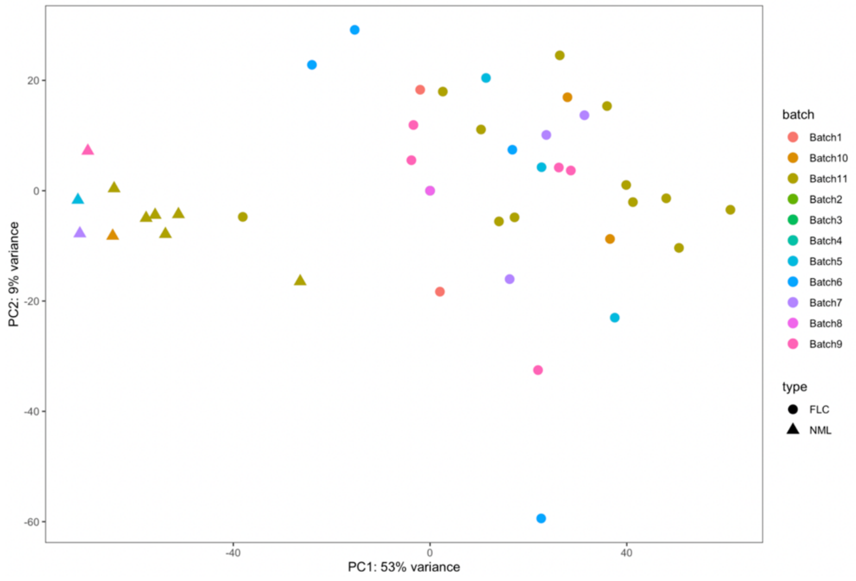

PCA for Cohort 2:

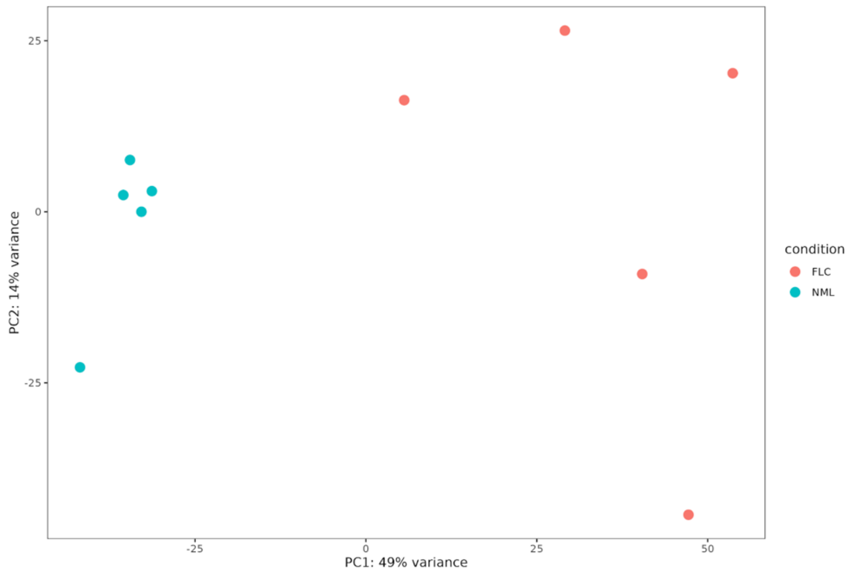

Regarding the query on the handling of batch effects in our RNA-seq pipeline, we would like to elaborate on our method. In our study, we employed the limma package, a widely recognized tool for the analysis of high-dimensional genomic data as part of our analysis. Specifically, we utilized the limma::removebatcheffect function that fits a linear model to the data, to batches of FLC or NML groups, and subsequently removes batch effects from expression data. This has been added to our methods section.

3. Figure 4E and F show different collections of gene sets. Figure 4E shows MSigDB and upregulated pathways. Figure 4F shows KEGG and downregulated pathways. The authors could show both the upregulated and downregulated pathways for a single collection of gene sets for consistency.

To provide consistency across the up- and down-regulated pathways, Figure 4F has been modified by replacing KEGG with MSigDB. Downregulated pathways denoted in KEGG have been moved to the supplementary figures.

4. In Figure 4E, “Uv response down” appears to be the top upregulated gene set but it is not discussed by the authors. What is the interpretation of this result?

A deeper examination of the specific genes in the “UV response down” pathway revealed that several are oncogenes, proto-oncogenes, or implicated in promoting cancer cell phenotypes in multiple cancers. These include MYC, ERBB2 (HER2), SNAI2, and KIT, as well as NRP1, LPAR1, and ABCC1 (**bolded in the table below**). Our assessment indicates that these genes are strongly linked to cancer and pro-growth pathways, and only partially overlap with those involved in the UV response.

We provide a detailed list of the upregulated genes enriched in the “UV Response Down” pathway below.

**Gene**	**Function**	**Reference**
PHF3	PHD finger protein 3, is involved in chromatin remodeling and transcription regulation.	https://www.ncbi.nlm.nih.gov/gene/23469
**NRP1**	Neuropilin-1 participates in axon guidance and angiogenesis. Co-receptor that enhances growth factors including VEGF and hepatocyte growth factor to promote tumor initiation, growth, and metastasis	https://pubmed.ncbi.nlm.nih.gov/22948112/; https://pubmed.ncbi.nlm.nih.gov/22025255/; https://pubmed.ncbi.nlm.nih.gov/32675311/;
CITED2	Cbp/p300-interacting transactivator, involved in transcriptional regulation. Reported to promote cancer phenotypes in multiple cancers to enhance tumor cell survival, maintain cancer stem cells, and modulate hypoxic response.	https://www.ncbi.nlm.nih.gov/pmc/articles/PMC7439111/; https://www.ncbi.nlm.nih.gov/pmc/articles/PMC7360518/
SDC2	Syndecan-2 is a cell surface proteoglycan implicated in cell adhesion and signaling. Implicated as a diagnostic biomarker of colorectal cancer.	https://pubmed.ncbi.nlm.nih.gov/31707342/; https://pubmed.ncbi.nlm.nih.gov/35436855/; https://pubmed.ncbi.nlm.nih.gov/37193183/
GCNT1	Beta-1,6-N-acetylglucosaminyltransferase catalyzes glycosylation reactions. Regulates tumorigenicity and promotes tumor cell invasion in breast and prostate cancer.	https://pubmed.ncbi.nlm.nih.gov/22454501/; https://pubmed.ncbi.nlm.nih.gov/24854630/; https://pubmed.ncbi.nlm.nih.gov/37813880/
**LPAR1**	Lysophosphatidic acid receptor 1, a G-protein-coupled receptor, is involved in signaling pathways promoting cell growth. Stimulates cancer phenotypes through PI3K/AKT signaling and crosstalk with EGFR.	https://www.ncbi.nlm.nih.gov/pmc/articles/PMC8394178/; https://www.ncbi.nlm.nih.gov/pmc/articles/PMC8306951/; https://pubmed.ncbi.nlm.nih.gov/31384176/; https://pubmed.ncbi.nlm.nih.gov/24212818/

PIK3R3	Phosphoinositide 3-kinase regulatory subunit 3, part of the PI3K signaling pathway. Overexpressed in multiple cancers.	https://pubmed.ncbi.nlm.nih.gov/22876838/; https://pubmed.ncbi.nlm.nih.gov/17875760/; https://www.ncbi.nlm.nih.gov/pmc/articles/PMC5790378/#cit0006
VLDLR	Very low-density lipoprotein receptor, involved in lipid metabolism and signaling. Elevated expression is associated with aggressive cancers and metastasis phenotypes.	https://pubmed.ncbi.nlm.nih.gov/34429409/; https://pubmed.ncbi.nlm.nih.gov/21047397/; https://pubmed.ncbi.nlm.nih.gov/27199348/
LAMC1	Laminin subunit gamma-1, a component of the extracellular matrix. Predicts poor prognosis in hepatocellular carcinoma and endometrial cancer. Enhances tumor cell invasion and migration. Upregulated by TFG-beta via NFkB-CXCL1-STAT3 signaling.	https://pubmed.ncbi.nlm.nih.gov/31912669/; https://pubmed.ncbi.nlm.nih.gov/28928891/; https://pubmed.ncbi.nlm.nih.gov/34218518/
LTBP1	Latent-transforming growth factor beta-binding protein 1 is involved in TGF-beta signaling. Promotes cancer phenotypes including epithelial-mesenchymal-transition and cancer-associated fibroblast transformation	https://pubmed.ncbi.nlm.nih.gov/34418460/; https://pubmed.ncbi.nlm.nih.gov/32216815/
**IGF1R**	Insulin-like growth factor 1 receptor, part of the IGF signaling pathway. Promotes tumorigenesis and confers chemoresistance in multiple cancers.	https://pubmed.ncbi.nlm.nih.gov/32493414/; https://pubmed.ncbi.nlm.nih.gov/36017326/; https://www.ncbi.nlm.nih.gov/pmc/articles/PMC10066341/
GJA1	Gap junction alpha-1 protein encoding connexin 43, involved in cell communication. Predictor of poor patient prognosis in stomach cancer and colorectal cancer.	https://www.ncbi.nlm.nih.gov/pmc/articles/PMC7678473/; https://pubmed.ncbi.nlm.nih.gov/30814684/
EFEMP1	EGF-containing fibulin-like extracellular matrix protein 1, implicated in cell adhesion. Implicated to promote cancer phenotypes in ovarian cancer via the AKT pathway. Also found to suppress glioma cell growth.	https://www.ncbi.nlm.nih.gov/pmc/articles/PMC5216990/; https://pubmed.ncbi.nlm.nih.gov/21955618/
PRKAR2B	Protein kinase cAMP-dependent type II regulatory subunit beta, involved in cAMP signaling. Implicated in tumor growth in prostate cancer by promoting glycolysis via HIF1-alpha. Promotes metastasis of prostate cancer via Wnt/Beta-catenin signaling and EMT transition	https://pubmed.ncbi.nlm.nih.gov/33025691/; https://pubmed.ncbi.nlm.nih.gov/29761841/
**MYC**	Myelocytomatosis oncogene is a well-known proto-oncogene involved in cell cycle regulation.	https://pubmed.ncbi.nlm.nih.gov/10378693/; https://pubmed.ncbi.nlm.nih.gov/22464321/

**ERBB2**	Erb-B2 receptor tyrosine kinase 2, a receptor for growth factors. Commonly referred to as HER2, often amplified in cancers.	https://pubmed.ncbi.nlm.nih.gov/25276427/; https://pubmed.ncbi.nlm.nih.gov/17471238/
CDON	Cell Adhesion Associated, Oncogene Regulated cell surface receptor. Member of the immunoglobulin superfamily. Mediates cell-cell interactions and positively regulates myogenesis.	https://www.ncbi.nlm.nih.gov/gene/50937
**ABCC1**	ATP-binding cassette sub-family C member 1, involved in drug resistance in cancer cells.	https://pubmed.ncbi.nlm.nih.gov/24670052/; https://pubmed.ncbi.nlm.nih.gov/18085475/; https://pubmed.ncbi.nlm.nih.gov/25281745/
IGFBP5	Insulin-like growth factor-binding protein 5, regulates IGF signaling. Promotes cancer invasion via ROR1/HER2-CREB signaling. Plays diverse roles in cancer progression as an oncogene in prostate, breast, and pancreatic cancers via IGF-dependent or IGF-independent signaling pathways. Also implicated as a tumor suppressor in melanoma, cervical carcinoma, and ovarian cancer.	https://pubmed.ncbi.nlm.nih.gov/36949068/; https://pubmed.ncbi.nlm.nih.gov/36465383/;
ATRX	ATRX chromatin remodeler that regulates DNA damage response and prevents chromosomal instability via homologous recombination. Among the most frequently mutated genes in human cancers.	https://pubmed.ncbi.nlm.nih.gov/36894374/; https://pubmed.ncbi.nlm.nih.gov/28062559/
ATP2B4	Plasma membrane calcium-transporting ATPase 4, regulates cellular calcium levels.	https://www.ncbi.nlm.nih.gov/gene/493
ATP2B1	Plasma membrane calcium-transporting ATPase 1, regulates cellular calcium levels.	https://www.ncbi.nlm.nih.gov/gene/490
DAB2	DAB adaptor protein 2, a mitogen-responsive phosphoprotein involved in clathrin-mediated endocytosis and signal transduction. Regulator of anti- and pro-tumorigenic pathways	https://pubmed.ncbi.nlm.nih.gov/36614139/
ARHGEF9	Rho guanine nucleotide exchange factor 9, regulates Rho GTPases in cellular processes. Biomarker and regulator of cancer phenotypes in hepatocellular carcinoma	https://pubmed.ncbi.nlm.nih.gov/31283007/; https://pubmed.ncbi.nlm.nih.gov/20335658/
COL3A1	Collagen type III alpha 1 chain, a component of connective tissues. Potential prognostic biomarker in ovarian carcinoma and esophageal squamous cell carcinoma	https://pubmed.ncbi.nlm.nih.gov/31533654/; https://pubmed.ncbi.nlm.nih.gov/35598375/

NFIB	Nuclear factor I/B is a transcription factor involved in cell differentiation. A driver of cancer progression and metastasis in lung adenocarcinoma, and skin and breast cancers	https://pubmed.ncbi.nlm.nih.gov/27374332/; https://pubmed.ncbi.nlm.nih.gov/27373156/; https://pubmed.ncbi.nlm.nih.gov/28596133/
MAP1B	Microtubule-associated protein 1B, regulates microtubule dynamics. Implicated as a predictor of poor prognosis in urothelial carcinoma and plays a role in mediating mTOR-targeted drug resistance	https://pubmed.ncbi.nlm.nih.gov/32182788/; https://pubmed.ncbi.nlm.nih.gov/29136244/
**KIT**	Tyrosine-protein kinase KIT is involved in cell signaling and critical proto-oncogenic regulator of cell proliferation, survival, and migration	https://pubmed.ncbi.nlm.nih.gov/35490363/; https://pubmed.ncbi.nlm.nih.gov/27536065/; https://pubmed.ncbi.nlm.nih.gov/9338533/
**SNAI2**	Snail homolog 2, a zinc-finger transcription factor that sustains cancer stem cell ability and EMT transition in multiple indications	https://pubmed.ncbi.nlm.nih.gov/35768509/; https://pubmed.ncbi.nlm.nih.gov/33023950/; https://pubmed.ncbi.nlm.nih.gov/17550342/
PLPP3	Phospholipid phosphatase 3, regulates lipid signaling pathways.	https://www.ncbi.nlm.nih.gov/gene/8613

5. In Figure 4F, Cell Cycle appears to be downregulated, however the authors showed that LINC00473 promotes cell proliferation. How do the authors reconcile this contradictory result?

We examined the specific downregulated genes in the “Cell Cycle” pathway and found that multiple genes are related to laminin or type 4 collagen, such as LAMA/B/C, COL4A1/5/6, and ITGA2. Interestingly, we noted the suppression of both XIAP and BAX, which are anti- and pro-apoptotic factors, respectively. This suggests to us that compensatory gene expression programs may be activated in response to LINC00473 induction and subsequent suppression of apoptosis in FLC cells. We also note that positive regulators of cell cycle progression, specifically PIK3R3, CDK6, and CCND1, were decreased in the context of LINC00473 overexpression. This is consistent with our findings that LINC00473 promotes cell proliferation in large part due to decreased levels of apoptosis (Figure 4H), and not necessarily through elevated cell cycle progression (Figure 4G).

We provide a detailed list of the downregulated genes enriched in the “Cell Cycle” pathway.

**Genes**	**Function**	**Reference**
LAMA1, LAMB2, LAMA4, LAMB1, LAMC1	Laminin subunits alpha, beta, and gamma	https://www.ncbi.nlm.nih.gov/gene/284217; https://www.ncbi.nlm.nih.gov/gene/3913; https://www.ncbi.nlm.nih.gov/gene/3910; https://www.ncbi.nlm.nih.gov/gene/3912; https://www.ncbi.nlm.nih.gov/gene/3915
COL4A1, COL4A6, COL4A5	Type IV collagen, the main component of the basement membrane	https://www.ncbi.nlm.nih.gov/gene/1282; https://www.ncbi.nlm.nih.gov/gene/1288; https://www.ncbi.nlm.nih.gov/gene/1287; www.pubmed.ncbi.nlm.nih.gov/21157337/

ITGA2	Integrin subunit alpha 2 receptor that binds to multiple ligands, including collagen and laminin	https://www.ncbi.nlm.nih.gov/gene/3673
PIK3R3	Phosphoinositide 3-kinase regulatory subunit 3, part of the PI3K signaling pathway. Promotes cell proliferation through the p53/p31 signaling pathway	https://pubmed.ncbi.nlm.nih.gov/32973127/
XIAP	X-linked inhibitor of apoptosis. Plays a crucial role in inhibiting cell death through the inhibition of caspases.	https://pubmed.ncbi.nlm.nih.gov/25120954/
CDK6	Cyclin-Dependent Kinase 6, a positive regulator of cell cycle progression. Forms complexes with cyclins to promote the transition of G1 to S phase.	https://www.ncbi.nlm.nih.gov/gene/1021
CCND1	Cyclin D1 is a positive regulator of cell cycle progression that forms complexes with CDK4 and CDK6 to promote G1 to S transition.	https://www.ncbi.nlm.nih.gov/gene/595
BAX	Bcl-2 Associated Protein, a pro-apoptotic protein. Reduced BAX expression is associated with oral cancer development and poor prognosis of non-small cell lung cancer and colorectal cancer patients.	https://pubmed.ncbi.nlm.nih.gov/28233171/; https://pubmed.ncbi.nlm.nih.gov/18772168/; https://pubmed.ncbi.nlm.nih.gov/24122668/;

6. In Figures 4E and F, it appears that the color of the bars and the height of the bars in the bar plot are both using the same information. For GSEA results, it is typical to use the Normalized Enrichment Score (NES) or Enrichment Score (ES) in some fashion because p-value statistics are heavily dependent on gene set size.

We are grateful to the Reviewer for their valuable comments. In line with the Reviewer's suggestion, we made the following modifications in Figures 4E and 4F, as well as other pathway enrichment analyses in the supplement and new data in Figure 6, to provide clearer insight into our findings.

- Replaced the p-value on the x-axis with the Odds Ratio from our Enrichr analysis. The Odds Ratio can be used as a reliable substitute for NES or ES.
- Changed the color scheme to orange for significantly (p value < 0.05) upregulated pathways and blue for suppressed pathways, such that color intensity represents the odds ratio.

To provide validation of the results from the Enrichr analysis, we performed GSEA on the up- and down-regulated gene sets and outline the pathways of interest below.

Top Upregulated Pathways (n = 1403 genes)

	MSigDB PATHWAY	SIZE	ES	NES	NOM p-val	FDR q-val
1	HALLMARK_EPITHELIAL_MESENCHYMAL_TRANSITION	33	0.39	0.85	0.679	1
2	HALLMARK_ESTROGEN_RESPONSE_EARLY	25	0.26	0.82	0.679	1
3	HALLMARK_ADIPOGENESIS	29	0.18	0.81	0.679	1
4	HALLMARK_GLYCOLYSIS	35	0.23	0.79	0.679	1
5	HALLMARK_HYPOXIA	26	0.17	0.77	0.679	1
6	HALLMARK_UV_RESPONSE_DN	30	0.31	0.64	0.679	1
7	HALLMARK_MTORC1_SIGNALING	31	0.1	0.48	0.679	1

Top Downregulated Pathways (n = 1374 genes)

	MSigDB PATHWAY	SIZE	ES	NES	NOM p-val	FDR q-val
1	HALLMARK_P53_PATHWAY	34	0.48	1.53	0	0.177
2	HALLMARK_INFLAMMATORY_RESPONSE	15	0.52	1.51	0	0.177
3	HALLMARK_HYPOXIA	26	0.5	1.48	0	0.177
4	HALLMARK_ADIPOGENESIS	17	0.39	1.43	0	0.177
5	HALLMARK_APOPTOSIS	25	0.49	1.43	0	0.177
6	HALLMARK_MYOGENESIS	25	0.48	1.37	0	0.177
7	HALLMARK_PI3K_AKT_MTOR_SIGNALING	11	0.35	1.31	0	0.177
8	HALLMARK_GLYCOLYSIS	12	0.4	1.3	0	0.262
9	HALLMARK_COAGULATION	10	0.57	1.3	0	0.253
10	HALLMARK_TNFA_SIGNALING_VIA_NFKB	44	0.53	1.29	0	0.271
11	HALLMARK_MTORC1_SIGNALING	20	0.35	1.24	0	0.317
12	HALLMARK_XENOBIOTIC_METABOLISM	12	0.47	1.22	0	0.384
13	HALLMARK_INTERFERON_GAMMA_RESPONSE	13	0.39	1.17	0	0.463
14	HALLMARK_EPITHELIAL_MESENCHYMAL_TRANSITION	20	0.47	1.14	0	0.486
15	HALLMARK_CHOLESTEROL_HOMEOSTASIS	12	0.34	1.11	0	0.545

By performing GSEA on the significantly differentially expressed genes in LeGO-473ox cells vs. LeGO-Ctl, we found strong concordance of activated and suppressed pathways with our previous Enrichr analysis (Figures 4E and 4F).

Notably, our GSEA analysis found that pathways related to epithelial mesenchymal transition, glycolysis, UV response down, hypoxia, and adipogenesis were upregulated in response to LINC00473 overexpression in FLC cells compared to empty vector control (Figure 4E). Additionally, the top suppressed pathways detected by GSEA include the p53 pathway, apoptosis, and TNF-alpha signaling via NF-kB (Figure 4F).

We note that the normalized enrichment scores and nominal p-values for this GSEA enrichment analysis are not particularly striking. This may be due to the lower number of input genes in these gene sets. Further, we note that glycolysis appears to be suppressed, which was not expected given our previous analyses and evidence from Seahorse metabolic assays. To dissect this further, we inspected the individual genes and found that only 1 of 12 suppressed genes are directly associated with glycolysis. The majority of the other 11 are associated with cell cycle regulation and glycosaminoglycan biosynthesis. A table of the genes listed in the downregulated “glycolysis” pathway is provided below:

Gene	Description	Function
CDK1	Cyclin-dependent kinase 1	Cell cycle regulation
HMMR	Hyaluronan-mediated motility receptor	Cell adhesion and motility
PPP2CB	Protein phosphatase 2 catalytic subunit beta	Protein dephosphorylation
IRS2	Insulin receptor substrate 2	Insulin signaling and glucose metabolism
SAP30	Sin3A-associated protein, 30 kDa	Transcriptional regulation
AURKA	Aurora kinase A	Cell cycle regulation
LDHC	Lactate dehydrogenase C	Lactate metabolism
ENO2	Enolase 2	Glycolysis
B3GAT1	Beta-1,3-glucuronyltransferase 1	Glycosaminoglycan biosynthesis
CHST1	Carbohydrate sulfotransferase 1	Glycosaminoglycan biosynthesis
ISG20	Interferon-stimulated exonuclease gene 20	RNA degradation and antiviral response
GAL3ST1	Galactose-3-O-sulfotransferase 1	Glycosaminoglycan biosynthesis

7. Figure 4I and 4J: instead of assessing the significance of gene set overlaps and showing Venn diagrams as in Figure 4I, it may be more useful to create gene sets and perform GSEA. I would recommend creating gene sets of different sizes from the RNA-Seq analysis of FLC vs NML (top 25, 50, 100, 200, 500 genes) and then run GSEA using the ranked list of LeGO-473ox relative to control genes as an input. An overlap of 188 genes might have some statistical significance, but 188 out of 1403 or 1667 genes suggests that there are many other differentially expressed genes that cannot be attributed to LINC00473 biology.

We thank the reviewer for outlining an alternative approach to strengthen our findings. As recommended by the reviewer, we created gene sets of the Top 25, 50, 100, 200, and 500 genes from the RNA-seq analysis on FLC vs. NML, and subsequently performed GSEA on a ranked list of upregulated genes in LeGO-473ox vs. LeGO-Ctl. This analysis yielded normalized enrichment scores that range from 1.01 (Top 25 gene set) to >1.18 (Top 200 gene set).

Generally, recurrent overlapping upregulated genes across most gene sets include CPE, LPCAT1, PRODH, and SLC7A11 (Figure 4I), which are concordant with our approach that leverages hypergeometric testing. Additionally, we found an intersection of key FLC marker genes, including CA12, VCAN, RPS6KA2, BACE2, SLC16A14, and NOVA1 (Figure 4D). However, we note that only the Top 200 gene set analysis yielded a significant nominal p-value, and for this reason, we provide details on this particular gene list below. Note that only the top 50 (of 200) genes are shown in the interest of space.

	Top 500	Top 200	Top 100	Top 50	Top 25
Enrichment Score (ES)	0.6313	0.6563	0.6043	0.7116	0.6208
Normalized Enrichment Score (NES)	1.1374	1.1847	1.0783	1.1522	1.0137
Nominal p-value	0.1700	0.0000	0.1700	0.1700	0.5300
FWER p-Value	0.3600	0.1700	0.3600	0.3600	0.7200

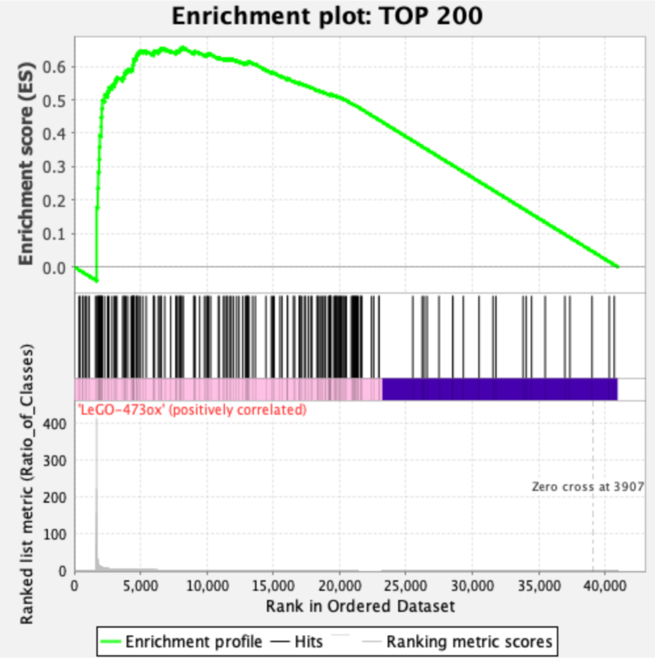

RANK	SYMBOL	RANK IN GENE LIST	RANK METRIC SCORE	CORE ENRICHMENT
1	TCF23	373	Infinity	Yes
2	CAPN14	450	Infinity	Yes
3	CIDEA	645	Infinity	Yes
4	ARPP21	681	Infinity	Yes
5	KCNJ6	851	Infinity	Yes
6	ACKR1	971	Infinity	Yes
7	EGFL6	1143	Infinity	Yes
8	SPP1	1610	Infinity	Yes
9	KCNQ3	1699	78.034	Yes
10	MUC5B	1786	21.058	Yes
11	VCAN	1823	17.31	Yes
12	GPC3	1883	14.154	Yes
13	HOXD9	1900	13.434	Yes
14	TENM4	1932	12.527	Yes
15	SLC22A31	2043	10.255	Yes
16	SLC7A11	2048	10.21	Yes
17	SYNDIG1	2090	9.362	Yes
18	UNC5D	2123	9.006	Yes

19	NQO1	2292	7.025	Yes
20	EBF2	2523	5.639	Yes
21	CDH17	2616	5.281	Yes
22	CA12	2853	4.544	Yes
23	LPAR3	3031	4.026	Yes
24	SPINK5	3121	3.91	Yes
25	LSAMP	3232	3.737	Yes
26	CPE	3657	3.186	Yes
27	PRKG2	3766	3.083	Yes
28	PRODH	3783	3.064	Yes
29	DYDC2	3897	2.963	Yes
30	NOVA1	4043	2.848	Yes
31	REG1A	4292	2.661	Yes
32	FRRS1L	4425	2.58	Yes
33	OTX1	4429	2.579	Yes
34	MRAP2	4459	2.557	Yes
35	OLFML2B	4473	2.551	Yes
36	DIO2	4510	2.524	Yes
37	GULP1	4665	2.443	Yes
38	ECEL1	4691	2.43	Yes
39	CNTN1	4751	2.402	Yes
40	GXYLT2	4853	2.348	Yes
41	TESC	4967	2.301	Yes
42	GALNTL6	5164	2.221	Yes
43	PLAC4	5429	2.108	Yes
44	HOPX	5972	1.918	Yes
45	TCN1	5990	1.913	Yes
46	IRF4	6041	1.901	Yes
47	CDH11	6342	1.817	Yes
48	SLC6A11	6427	1.799	Yes
49	HAVCR1	6536	1.768	Yes
50	THBS4	6566	1.76	Yes

We agree with the Reviewer that many genes may be differently regulated by LINC00473 depending on cellular context. While our evidence suggests that this lncRNA plays an important role in cancer phenotypes, we do not believe that it is the only effector of DNAJB1-PRKACA, and therefore not responsible for all or even most of the vast gene expression changes in FLC tumors. Specifically, it would be reasonable that LINC00473 functions through alternative mechanisms that hinge on the cellular and molecular contexts within primary tumors, which may be absent in FLC cells in culture. Nonetheless, it remains intriguing to us that the overexpression of a single lncRNA leads to the upregulation of nearly 200 genes that are aberrantly elevated in primary FLC tumors.

8. In Figure 4J, the top downregulated gene set is TNFa-NFKB signaling, but this is not discussed. What is the interpretation of this finding?

By assessing the individual genes that were downregulated in the "TNFa-NFKB" pathway, we found that multiple genes are tumor suppressor genes or have suggested anti-cancer functions. These include KLF6/10, EGR1, and GADD45A/B. It is interesting to note that certain genes have been identified to act as both promoters and inhibitors of tumor growth, depending on individual cancer contexts, including JUN, FOS, FOSB, NFKBIE, and ATF3.

This is consistent with the known duality of TNFa signaling in its paradoxical role in promoting and inhibiting cancer progression (https://pubmed.ncbi.nlm.nih.gov/31417576/, https://pubmed.ncbi.nlm.nih.gov/20712570/, https://pubmed.ncbi.nlm.nih.gov/31900901/).

We provide a detailed list of the downregulated genes enriched in the "TNFa-NFKB" pathway below.

Gene	Function Description	References
KLF10	Kruppel-like factor 10, transcriptional regulator. Tumor suppressor in multiple cancers	https://pubmed.ncbi.nlm.nih.gov/29799499/; https://pubmed.ncbi.nlm.nih.gov/34702956/; https://pubmed.ncbi.nlm.nih.gov/23994618/
EGR1	Early growth response 1, transcription factor. Exhibits tumor suppressor functions by monitoring DNA damage and promoting tumor cell apoptosis.	https://pubmed.ncbi.nlm.nih.gov/33842351/; https://pubmed.ncbi.nlm.nih.gov/34488929/; https://pubmed.ncbi.nlm.nih.gov/31748910/
JUN	Transcription factor Jun forms the AP-1 early response transcription factor in combination with Fos. Initially discovered as a proto-oncogene, recent studies found that AP-1 transcribes both positive and negative regulators of cell cycle progression, like CyclinA or p19(ARF).	https://pubmed.ncbi.nlm.nih.gov/20060892/; https://pubmed.ncbi.nlm.nih.gov/16676006/
GADD45B	Growth arrest and DNA damage-inducible protein beta. Exhibits tumor suppressive properties by promoting apoptosis	https://pubmed.ncbi.nlm.nih.gov/22515981/; https://pubmed.ncbi.nlm.nih.gov/31296259/; https://pubmed.ncbi.nlm.nih.gov/29716964/
GADD45A	Growth arrest and DNA damage-inducible protein alpha. Exhibits tumor suppressive properties by promoting apoptosis	https://pubmed.ncbi.nlm.nih.gov/22515981/
FOS	Transcription factor c-Fos forms the AP-1 early response transcription factor in combination with c-Jun. Initially discovered as a proto-oncogene, recent studies found that AP-1 transcribes both positive and negative regulators of cell cycle progression, like CyclinA or p19(ARF).	https://pubmed.ncbi.nlm.nih.gov/20060892/; https://pubmed.ncbi.nlm.nih.gov/16676006/
RCAN1	Regulator of calcineurin 1, involved in signaling. Repression of RCAN1 mediates cell proliferation and cell metabolism in prostate cancer	https://pubmed.ncbi.nlm.nih.gov/35067783/

KLF6	Kruppel-like factor 6, transcriptional regulator. A known tumor suppressor.	https://pubmed.ncbi.nlm.nih.gov/11752579/; https://pubmed.ncbi.nlm.nih.gov/15057748/; https://pubmed.ncbi.nlm.nih.gov/15486921/
NFIL3	Nuclear factor interleukin-3-regulated protein.	https://www.ncbi.nlm.nih.gov/gene/4783
MAFF	MAF basic leucine zipper transcription factor that binds to US-2 elements in the promoter of the oxytocin receptor (OTR) gene	https://www.ncbi.nlm.nih.gov/gene/23764
FOSB	Proto-oncogene c-FosB, transcription factor. Mediator of apoptosis.	https://pubmed.ncbi.nlm.nih.gov/36311014/; https://pubmed.ncbi.nlm.nih.gov/12618758/
NFKBIE	NF-kappa-B inhibitor epsilon, a negative regulator of NF-kappa-B. NF-kappa-B plays contrasting roles in cancer as a pro- and anti-tumorigenic regulator	https://pubmed.ncbi.nlm.nih.gov/32958760/; https://pubmed.ncbi.nlm.nih.gov/15102437/; https://pubmed.ncbi.nlm.nih.gov/29315242/
IER2	Immediate early response 2, predicted to enable DNA binding activity	https://www.ncbi.nlm.nih.gov/gene/9592
ATF3	Activating transcription factor 3. Mediates dual roles in promoting or suppressing cancer cell phenotypes across different indications	https://pubmed.ncbi.nlm.nih.gov/35685455/; https://pubmed.ncbi.nlm.nih.gov/17952119/
MCL1	Myeloid cell leukemia 1, an anti-apoptotic protein. Negative regulator of tumor suppressor p73 as part of Bcl-2 signaling. Expression is known to oscillate at different stages of the cell cycle.	https://pubmed.ncbi.nlm.nih.gov/33144577/; https://pubmed.ncbi.nlm.nih.gov/34475520/

Reviewer #2:

Fibrolamellar carcinoma is a rare cancer with limited options for patients due to late diagnosis and limited success on therapeutic strategies targeting the common DP-fusion established to be a driver of FLC. Therefore looking for downstream targets that could be developed is a very worthwhile effort. The establishment of LINC00473 as among the most highly upregulated genes in FLC (with limited number of samples albeit) and the subsequent work to carefully compare and dissect the expression LINC00473 from its host protein coding gene PDE10A is an important contribution. The demonstration in PDX models that patient-derived models of FLC, notably patient-derived xenograft (PDX) tissue as well as an FLC cell line established from this PDX, and found that DP fusion and LINC00473are concordantly elevated and siRNA studies to establish that Silencing of the DP fusion leads to LINC00473 suppression and siRNAs to LINC00473 impacts apoptosis, glycolysis and mitochondrial activity provides evidence for developing therapeutic strategies to impact the metabolic pathways driving FLC. In summary this is a well conducted study and more importantly provides evidence for role for LncRNAs as potential targets for cancer treatment.

We are grateful to the Reviewer for taking the time and effort to thoroughly examine and consider our work. We appreciate their thoughtful comments.

Reviewer #3:
The manuscript "DNAJB1-PRKACA fusion protein-regulated LINC00473 promotes tumor growth and alters mitochondrial fitness in fibrolamellar carcinoma" by Ma et al. utilizes functional genomics and in vivo models to characterize the role of the lncRNA LINC00473 in fibrolamellar carcinoma.

Major points:
1. It does not appear from Figure 1A that LINC00473 is the most differentially expressed gene in the bulk RNA-sequencing analyses. Is there some interest in these other genes and what they might do?

We appreciate the Reviewer's interest in this ongoing work on other differentially expressed genes and share their enthusiasm for advancing understanding in this area. This study focused on investigating LINC00473, which has been implicated in cAMP signaling in various contexts, including lung adenocarcinoma and human decidualization. Specifically, one report found that LINC00473 is a cAMP/CREB target gene necessary for the growth and survival of non-small cell lung tumors harboring an inactivating mutation in the LKB1 tumor suppressor gene. Other studies demonstrated that LINC00473 is downstream of the fusion oncoprotein CRTC1-MAML2 in mucoepidermoid carcinoma (MEC) and that the deletion of CRTC2/3 leads to decreased levels of LINC00473. This is especially interesting because MUC5B and MUC14 are tumor-specific mucins in MEC and biliary tract carcinoma, and are among the most highly expressed genes in FLC tumors. Furthermore, our group previously reported that the LINC00473 locus is associated with an FLC tumor-specific super enhancer that is enriched for CREB-binding motifs. Together, this limited but intriguing knowledge of LINC00473 compelled us to pursue its study in FLC, especially due to its putative role in aberrant PKA signaling, a cAMP-dependent pathway that is abnormal in virtually all FLC tumors.

Beyond LINC00473, this dataset is currently being actively harnessed in a hypothesis-generating manner in several ongoing studies in our lab aimed at evaluating potential therapeutic vulnerabilities in FLC. These include the functional examination of SLC16A14, an orphan transporter, as well as the investigation of the suppressed pathways in retinoic acid metabolism, an otherwise normal function of the healthy liver. This dataset has also been shared with multiple collaborators and their groups are actively pursuing several additional gene targets and pathways. Importantly, our lab is currently integrating this RNA-seq dataset in several multiomic analyses, including proteomics and metabolomics data on FLC vs NML to provide a clearer understanding of the molecular etiology of FLC.

2. What does RQV stand for in Figure 2, 3? It does not seem to be listed in the figure legend or paper.

We thank the Reviewer for their keen eye for detail for the benefit of our manuscript. RQV is an abbreviation for Relative Quantitative Value, which is the change of any normalized measurement relative to the control group. The RQVs for the controls are shown in the figures and are equal to 1.0. The abbreviation has been added to each legend where it is used.

3. Minor comment that the font on many of the figures is very difficult to read because it is quite small.

We completely agree with the Reviewer that the font size of some of the figures is small and makes the text challenging to view. The figures have been enlarged to improve legibility.

4. "These observations provide strong evidence that LINC00473 expression is driven by the DP fusion in FLC." I think the data from Figure 2 really just demonstrates that LINC00473 expression in FLC requires DP fusion. This statement would be true if showing that the DP fusion specifically leads to LINC00473 expression. Demonstrating overexpression of the DP fusion in wild-type liver cells, then asking whether there is increased LINC00473 expression is a critical sufficiency experiment. The current experiment in the manuscript expresses LINC00473 in FLC cells that already have expression of LINC00473 – it is unclear to me what extra levels of LINC00473 does to the cells.

We thank the Reviewer for their valuable input. We especially appreciate their efforts in providing insightful feedback on possible experimental approaches. Indeed, a similar method was under heavy consideration in the development of this project. However, engineering LINC00473 overexpression in human hepatocytes is notoriously difficult, given that hepatocytes do not proliferate or survive in tissue culture for more than a few days. Follow-up phenotyping would be an even greater challenge.

To validate that the DP fusion induces LINC00473 expression, we and collaborators used CRISPR-Cas9 engineering to endogenously generate the oncofusion in two cell lines that otherwise have little or undetectable levels of LINC00473. These include HEK293-DP cells (Kim et al., 2022, PLOS One) and HepG2-DP cells (Nukaya, 2023, bioRxiv). We found that LINC00473 levels were robustly elevated in response to the DP fusion in HepG2-DP cells compared to non-targeting guide control (HepG2-Ctl; Figure 1H) and in HEK293-DP cells compared to wild-type control (HEK293-WT; Supplementary Figure 1C).

5. The proliferation experiments are purely based on cell counts – would be helpful to see a measure of viability using ATP-based readout assays such as Cell-Titer-Glo (CTG) for these measurements or using doubling time as a proxy rather than total cell number. Is this the viable cell count or just the total cell number?

We are grateful to the Reviewer for their suggested strategies to validate the cell count data. Although Cell Titer Glo is a commonly used assay, ATP measurements serve as a proxy for cell proliferation as the reviewer mentioned. In the context of our study, a key consideration persuaded us to forgo CTG for measuring cell growth. Specifically, our Seahorse experiments have demonstrated that LINC00473 plays a role in both major branches of ATP production by promoting glycolysis and altering cellular respiration. These observations indicate that the CTG assay may potentially measure altered levels of LINC00473-related metabolic activity and provide a poor indication of cell proliferation.

As per the Reviewer's suggestion, we also provide doubling time data below and as part of a revised Figure 3D, E, F, and G. We also wish to clarify that our cell count data depicts the total cell number, which we revised in the figure legend and methods section.

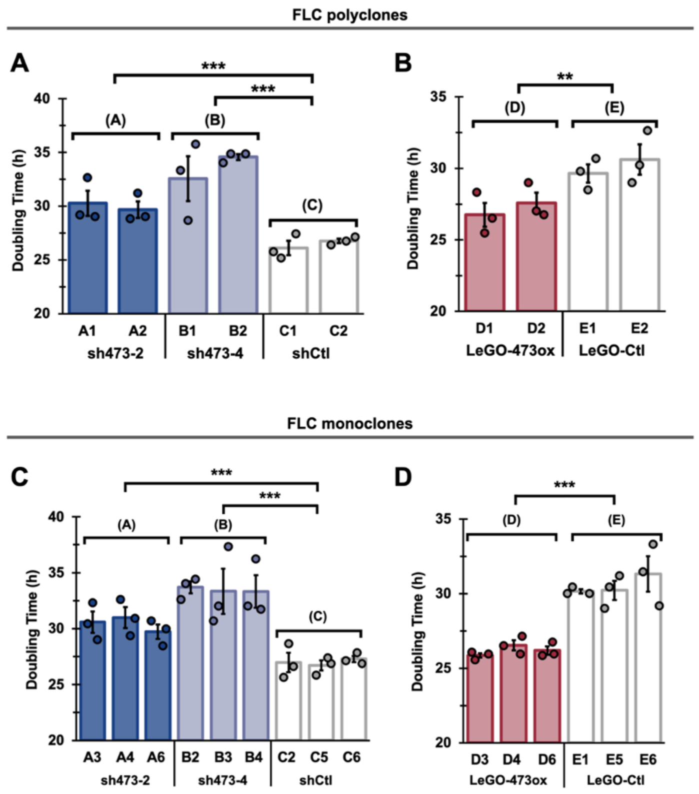

Cell doubling time of FLC cells. Doubling time (hours) of FLC polyclonal cells with stable LINC00473 knockdown (sh473-2, sh473-4) and non-targeting control (shCtl) (**A**) or overexpression using LINC00473 cDNA (LeGO-473ox) and empty vector control (LeGO-Ctl) (**B**). Each polyclonal cell line was quantified 2 times across independent passages (A1, B1, C1 from passage 1; A2, B2, C2 from passage 2), and each point is the average doubling time of 3 replicates. Doubling time (hours) of FLC monoclonal cells with stable LINC00473 knockdown (sh473-2: clones A3, A4, A6; sh473-4: clones B2, B3, B4) and non-targeting control (shCtl: clones C2, C5, C6) (**C**), or overexpression (LeGO-473ox: clones D3, D4, D6) and empty vector control (LeGO-Ctl: clones E1, E5, E6) (**D**). Each monoclonal cell line was quantified 2 times across independent passages, and each point is the average doubling time of 3 replicates.

6. In Figure 4A, it would be preferable to visualize the data using a volcano plot with log(fold change) and then replace the mean normalized counts with significance (q-value/FDR). The mean normalized counts are generally not useful for these comparisons. It would be helpful, moreover, to have the RNA-seq done in triplicate.

To provide a different means of data visualization in addition to the MA plot in the manuscript, a volcano plot has been generated below. To address the comment on performing the RNA-seq in triplicate, we agree that increased sample numbers would have benefited our study. However, repeating this experiment to include an additional replicate may not add very much value to the analysis and would probably lead to very similar conclusions. Due to cost and time constraints surrounding RNA-seq studies, we deliberated between repeating this experiment or conducting an entirely new study on LINC00473 overexpression in PDX tumors as outlined in the Reviewer's Point #8. Ultimately, we chose the latter experiment in order to more deeply explore the gene regulatory programs of the lncRNA in the in vivo context. We thank the reviewer for their consideration.

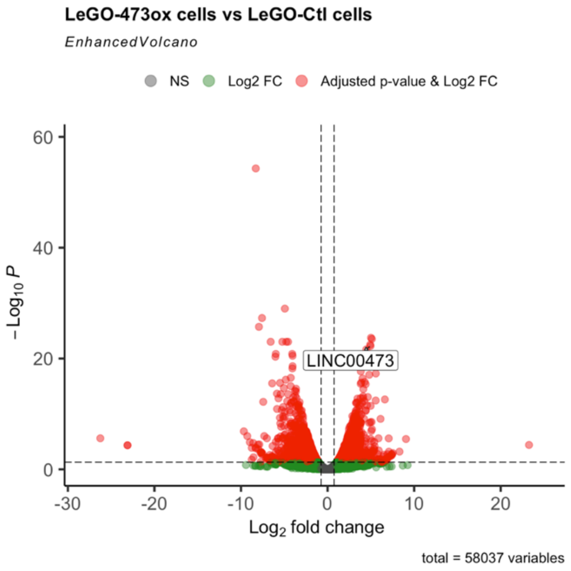

7. 8 days is quite short for a PDX experiment – do the authors have additional data showing that this is an ongoing sustained increase in tumor volume?

We thank the Reviewer for their interest in our in vivo experiments. The study concluded on Day 8 due to large tumor burden, and some animals approached humane euthanasia endpoint. For instance, some of the largest tumor masses at the endpoint were above 2000mg, with the largest at 3167mg. We agree with the Reviewer that additional data over a longer experimental window would provide further support to demonstrate that LINC00473 increases tumor volume. However, under the expert advisement of the Surgery Manager and PDX surgical specialists at the Cornell College of Veterinary Medicine PDX facility, the rapid growth of tumors presents a direct challenge to animal welfare beyond eight days of experimentation.

8. Do the authors have tumor tissue from these experiments that was processed for RNA-sequencing? This would give a better sense in an in vivo model of what LINC00473 transcriptionally activates.

We express our gratitude to the Reviewer for their constructive comments and curiosity about investigating the differential gene expression programs in tumors generated from LINC00473 overexpressing (LeGO-473ox) cell implantation. To explore the specific genes and pathways that

LINC00473 activates in the in vivo context, we took the advice of the Reviewer and performed RNA-sequencing on PDX tumors harboring LeGO-473ox and LeGO-Ctl empty vector control tumors (n = 4 for both groups). These experiments used remaining tumor tissues from previous experiments reported in our manuscript.

Our RNA-seq analyses identified notable upregulated genes including FGF13, MMP16, BMP2, TOX3, PTPRD PLTP, PRKCB, CA8, HSPA2, and SPOCK1 because of their associated roles in various cancers. Suppressed genes of interest include TSSC2, SPINT2, TSC22D3, HIC1, FASN, ITPR3, and PCK1 due to their association with tumor suppression and positive regulation of apoptosis. We have generated an MA plot and PCA plot (Figure 6D and 6E). These data from this RNA-seq experiment have been deposited into GEO and are accessible at GSE253465.

To explore the biological significance of these differentially expressed genes, we performed gene pathway enrichment analysis using Enrichr using the top up- and down-regulated genes between LeGO-473ox compared to LeGO-Ctl. These genes were identified with the following thresholds: base mean > 100; log2 fold change > 0.5 for upregulated genes or < –0.5 for downregulated genes; adjusted p-value < 0.05. Below, we provide a snapshot of the most strongly activated and suppressed pathways that result from LINC00473 overexpression in implanted FLC tumors. Further, these data have been added to Figures 6F and 6G of the manuscript (MSigDB) or Supplementary Figures 6B and 6C (KEGG).

Top Upregulated Pathways (n = 953 genes)

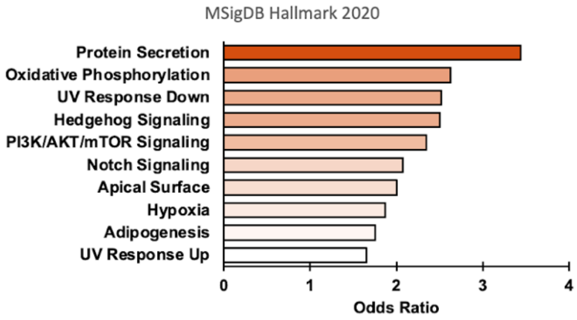

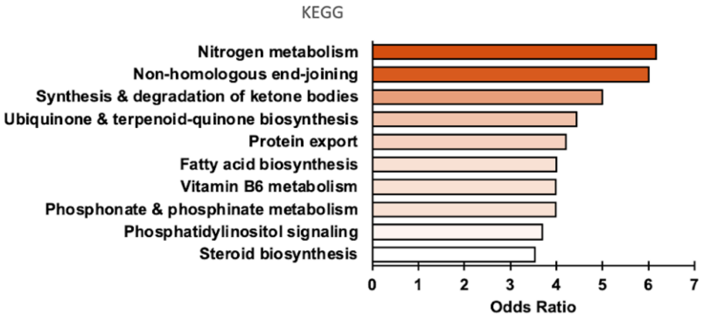

Top Downregulated Pathways (n = 1468 genes)

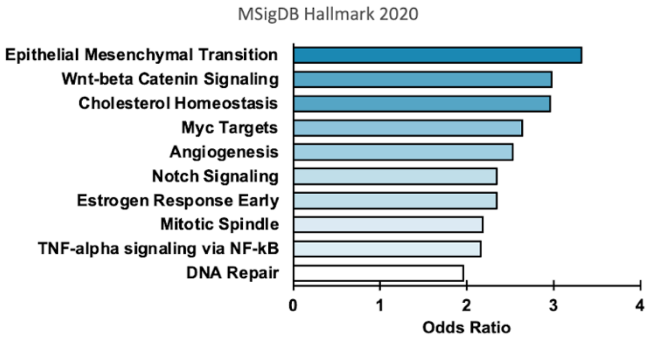

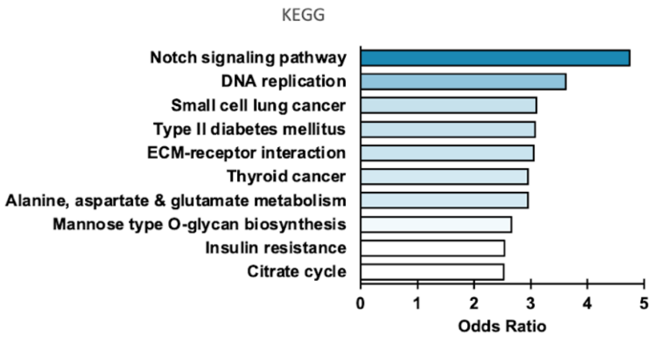

The main conclusions from this analysis include:

- LINC00473 overexpression in FLC-derived tumors activates oxidative phosphorylation pathways, which is consistent with our metabolic assay observations and suggests that the lncRNA plays a role in mitochondrial energetics in FLC. Other notable upregulated pathways include UV Response

Down, Hedgehog Signaling, PI3K/AKT/mTOR Signaling, and Hypoxia (Figure 6F) because they were found to be activated in FLC cells harboring LINC00473 overexpression (Figure 4E) or enriched in the overlapping genes in primary FLC tumors and LeGO-473ox cells relative to their respective controls (Figure 4I).

- In contrast, elevated levels of LINC00473 led to the suppression of several pathways within the FLC cell-derived tumor context. This includes suppressed DNA repair and TNF-alpha Signaling via NF-kB (Figure 6G), which were previously noted in earlier analyses (Figure 4F and J). Additionally, p53 signaling and apoptosis were strongly downregulated (Supplementary Figure 4E), which is consistent with previous findings (Figure 4F and 4J) and provides further evidence that LINC00473 plays a role in suppressing apoptosis.

9. It would help to have a supplemental table of the most significant differentially expressed genes for RNA-seq studies.

We completely agree with the Reviewer that a supplemental table of significantly expressed genes would be beneficial for readers. To this end, three supplementary tables have been added to reflect the three RNA-seq experiments in this study. Up- and down-regulated genes are delineated by the tabs within each spreadsheet. These are named as follows and are provided as part of the Supporting Information:

- DEG on primary patient tissues, FLC tumors vs. NML: “Most significant DEGs_FLC vs NML.xlsx”
- DEG on engineered cells, LeGO-473ox vs. LeGO-Ctl cells: “Most significant DEGs_LeGO-473ox vs LeGO-ctl cells.xlsx”
- DEG on PDX tumors generated from cell implantation, LeGO-473ox vs. LeGO-Ctl tumors: “Most significant DEGs_LeGO-473ox vs LeGO-ctl tumors.xlsx”

Overall, I think the functional approaches utilized are robust and well controlled. While CRISPR-Cas9 approaches would be preferable, the siRNA approaches are reasonable. A major issue with the manuscript is that the figures are very difficult to read at 100% and for nearly all figures, I had to zoom in to 200-300% to read the figure legends. It would help the reader significantly for these legends to be more clear and refer specifically to the experiment (for example, if referring to qPCR data on DP fusion, to specifically show that in the figure). Otherwise, most of my concerns are around experimental details as detailed above.

We appreciate the Reviewer’s thoughtful comments and careful examination of our work. To address the readability of the figure legends, the font size has increased. The legibility of figure panels has previously been addressed in an earlier comment. We thank the Reviewer for their valuable suggestions that have improved the quality of our manuscript.
