# Supplementary material for: DNAJB1-PRKACA fusion protein-regulated LINC00473 promotes tumor growth and alters mitochondrial fitness in fibrolamellar carcinoma
Source: PLoS Genet. 2024 Mar 21;20(3):e1011216. doi: 10.1371/journal.pgen.1011216 (PMC11020935; doi:10.1371/journal.pgen.1011216)
Supplement: Attachment — Submitted filename: Reviewer Attachments-PGENETICS-D-23-01036-PG.pdf [file pgen.1011216.s012.pdf]

Critique

The finding that LINC00473 as critical downstream driver of cell growth and survival in FLC harboring DNAJB1-PRKACA fusions both in PDX models and cell lines is an important contribution to understanding the gene networks that drive FLC. The study comprises the largest number of FLC samples analyzed through RNA-seq dataset published to date.
The work done to establish this especially with the addition of CRISPR- Cas9 to endogenously generate the DNAJB1-PRKACA fusion which was shown to significantly upregulated LINC00473 in HEK293-DP cells HepG2-DP cells with low or undetectable levels of the LncRNA provides strong evidence that LINC00473 is a downstream target of the onco-fusion.

Actionable fusions carrying one or both genes that have an FDA approved drug have significantly advanced cancer treatment. However, toxicities associated with the impact of these drugs on normal tissues expressing the wild-type form of the actionable gene has remains a major challenge. In that context finding critical drivers of oncogenic phenotypes downstream of actionable fusions is a very important next step. Functional studies to explore the impact of knock-down and upregulation of LINC00473 provides evidence for developing therapeutic strategies to impact the metabolic pathways driving FLC. RNAi-based therapies are currently in clinical trials however will take longer to get approved. In that context the finding that LINC00473 is also downstream of the fusion oncoprotein CRTC1-MAML2 in mucoepidermoid carcinoma (MEC) is mentioned as an opportunity for the work presented to have impact broader impact beyond FLC which is a rare cancer. In that context the manuscript would have strengthened in bringing in downstream drivers of LINC00473 as new druggable targets for FLC. It is mentioned in the discussion that MUC5B and MUC14 established to be tumor-specific mucins in MEC and biliary tract carcinoma, are among the most highly expressed genes in FLC from RNA-seq. siRNA studies targeting MUC5B and MUC14 is able to rescue to oncogenic phenotype of either, in FLC cell lines carrying a DNAJB1-PRKACA-knock down and/or LINC00473.
