# Supplementary material for: DNAJB1-PRKACA fusion protein-regulated LINC00473 promotes tumor growth and alters mitochondrial fitness in fibrolamellar carcinoma
Source: PLoS Genet. 2024 Mar 21;20(3):e1011216. doi: 10.1371/journal.pgen.1011216 (PMC11020935; doi:10.1371/journal.pgen.1011216)
Supplement: Attachment — Submitted filename: Response to Reviewers_PLOS Genetics_Final.pdf [file pgen.1011216.s013.pdf]

Editor's comments:

Your revised manuscript has been evaluated by the three reviewers. All reviewers note significant improvement in your manuscript. There are several minor points of revision that are needed at this time. These points can be addressed through revising your manuscript text to discuss the research findings in the way suggested by the reviewers. **Please adjust the language around GSEA/pathway analyses as suggested and clarify how the overexpression of LINC00473 compares to the overexpression of other genes in this disease.** We will be happy to consider your revised manuscript. Please submit a point-by-point response to the reviewers' comments. Assuming your responses are sufficient, we anticipate moving forward with your paper.

We are delighted with the enthusiasm for our work and very positive reviews. We have meticulously examined each of the reviewers' comments and offer detailed point-by-point responses below. Importantly, we revised the manuscript to clarify the language regarding our pathway analyses and LINC00473 as an upregulated gene in FLC to enhance accuracy. These changes are highlighted in the document "Manuscript Resubmission_LINC00473_highlighted changes.pdf".

Comments to the Reviewers:

Reviewer #1:

It was my pleasure to review the revised manuscript entitled "DNAJB1-PRKACA fusion protein-regulated LINC00473 promotes tumor growth and alters mitochondrial fitness in fibrolamellar carcinoma" by Ma and colleagues. The authors took extraordinary measures to address reviewer comments, bolstering the use of the initial RNA-Seq cohort for discovery purposes and exploring possible hypothesis. All this reviewer's original comments were addressed in the revised manuscript.

The revised manuscript includes numerous additional analyses, including gene set/pathway analyses that utilized enrichR and GSEA. I noted that in the rebuttal document and in the revised text the authors interpreted ranked lists of GSEA results.

I would urge the authors to exercise caution when interpreting GSEA results with p-values or adjusted p-values that are not significant. If statistical significance is borderline, one could increase the number of GSEA permutations to obtain a higher precision significant estimate. Otherwise, I would interpret the results as not enriched. I would not attempt to draw meaningful conclusions from these results, which could occur due to chance, and instead state that the results were not significant. This would leave room for future follow-up studies exploring the role of LINC00473 in FLC.

I appreciate the extraordinary effort by the authors to provide a polished, high quality manuscript for publication.

We are grateful for the Reviewer's enthusiastic and thoughtful response to our revised manuscript and additional analyses. We fully acknowledge and agree with the Reviewer's recommendation to exercise caution in overinterpreting insignificant or borderline-significant enrichment results. We have carefully reviewed our manuscript and can confirm that the pathways we have discussed (Figures 4E, 4F, 4I, 4J, 6F, 6G and Supplementary Figures 4D, 4E, 5B, 5C, 5D, 5E, 6B, 6C) are robustly statistically significant. To clarify this in our manuscript, we revised the following lines:

- *"Strikingly, genes upregulated by LINC00473* were most highly enriched *in metabolic pathways, including glycolysis (MSigDB, Figure 4E), pentose phosphate pathway, galactose metabolism, and mitochondrial respiratory chain dysfunction (Supplementary Figure 5B)."*
    - This is revised to, *"Strikingly, genes upregulated by LINC00473* were significantly enriched *in metabolic pathways, including glycolysis (MSigDB, Figure 4E), pentose phosphate pathway, galactose metabolism, and mitochondrial respiratory chain dysfunction (Supplementary Figure 5B)."*

- *“We also found that genes downregulated by LINC00473 were most enriched in apoptosis and p53 pathways (MSigDB, Figure 4F; Supplementary Figure 5C).”*
    - This is revised to “*We also found that genes downregulated by LINC00473 were significantly enriched in apoptosis and p53 pathways (MSigDB, Figure 4F; Supplementary Figure 5C).”*

Reviewer #2:

The finding that LINC00473 as critical downstream driver of cell growth and survival in FLC harboring DNAJB1-PRKACA fusions both in PDX models and cell lines is an important contribution to understanding the gene networks that drive FLC. The study comprises the largest number of FLC samples analyzed through RNA-seq dataset published to date.
The work done to establish this especially with the addition of CRISPR- Cas9 to endogenously generate the DNAJB1-PRKACA fusion which was shown to significantly upregulated LINC00473 in HEK293-DP cells HepG2-DP cells with low or undetectable levels of the LncRNA provides strong evidence that LINC00473 is a downstream target of the onco-fusion.

Actionable fusions carrying one or both genes that have an FDA approved drug have significantly advanced cancer treatment. However, toxicities associated with the impact of these drugs on normal tissues expressing the wild-type form of the actionable gene has remains a major challenge. In that context finding critical drivers of oncogenic phenotypes downstream of actionable fusions is a very important next step. Functional studies to explore the impact of knock-down and upregulation of LINC00473 provides evidence for developing therapeutic strategies to impact the metabolic pathways driving FLC. RNAi-based therapies are currently in clinical trials however will take longer to get approved. In that context the finding that LINC00473 is also downstream of the fusion oncoprotein CRTC1-MAML2 in mucoepidermoid carcinoma (MEC) is mentioned as an opportunity for the work presented to have impact broader impact beyond FLC which is a rare cancer. In that context the manuscript would have strengthened in bringing in downstream drivers of LINC00473 as new druggable targets for FLC.

It is mentioned in the discussion that MUC5B and MUC14 established to be tumor-specific mucins in MEC and biliary tract carcinoma, are among the most highly expressed genes in FLC from RNA-seq. siRNA studies targeting MUC5B and MUC14 is able to rescue to oncogenic phenotype of either, in FLC cell lines carrying a DNAJB1-PRKACA-knock down and/or LINC00473.

We want to extend our gratitude to the Reviewer for their thoughtful and enthusiastic assessment of our manuscript. We acknowledge the importance of identifying readily actionable downstream genes of LINC00473 for the potential benefit of patients with FLC, MEC, and biliary tract carcinoma. However, we would like to highlight that the scope of our current work does not encompass the exploration of this specific question, including the investigation of MUC5B and MUC14 as candidate targets. At the time of writing, it is unknown whether the mucin genes are up or downstream of LINC00473. Nevertheless, we recognize the importance of uncovering this relationship and agree that dissecting the therapeutic vulnerability of these tumor-specific mucins holds promise for intriguing future investigations. To that end, we have added a sentence to the Discussion section to highlight this important research direction in the future*: “Further, it is important to uncover the downstream targets and effectors of LINC00473 in FLC and other cancers.”*

Reviewer #3:

1. Appreciate the authors' explanation but I agree with Reviewer 1's comments that LINC00473 does not appear to be the major downstream target of the DNAJB1-PRKACA fusion. It appears that the RNA-seq data was just used as more supporting data for the initial hypothesis. It would help if this was clearly stated in the manuscript in the description of the RNA-seq results (it appears to be #124 most upregulated gene in the list of differential expression...)

We appreciate the Reviewer's thorough assessment of our work. We completely agree with the Reviewer's recommendation to enhance the clarity of our findings. Specifically, we have revised the language concerning our RNA-seq data to more accurately convey that LINC00473 is elevated in FLC without implying that it is among the topmost upregulated genes.

The following sentence has been revised: *"Our analysis identified LINC00473 as one of the top-most significantly upregulated genes in FLC compared to NML (fold-change > 25, Figure 1A), though there is some variance of expression values across patients (Figure 1B)."* The modified sentence now reads: *"Our analysis determined that LINC00473 is significantly elevated in FLC compared to NML (fold-change > 25, Figure 1A), though there is some variance of expression values across patients (Figure 1B)".*

Additionally, we revised the title of Figure 1 from, "*Figure 1. RNA-seq on an expanded number of primary patient samples reveals LINC00473 as a top up-regulated gene in FLC.*". The title now reads, "*Figure 1. RNA-seq on an expanded number of primary patient samples reveals LINC00473 as a significantly up-regulated gene in FLC.*"

We believe that this textual adjustment better represents our results and addresses the Reviewer's helpful feedback. Hopefully, it is now clear to the reader that we did not pursue LINC00473 because it is the *most* up-regulated gene but rather because it is a gene that has been implicated previously as an oncogene in non-small cell lung cancer (Chen et al., 2016, JCI) and it is very highly elevated in FLC across patients (even more so than what was observed before in non-small cell lung cancer).

2. For the new data on tumors from LINC00473 cell implantation – MSigDB Hallmark and KEGG pathways are often somewhat generic. Was there any examination of the C3 regulatory gene sets for example that specifically look at transcription gene sets and miRNA gene sets. There are known miRNAs that LINC00473 regulates, and it would be a reasonable validation to see if those are reflected in the models generated.

We extend our gratitude to the Reviewer for their thoughtful inquiry into our new RNA-seq dataset, as it provides us with an opportunity to delve deeper into the dataset and gain more meaningful insights. To explore associations in gene sets centered on transcription factors or miRNAs, we used (i) ENCODE and ChEA Consensus TFs from ChIP-X, and (ii) miRTarBase datasets, which contain experimentally determined TF or miRNA targets.

By ranking the top transcription factors by adjusted p-value using the ENCODE and ChEA Consensus dataset, we found that specific categories of TF function emerged. Using an adjusted p-value less than 0.05 benchmark, we note that only 7 statistically significant TFs emerged from the upregulated gene list (n=955 genes) and 35 TFs surfaced from the list of downregulated genes (n=1468). By analyzing these TFs, prominent categories of cellular function emerged, including:

Upregulated gene list:

- Cancer-related pathways (PI3K/Akt, TGFB signaling)
- Cell cycle regulation
- Embryonic stem cell maintenance
- Oxidative stress response
- Immune response

Downregulated gene list:

- Tumor suppression/anti-apoptosis and negative regulation TGFB signaling
- Chromatin organization

- Epigenetic regulation
- Cell cycle regulation
- Immune response
- Immune cell development

Interestingly, ZBTB7A stood out as the topmost TF associated with downregulated genes in LeGO-473ox tumors relative to control. In our discussion section, we previously noted that Zhang et al. and Ruiz-Bañobre et al. found that ZBTB7A silences LINC00473 transcription by directly binding to the lncRNA promoter (PMID: 28942243, PMID: 35810318). It is intriguing to consider the idea that the overexpression of LINC00473 leads to decreased ZBTB7A-driven transcription. Though further rigorous study is needed, this finding may indicate that LINC00473 functions in a positive feedback loop to negatively regulate its own silencer to decrease its expression and/or activity. Another possibility, which is not mutually exclusive with the aforementioned hypothesis, is that DNAJB1-PRKACA silences ZBTB7A to elevate LINC00473. We summarize these data in the tables below, with TFs ranked by adjusted p-value.

Color Key:						
**Cancer-related signaling pathway**	**Chromatin remodeling**	**Epigenetic regulation**	**Cell cycle and growth**	**Immune response and inflammation**	**Stem cell maintenance and development**	**Other**

Transcription Factors associated with top upregulated genes (n=955):

Name	P-value	Adjusted p-value	Odds Ratio	Combined score	Regulated pathways
AR	2.05E-08	0.000002126	1.97	34.84	Androgen signaling, PI3K/Akt pathway
SUZ12	0.0000536	0.002787	1.52	15	Epigenetic regulation
SMAD4	0.0003441	0.01193	1.78	14.23	TGF-β signaling
SOX2	0.001355	0.02819	1.59	10.48	Embryonic stem cell maintenance
E2F1	0.001283	0.02819	1.56	10.39	Cell cycle regulation
NFE2L2	0.002315	0.04013	1.48	8.97	Oxidative stress response
ELF1	0.003085	0.04583	1.3	7.52	Immune response, cell cycle regulation

Transcription Factors associated with top downregulated genes (n=1468):

Name	P-value	Adjusted p-value	Odds Ratio	Combined score	Regulated pathways
ZBTB7A	1.21E-23	1.26E-21	2.11	111.35	Negative regulation of TGFB signaling
CTCF	2.73E-16	1.42E-14	1.95	69.91	Chromatin organization, insulator function
SUZ12	2.38E-10	8.26E-09	1.72	38.08	Epigenetic regulation
EZH2	1.00E-09	2.59E-08	3.11	64.37	Histone methylation
SMC3	1.25E-09	2.59E-08	1.82	37.29	Cohesin complex, chromatin organization
KLF4	1.39E-07	0.000002406	1.76	27.76	Cell differentiation
UBTF	0.00000311	0.00004621	1.51	19.12	Ribosomal RNA transcription
E2F6	4.155E-06	0.00005401	1.37	16.92	Cell cycle regulation
CEBPD	5.827E-06	0.00006733	1.74	20.99	Immune response, inflammation
RCOR1	0.00001844	0.0001917	1.71	18.63	Epigenetic regulation
GATA1	0.00002567	0.0002334	1.64	17.38	Hematopoiesis regulation
SOX2	0.00002693	0.0002334	1.66	17.43	Embryonic stem cell maintenance
BCL3	0.0001044	0.0008356	2.07	18.97	Immune response
RUNX1	0.0001962	0.001458	1.44	12.26	Hematopoiesis regulation
TCF3	0.0002241	0.001553	1.49	12.52	Immune cell development (B and T cell)
STAT5A	0.0004498	0.002924	2.03	15.66	Anti-apoptotic function via BCL-XL induction
NELFE	0.0004852	0.002968	2.02	15.43	RNA polymerase II regulation
NFYB	0.0005267	0.003043	1.25	9.42	Transcriptional regulation

TCF3	0.001029	0.00561	1.47	10.09	Immune cell development (B and T cell)
GATA2	0.001079	0.00561	1.49	10.15	Hematopoiesis regulation
PML	0.001172	0.005803	1.33	9	Tumor suppression
E2F4	0.001399	0.006614	1.49	9.82	Cell cycle regulation
POU5F1	0.001567	0.007087	1.85	11.92	Pluripotency, embryonic stem cell maintenance
EGR1	0.001733	0.007508	1.75	11.13	Early growth response, stress response
PBX3	0.002267	0.009429	1.35	8.2	Neuron development
NFIC	0.002489	0.009958	1.77	10.61	Regulation of immune response, development
RAD21	0.002811	0.01083	1.34	7.86	Cohesin complex, chromatin organization
TRIM28	0.003151	0.0117	1.88	10.82	Epigenetic regulation
ZMIZ1	0.003665	0.01314	1.39	7.77	TGFB signaling regulation
TP63	0.003816	0.01323	1.31	7.29	Tumor suppression
FOXA1	0.008547	0.02867	1.77	8.41	Transcriptional regulation, chromatin opening
EZH2	0.009662	0.0314	1.57	7.27	Histone methylation
ZNF384	0.01288	0.03996	1.36	5.9	Extracellular matrix remodeling
REST	0.01306	0.03996	1.27	5.5	Repression of neuronal genes
CREB1	0.01369	0.04068	1.25	5.36	cAMP response element binding protein
FOSL2	0.01733	0.05006	1.69	6.85	AP-1 complex regulation

To address the Reviewer's query about miRNAs, we used mirTarBase to identify miRNA target sites that are enriched in our gene sets. This revealed 68 and 32 significantly enriched motifs among the top upregulated genes and downregulated genes, respectively. None of these motifs are for miRNAs that we have shown previously to be among the most significantly altered in FLC (PMID: 35482409). Further, we surveyed the literature and found that LINC00473 has not been associated with these miRNAs to date.

In the case the Reviewer may find it helpful, we provided links to our Enrichr analyses below. The Reviewer's engagement with our work is greatly appreciated, and we welcome the chance to enhance the robustness and clarity of our findings in response to their inquiries.

Upregulated LeGO-473ox tumors vs. LeGO-Ctl tumors

- Link to Top Up All genes
  > https://maayanlab.cloud/Enrichr/enrich?dataset=7132d099cf8e45915ec01cf5c84a0a81

Downregulated LeGO-473ox tumors vs. LeGO-Ctl tumors

- Link to Top Down All genes
  > https://maayanlab.cloud/Enrichr/enrich?dataset=52a737c7d86cf1592495154fe839e06c

3. I find it very odd that in the spreadsheet with the most significant differentially expressed genes for tumors that LINC00473 does not appear at all. That is somewhat concerning for these models.

We thank the reviewer for this comment and we acknowledge that clarification is needed in this regard. As mentioned in our manuscript, some annotation libraries erroneously collapsed the LINC00473 transcript into a nearby protein-coding gene called PDE10A. This includes the current GRCh38 human genome assembly, which we used for the differentially expressed gene analysis. To account for this, we typically use a library that correctly annotates LINC00473. For this analysis, we had erroneously used the annotation library without LINC00473. We have corrected this mistake and the revised analysis confirms that LINC00473 is indeed significantly overexpressed (base mean = 1348.46, log2 foldchange = 2.02, padj = 4.20E-16) in LINC00473-overexpressing tumors relative to control (matching what we show by RT-qPCR in Figure 6C). We have added this information to a revised spreadsheet "STable 4. Most significant DEGs_LeGO-473ox vs LeGO-ctl tumors.xlsx".
