# Supplementary figures and images for: Correction: DNAJB1-PRKACA fusion protein-regulated LINC00473 promotes tumor growth and alters mitochondrial fitness in fibrolamellar carcinoma
Source: PLoS Genet. 2026 Feb 26;22(2):e1012054. doi: 10.1371/journal.pgen.1012054 (PMC12944757; doi:10.1371/journal.pgen.1012054)

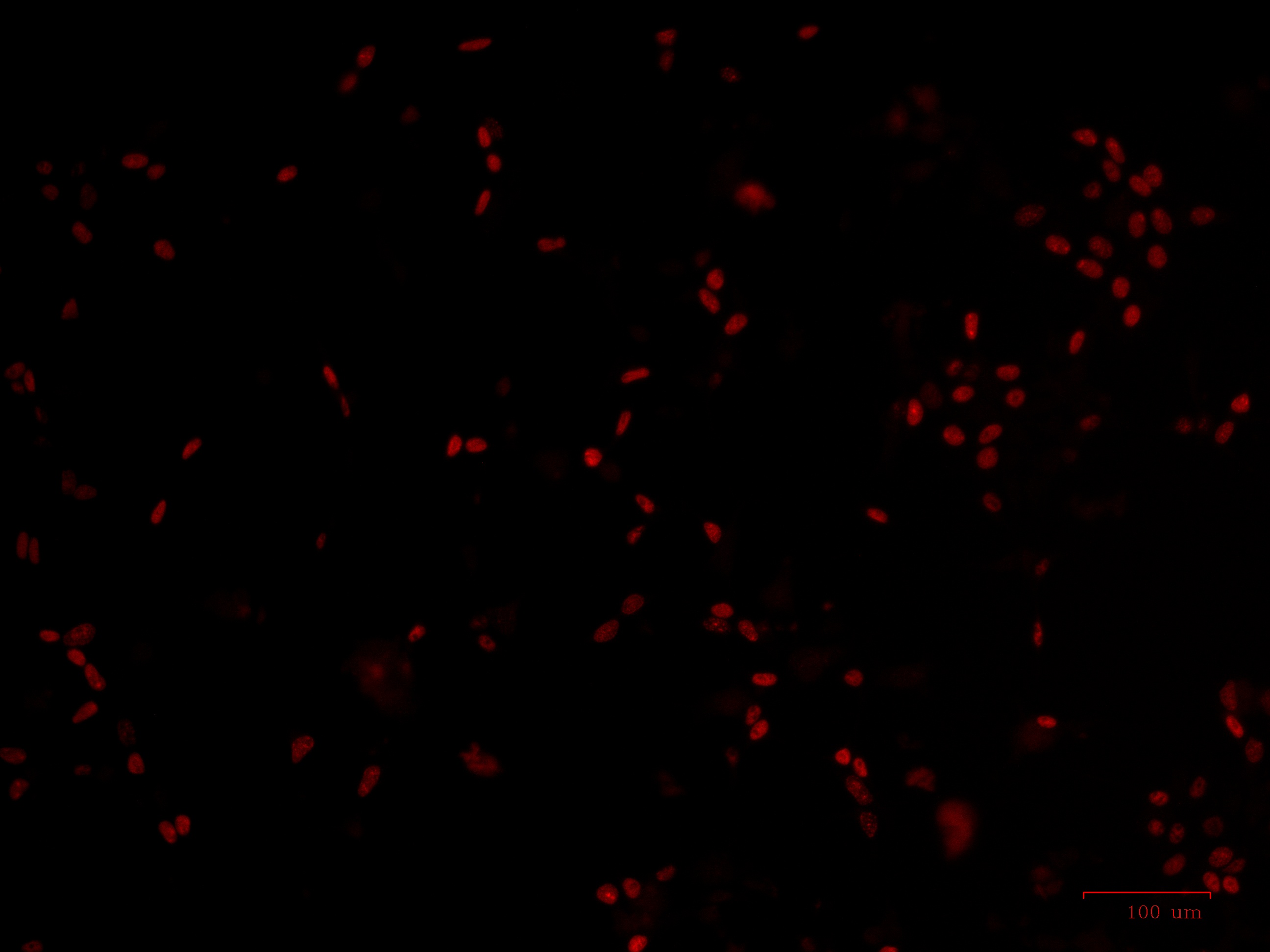

Supplement: S1 File — This file includes the original images underlying Fig 4G; Representative images of DAPI- and EdU-stained FLC cells. (ZIP) [file pgen.1012054.s001.zip › Figure 4G. LeGO-473ox cells, EdU.jpg]

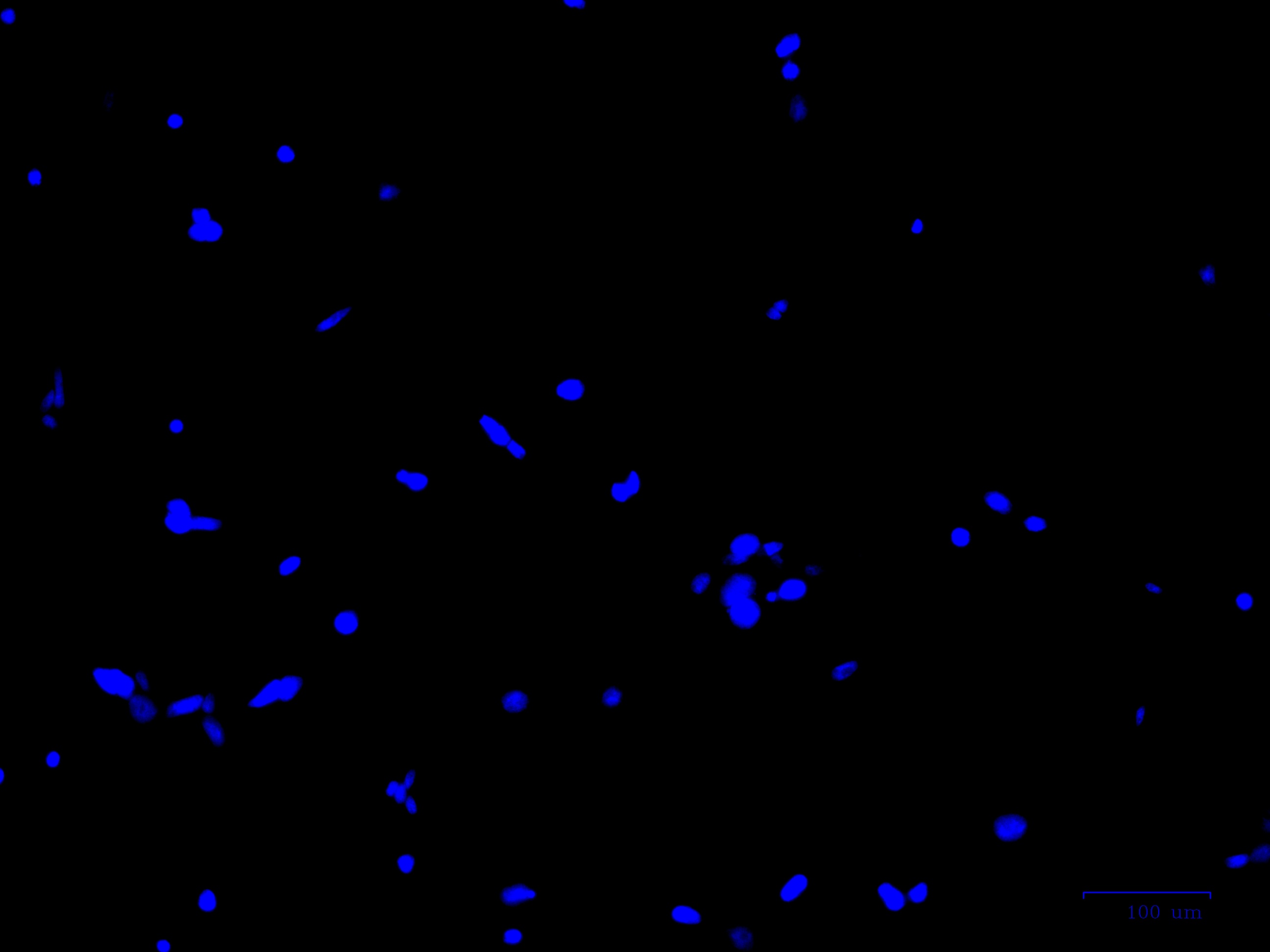

Supplement: S1 File — This file includes the original images underlying Fig 4G; Representative images of DAPI- and EdU-stained FLC cells. (ZIP) [file pgen.1012054.s001.zip › Figure 4G. LeGO-Ctl cells, DAPI.jpg]

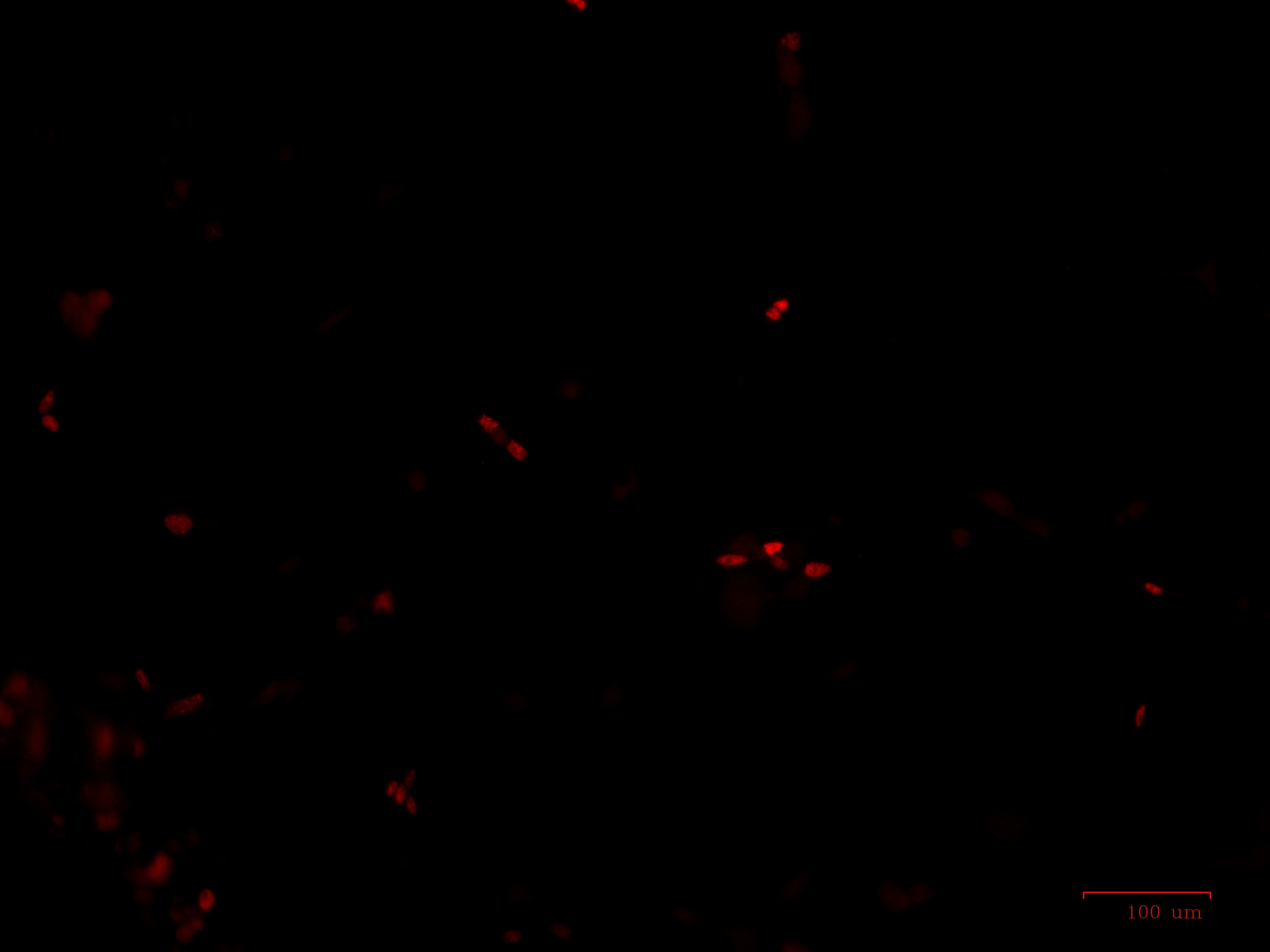

Supplement: S1 File — This file includes the original images underlying Fig 4G; Representative images of DAPI- and EdU-stained FLC cells. (ZIP) [file pgen.1012054.s001.zip › Figure 4G. LeGO-Ctl cells, EdU.jpg]

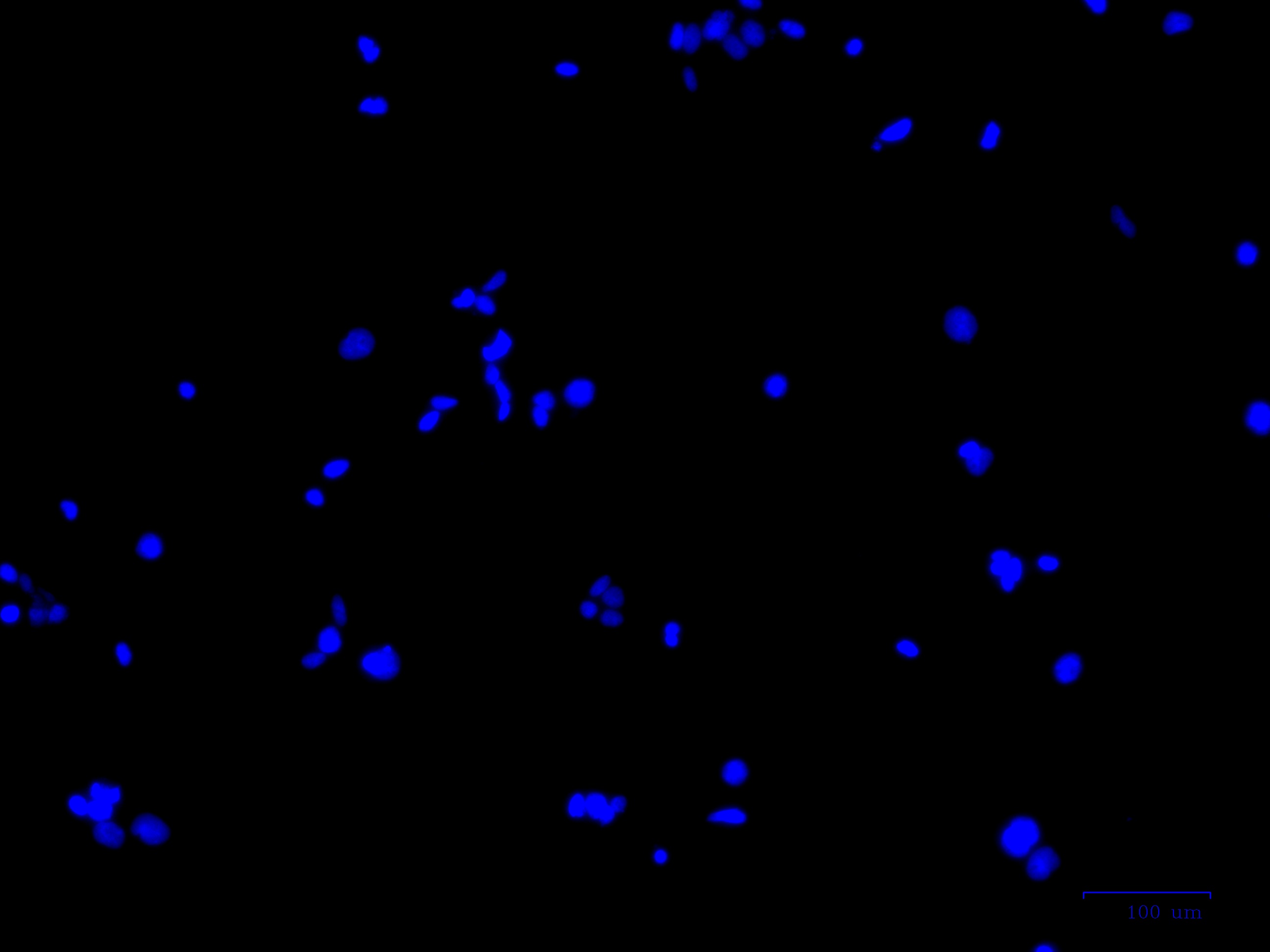

Supplement: S1 File — This file includes the original images underlying Fig 4G; Representative images of DAPI- and EdU-stained FLC cells. (ZIP) [file pgen.1012054.s001.zip › Figure 4G. sh473 cells, DAPI.jpg]

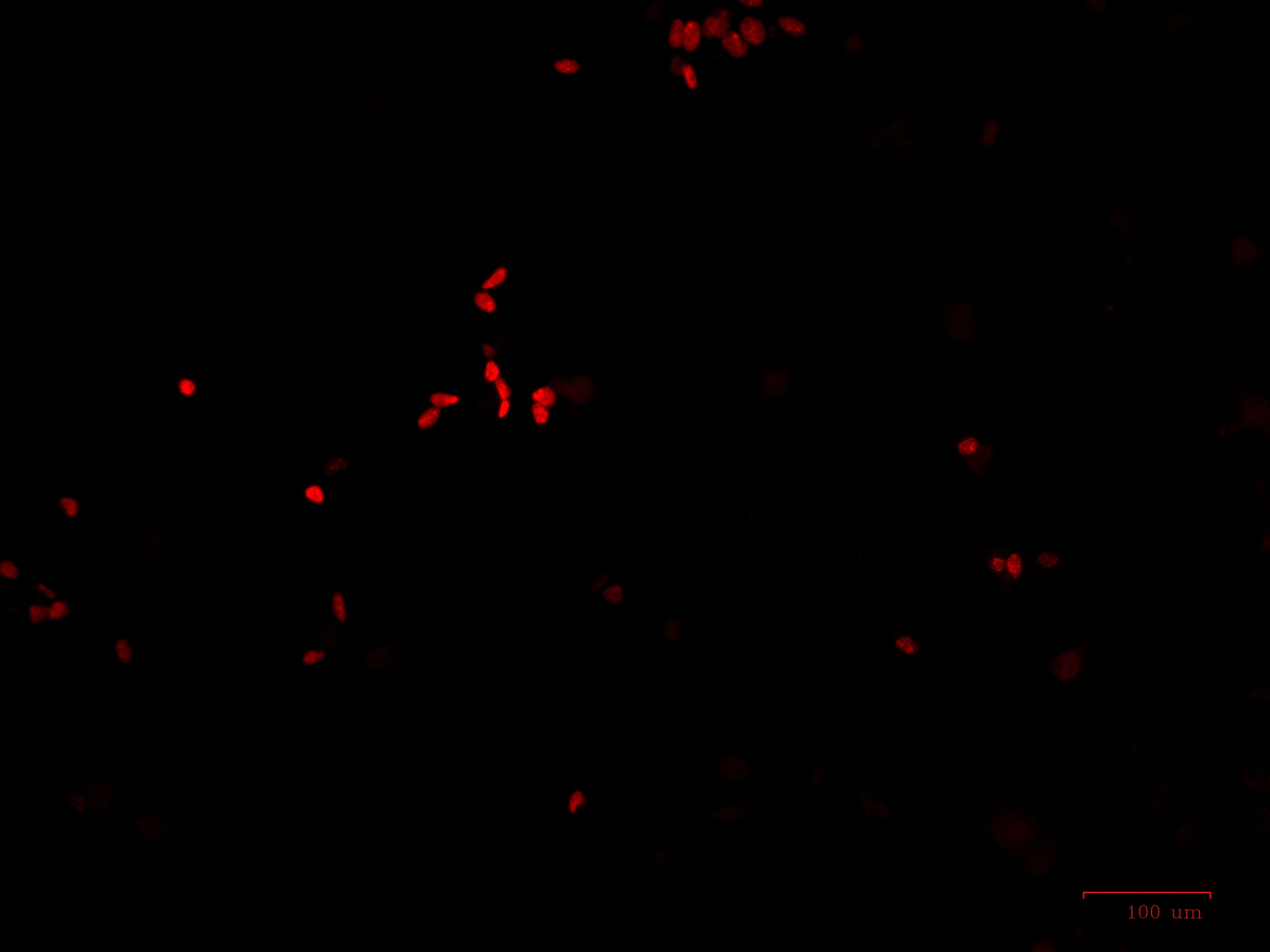

Supplement: S1 File — This file includes the original images underlying Fig 4G; Representative images of DAPI- and EdU-stained FLC cells. (ZIP) [file pgen.1012054.s001.zip › Figure 4G. sh473 cells, EdU.jpg]

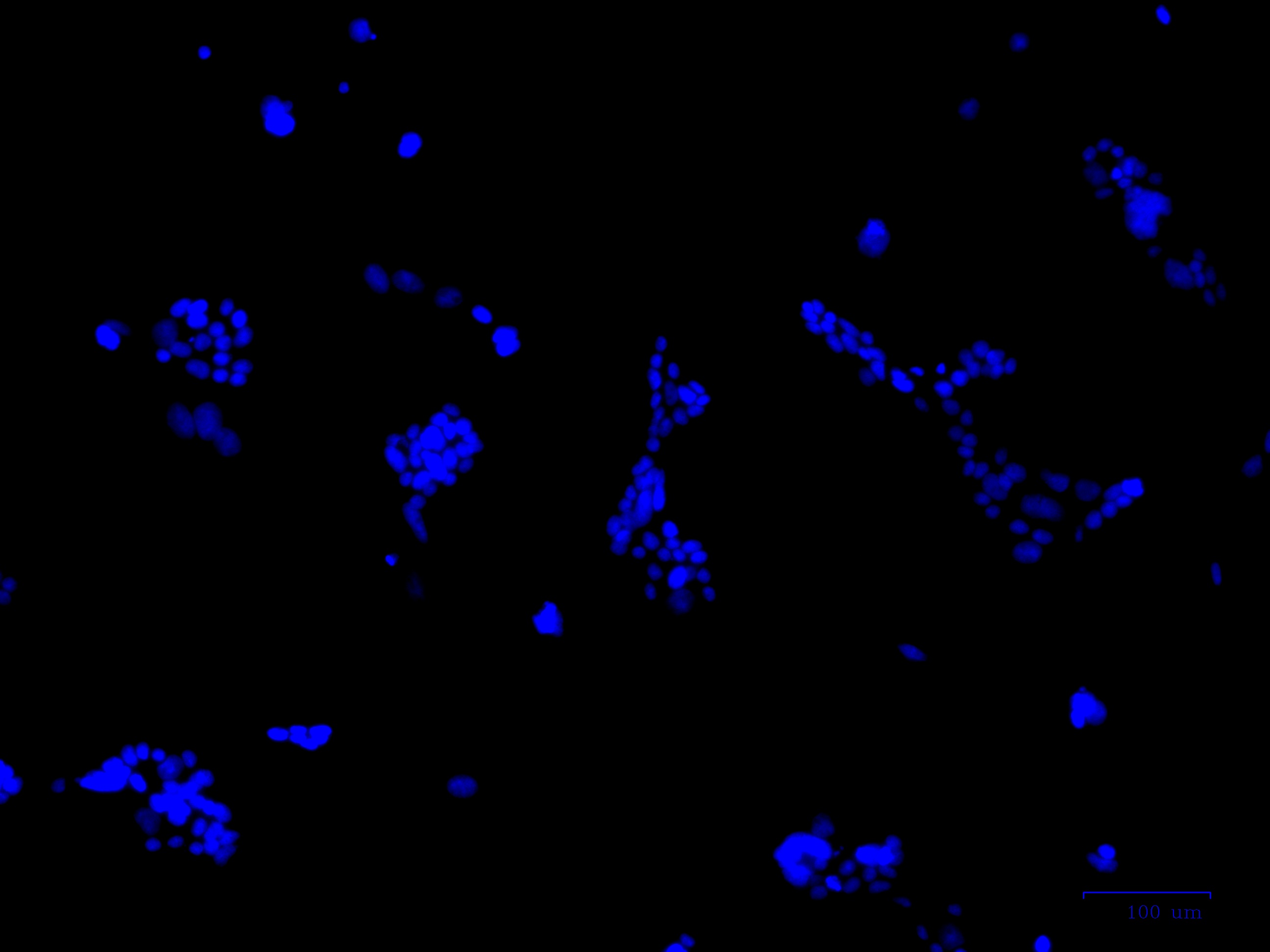

Supplement: S1 File — This file includes the original images underlying Fig 4G; Representative images of DAPI- and EdU-stained FLC cells. (ZIP) [file pgen.1012054.s001.zip › Figure 4G. shCtl cells, DAPI.jpg]

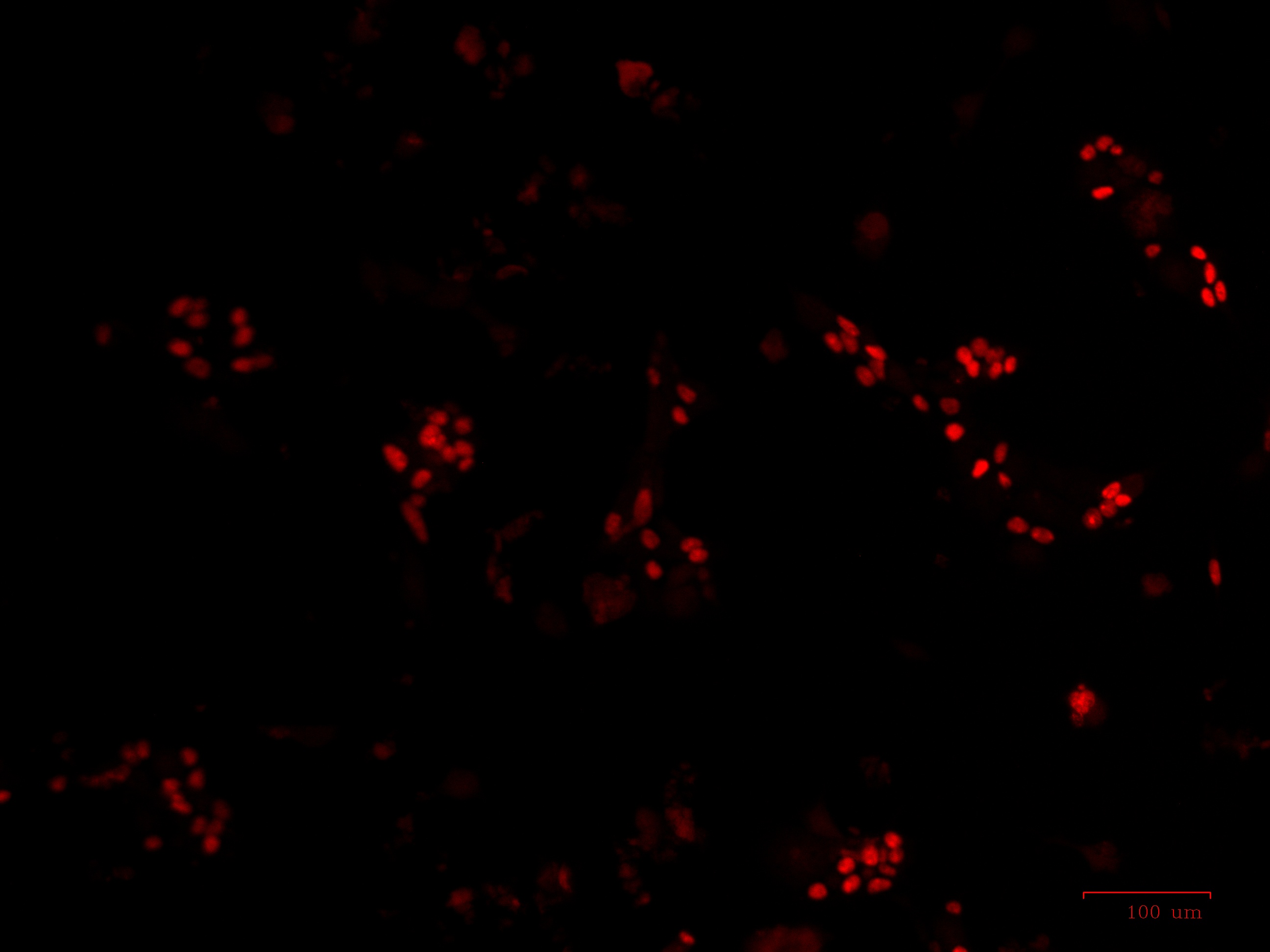

Supplement: S1 File — This file includes the original images underlying Fig 4G; Representative images of DAPI- and EdU-stained FLC cells. (ZIP) [file pgen.1012054.s001.zip › Figure 4G. shCtl cells, EdU.jpg]

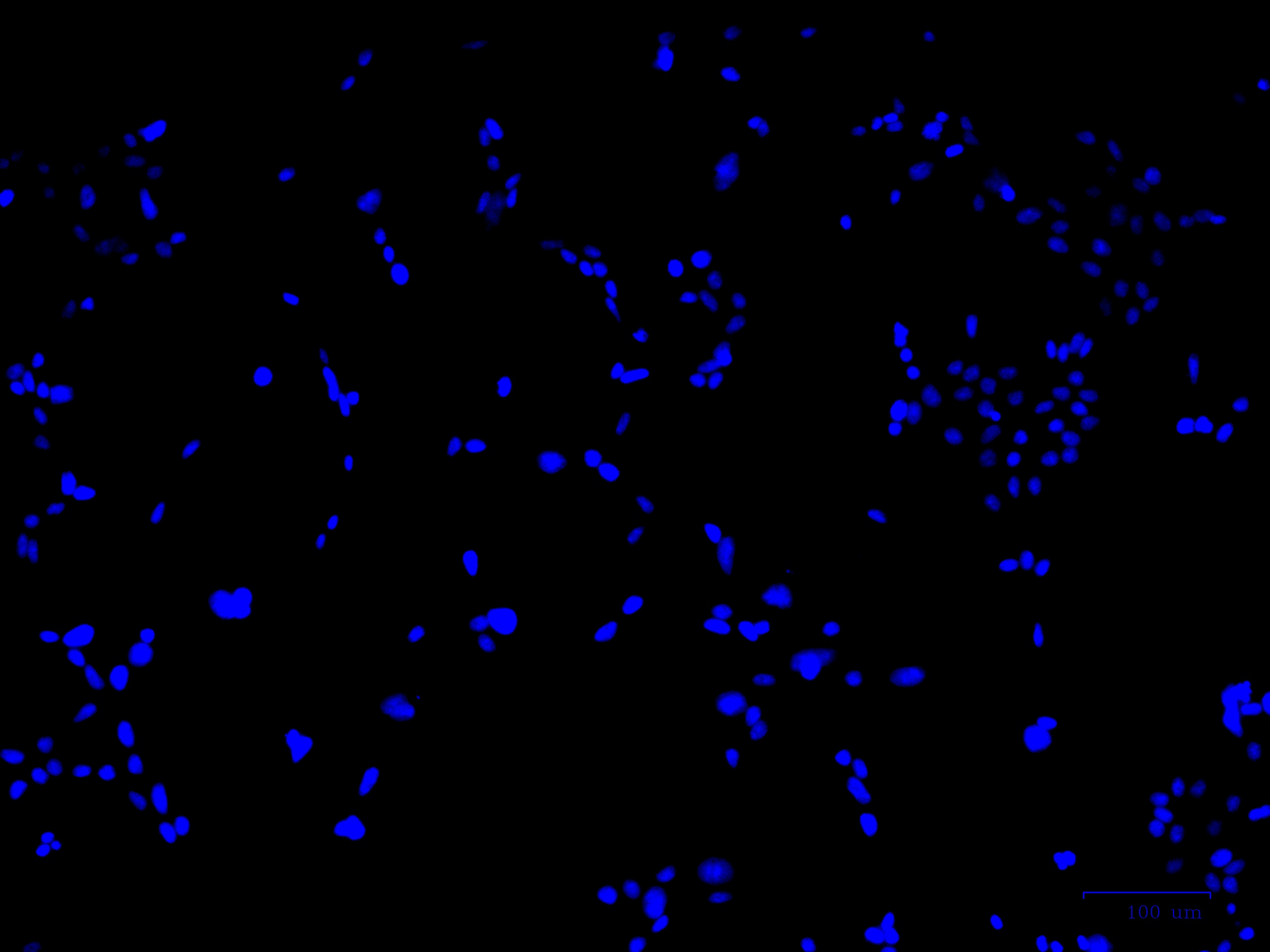

Supplement: S1 File — This file includes the original images underlying Fig 4G; Representative images of DAPI- and EdU-stained FLC cells. (ZIP) [file pgen.1012054.s001.zip › Figure 4G. LeGO-473ox cells, DAPI.jpg]

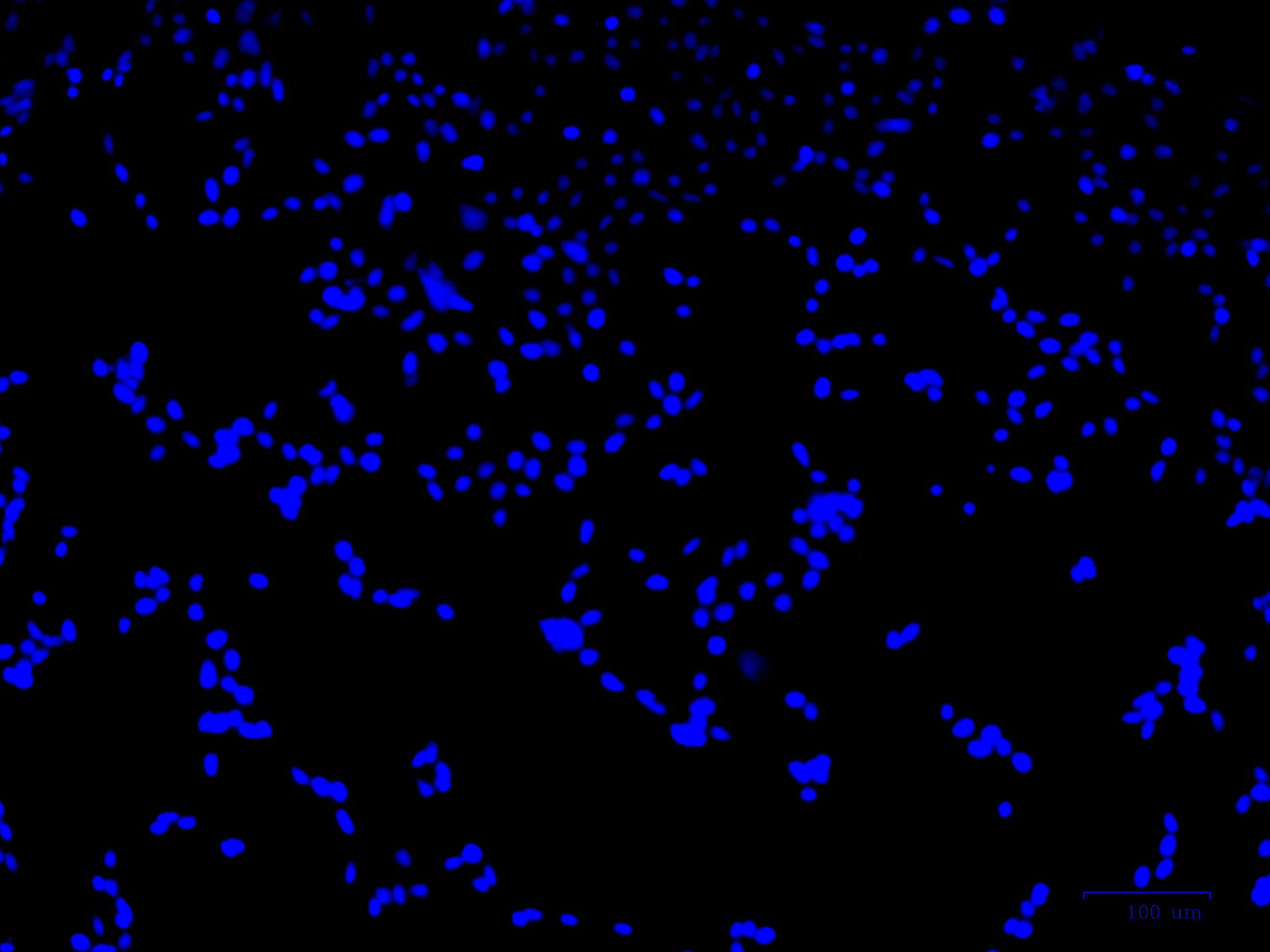

Supplement: S2 File — This file includes the original images underlying Fig 4H; Representative images of DAPI- and TUNEL-stained FLC cells. (ZIP) [file pgen.1012054.s002.zip › Figure 4H. LeGO-473ox cells, DAPI.jpg]

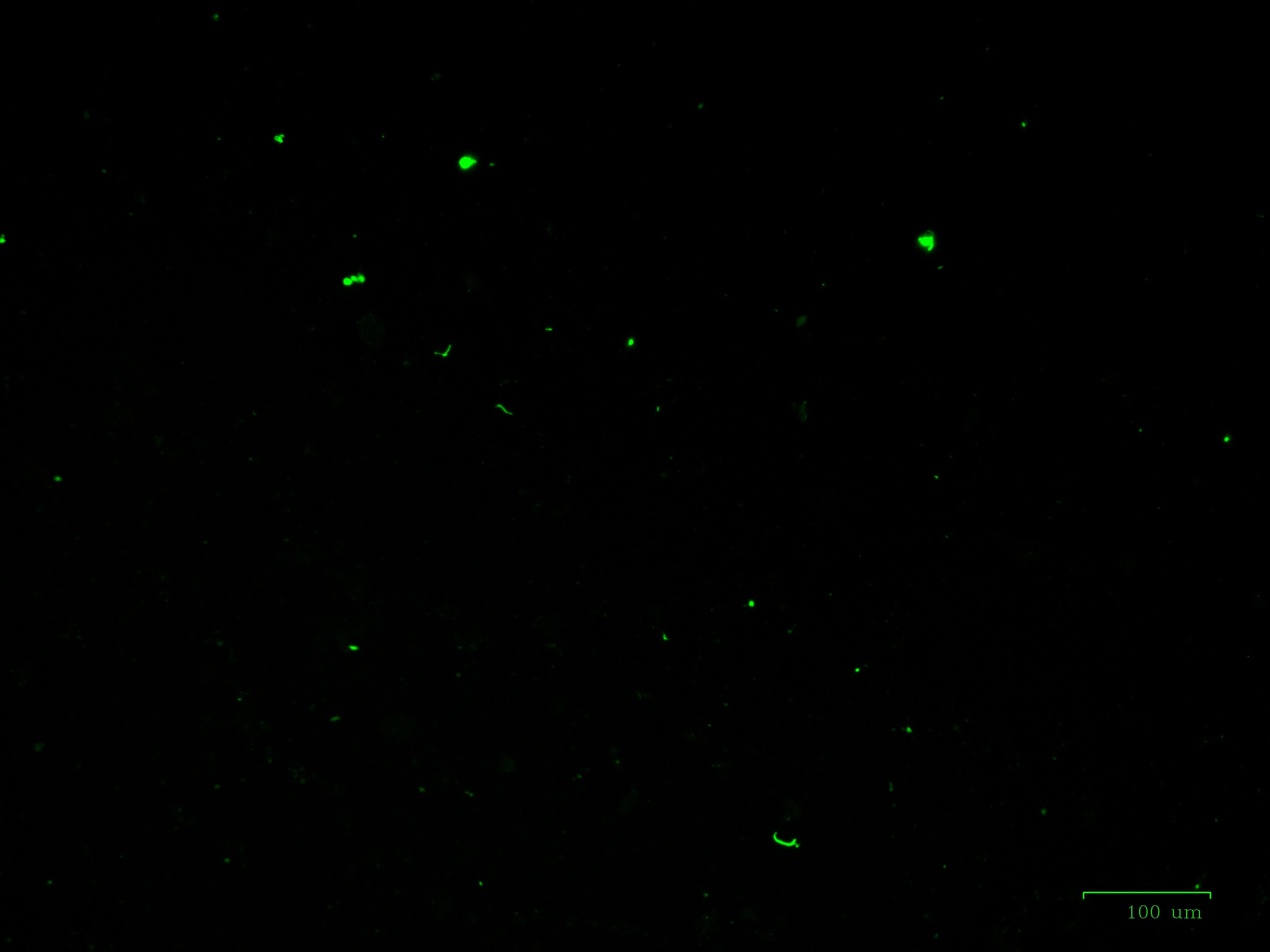

Supplement: S2 File — This file includes the original images underlying Fig 4H; Representative images of DAPI- and TUNEL-stained FLC cells. (ZIP) [file pgen.1012054.s002.zip › Figure 4H. LeGO-473ox cells, TUNEL.jpg]

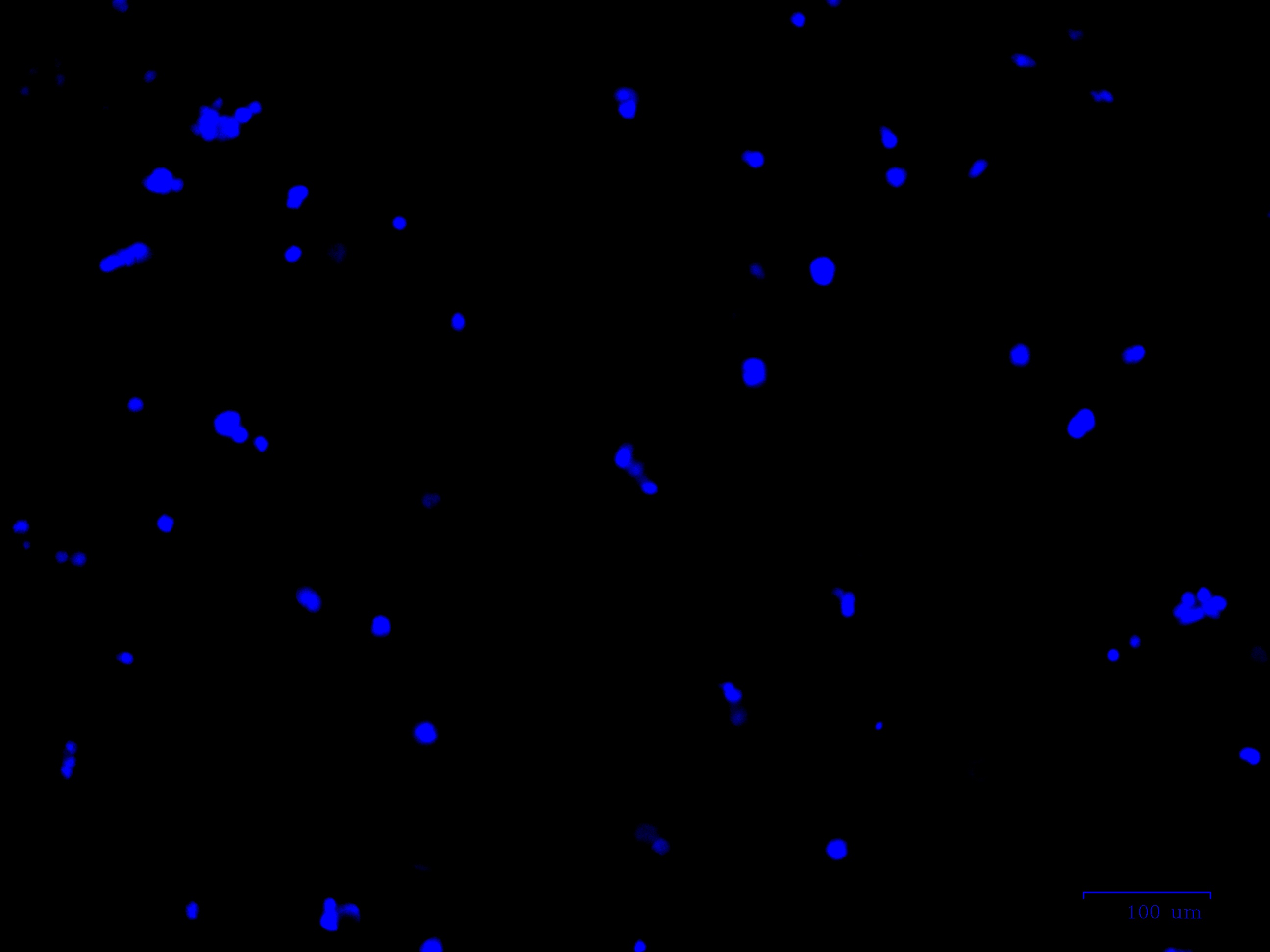

Supplement: S2 File — This file includes the original images underlying Fig 4H; Representative images of DAPI- and TUNEL-stained FLC cells. (ZIP) [file pgen.1012054.s002.zip › Figure 4H. LeGO-Ctl cells, DAPI.jpg]

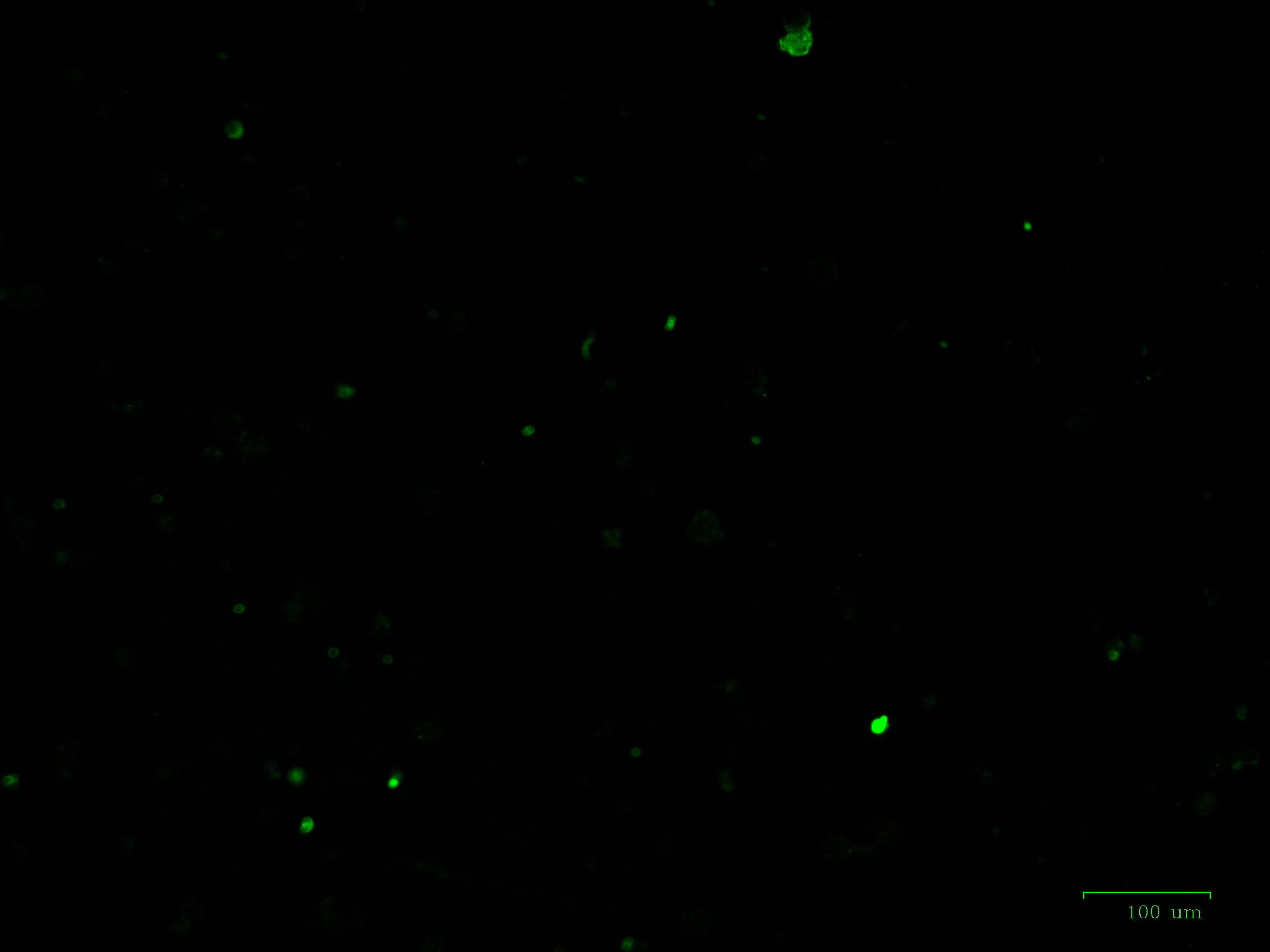

Supplement: S2 File — This file includes the original images underlying Fig 4H; Representative images of DAPI- and TUNEL-stained FLC cells. (ZIP) [file pgen.1012054.s002.zip › Figure 4H. LeGO-Ctl cells, TUNEL.jpg]

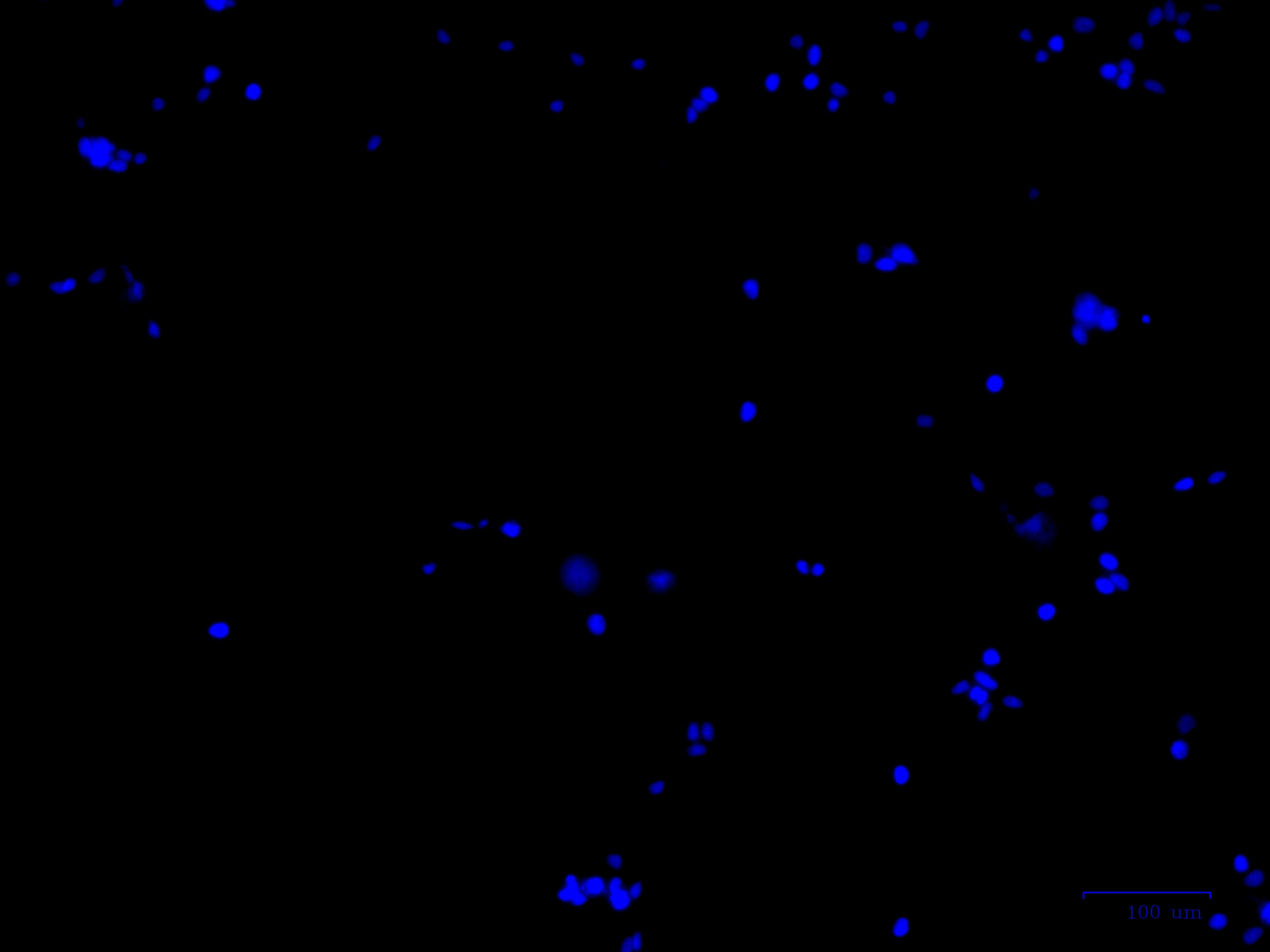

Supplement: S2 File — This file includes the original images underlying Fig 4H; Representative images of DAPI- and TUNEL-stained FLC cells. (ZIP) [file pgen.1012054.s002.zip › Figure 4H. sh473 cells, DAPI.jpg]

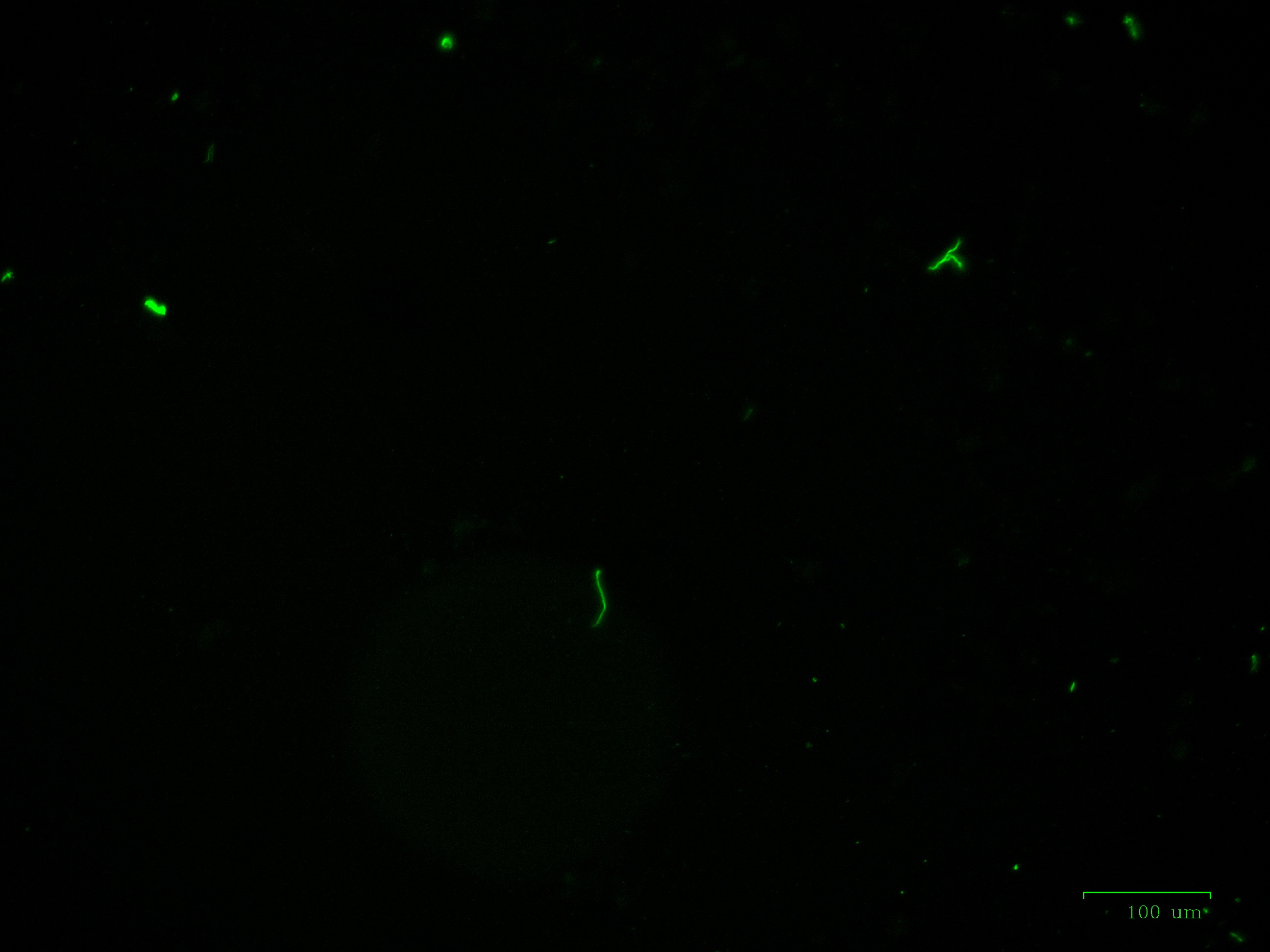

Supplement: S2 File — This file includes the original images underlying Fig 4H; Representative images of DAPI- and TUNEL-stained FLC cells. (ZIP) [file pgen.1012054.s002.zip › Figure 4H. sh473 cells, TUNEL.jpg]

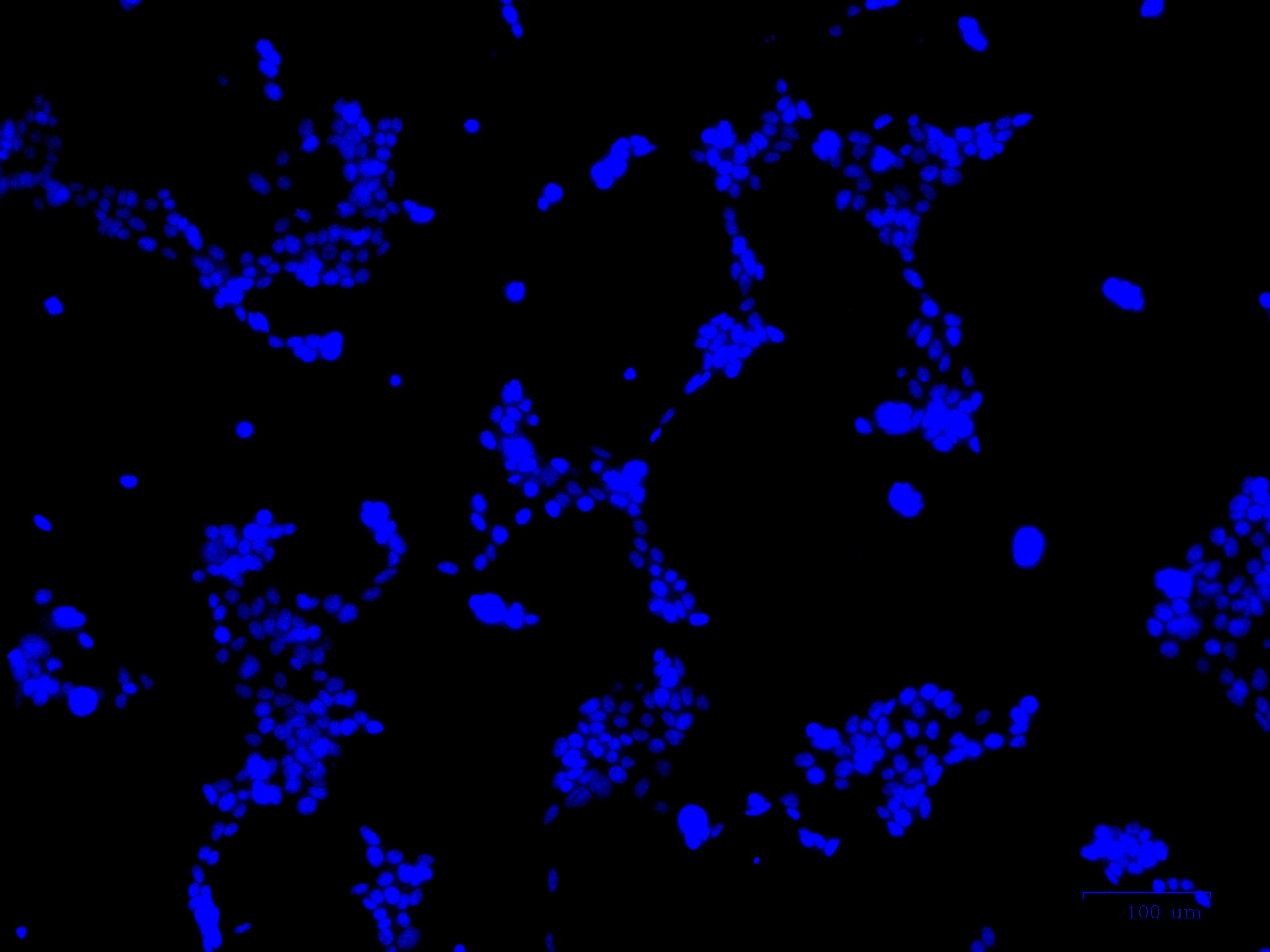

Supplement: S2 File — This file includes the original images underlying Fig 4H; Representative images of DAPI- and TUNEL-stained FLC cells. (ZIP) [file pgen.1012054.s002.zip › Figure 4H. shCtl cells, DAPI.jpg]

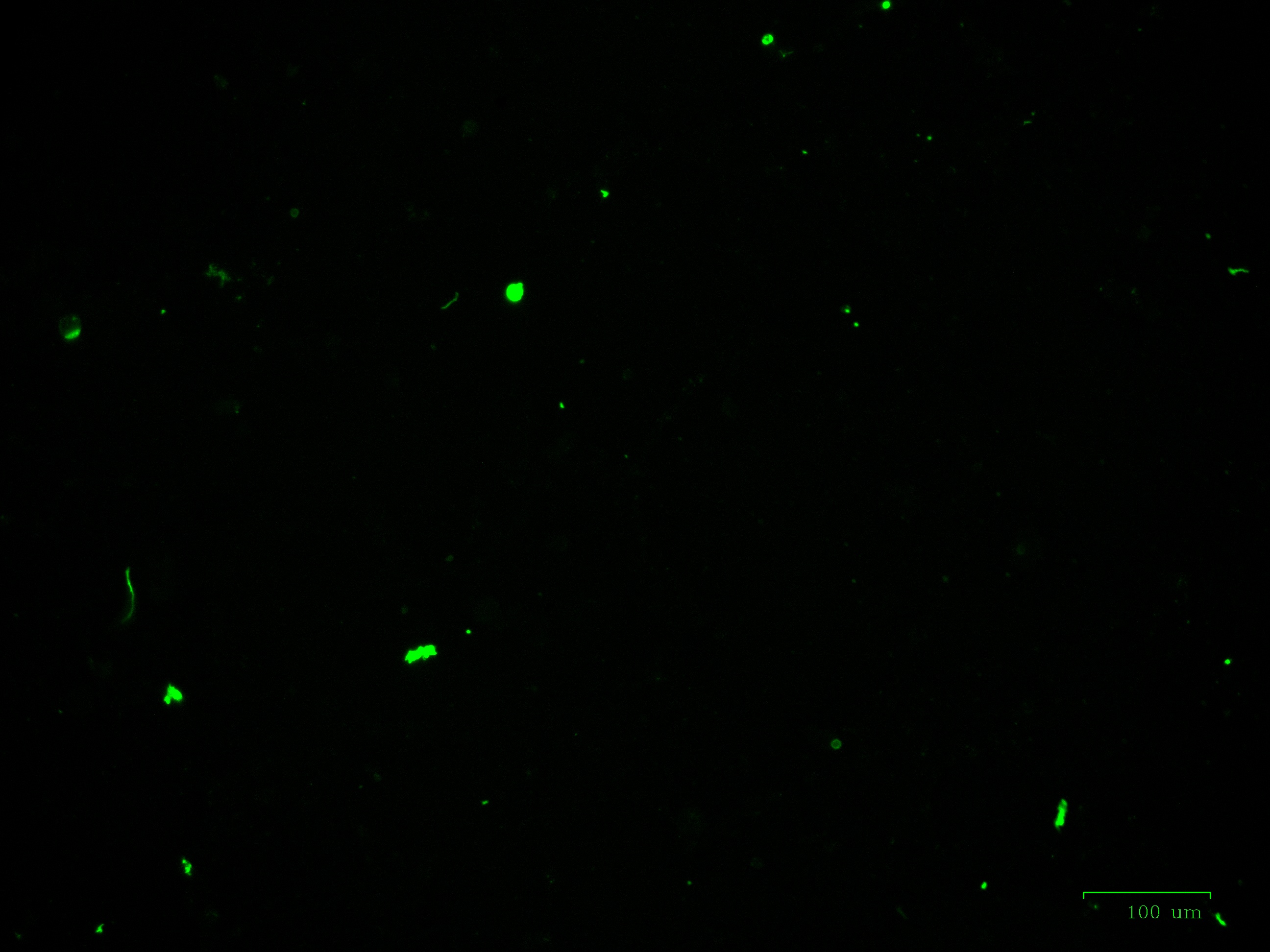

Supplement: S2 File — This file includes the original images underlying Fig 4H; Representative images of DAPI- and TUNEL-stained FLC cells. (ZIP) [file pgen.1012054.s002.zip › Figure 4H. shCtl cells, TUNEL.jpg]

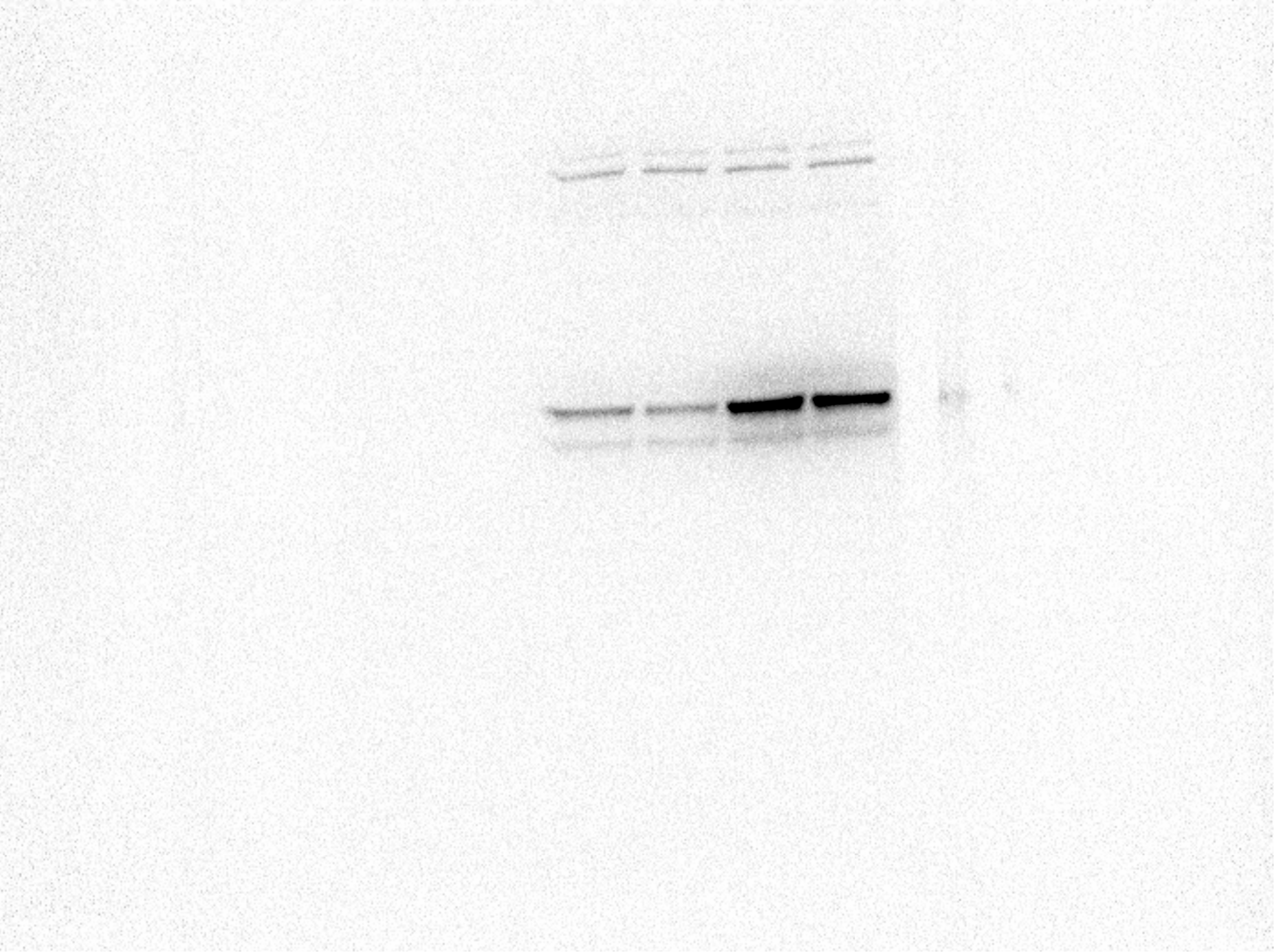

Supplement: S3 File — This file includes the original images underlying Fig S3C; Representative immunoblot of protein expression of DNAJB1- PRKACA (DP) fusion is detected with a protein kinase A catalytic α subunit (PKA) antibody. Lane 1, siDP#1-LNP; Lane 2, siDP#2-LNP; Lane 3, siLuciferase (siLuc-LNP) negative control; Lane 4, mock negative controls following 250nM treatment. (ZIP) [file pgen.1012054.s003.zip › Figure S3C. Immunoblot of DNAJB1-PRKACA and WT PKAc.tif]

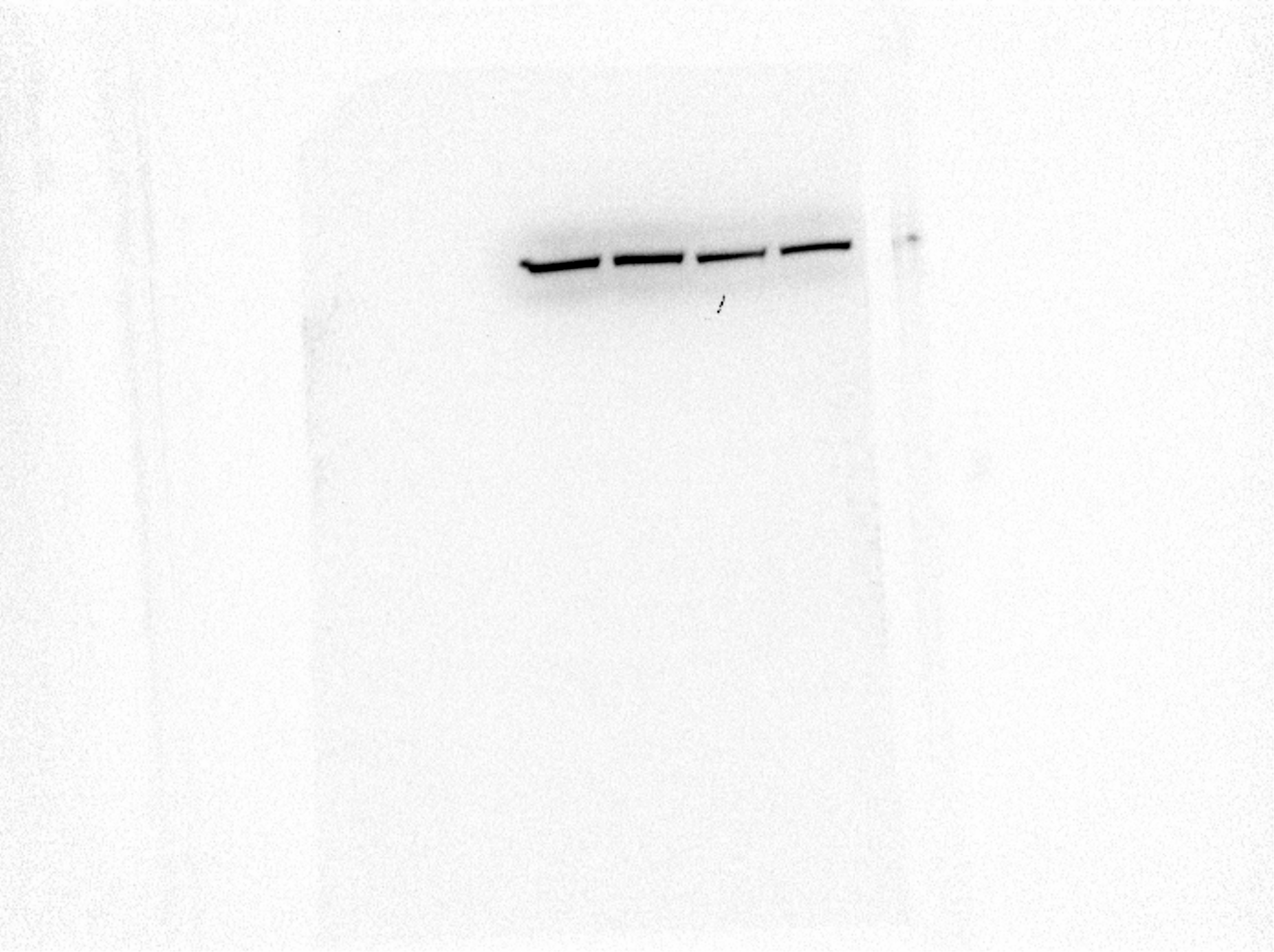

Supplement: S3 File — This file includes the original images underlying Fig S3C; Representative immunoblot of protein expression of DNAJB1- PRKACA (DP) fusion is detected with a protein kinase A catalytic α subunit (PKA) antibody. Lane 1, siDP#1-LNP; Lane 2, siDP#2-LNP; Lane 3, siLuciferase (siLuc-LNP) negative control; Lane 4, mock negative controls following 250nM treatment. (ZIP) [file pgen.1012054.s003.zip › Figure S3C. Immunoblot of vinculin control.tif]

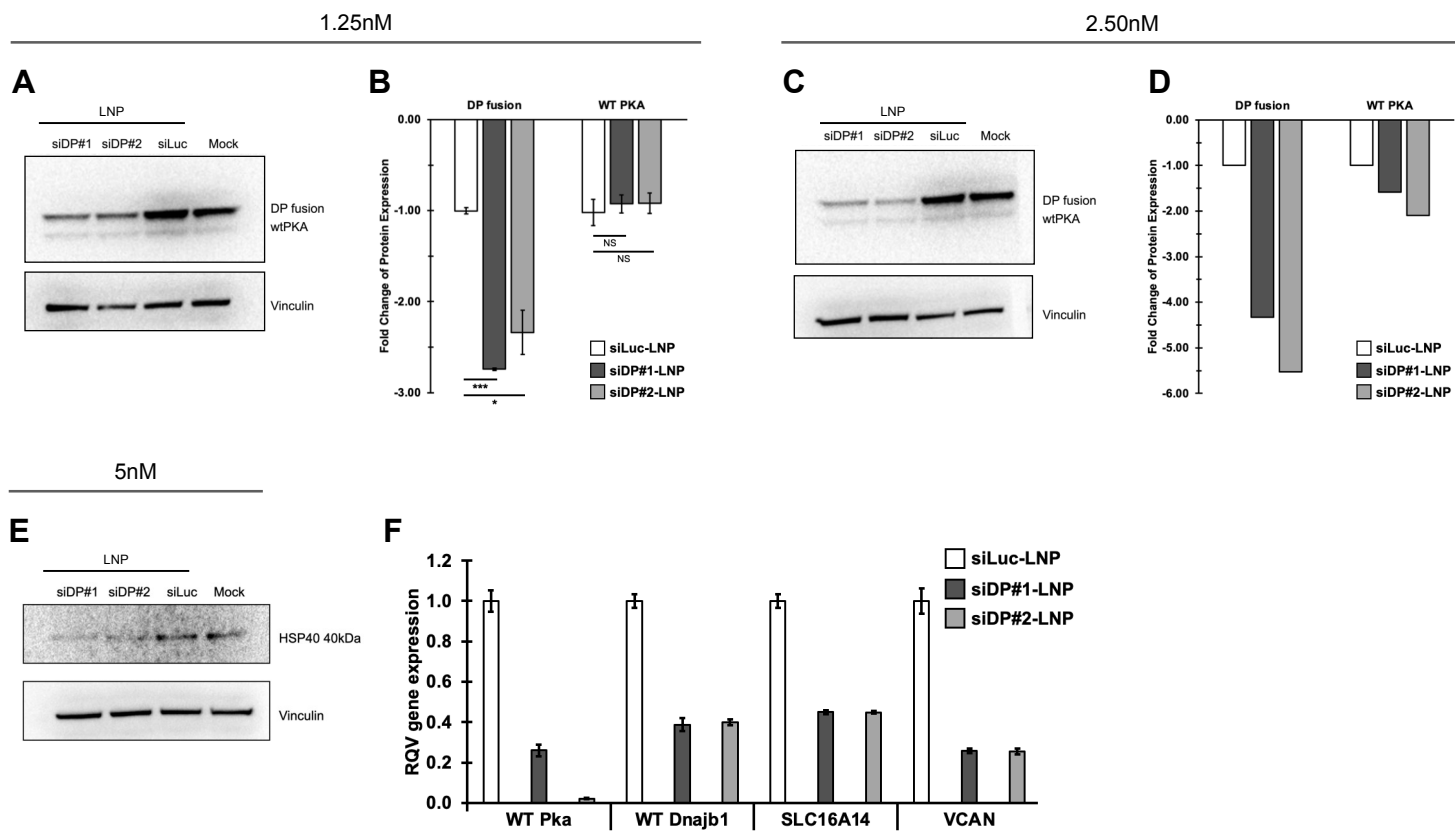

Supplementary Figure 3. Silencing of the DP fusion.

Supplement: S3 Fig — (A, C) Representative immunoblot of protein expression of DNAJB1- PRKACA (DP) fusion is detected with a protein kinase A catalytic α subunit (PKA) antibody. WT PKAc, DP fusion major, and DP fusion minor are identified. Lane 1, siDP#1-LNP; Lane 2, siDP#2-LNP; Lane 3, siLuciferase (siLuc-LNP) negative control; Lane 4, mock negative controls following 1.25nM treatment (A) or 2.50nM treatment (C) with siRNA-LNPs or mock condition over 96 hours. Vinculin loading control is shown in the lower panel and run on the same blot. (B, D) Fold change of protein levels of the blot in panel A (B) and panel C (D), relative to siLuc negative control (n = 1). (E) Representative immunoblot of protein expression of WT DNAJB1. Lane 1, siDP#1-LNP; Lane 2, siDP#2-LNP; Lane 3, siLuciferase (siLuc-LNP) negative control; Lane 4, mock negative control. siRNA-LNP treatments at 5nM, or mock condition, over 96 hours. Vinculin loading control is shown in the lower panel and run on the same blot (n = 3). (F) Gene expression from RT-qPCR following free uptake of siDP#1-LNP, siDP#2-LNP, and siLuc-LNP at 5nM treatment over 96 hours in FLC cells, as shown in Fig 3F (n = 3). Data are represented as mean ± SD. P values are calculated by 2-tailed Student’s t-test. *p < 0.05, **p < 0.01, ***p < 0.001. (PDF) [file pgen.1012054.s008.pdf]
